# Reproductive, maternal, newborn, and child health intervention coverage in 70 low-income and middle-income countries, 2000–30: trends, projections, and inequities

**DOI:** 10.1016/S2214-109X(23)00358-3

**Published:** 2023-09-04

**Authors:** Md Mizanur Rahman, Thomas Rouyard, Sumaiya Tasneem Khan, Ryota Nakamura, Md Rashedul Islam, Md Sifat Hossain, Shamima Akter, Maria Lohan, Moazzam Ali, Motohiro Sato

**Affiliations:** a Research Centre for Health Policy and Economics, Hitotsubashi University, Tokyo, Japan; b Graduate School of Economics, Hitotsubashi University, Tokyo, Japan; c Tokyo Foundation for Policy Research, Tokyo, Japan; d Global Public Health Research Foundation, Dhaka, Bangladesh; e School of Nursing and Midwifery, Queen's University Belfast, Belfast, Northern Ireland, UK; f Department of Sexual and Reproductive Health and Research, WHO, Geneva, Switzerland

## Abstract

**Background:**

Monitoring the progress in reproductive, maternal, newborn, and child health (RMNCH) using the composite coverage index (CCI) is crucial to evaluate the advancement of low-income and middle-income countries (LMICs) towards the attainment of Sustainable Development Goal target 3. We present current benchmarking for 70 LMICs, forecasting to 2030, and an analysis of inequities within and across countries.

**Methods:**

In this cross-sectional secondary data analysis, we extracted 291 data points from the WHO Equity Monitor, and Demographic and Health Survey Statcompiler for 70 LMICs. We selected countries on the basis of whether they belonged to LMICs, had complete information about the predictors between 2000 and 2030, and had at least one data point related to CCI. CCI was calculated on the basis of eight types of RMNCH interventions in four domains, comprising family planning, antenatal care, immunisations, and management of childhood illnesses. This study examined CCI as the main outcome variable. Bayesian hierarchical models were used to estimate trends and projections of the CCI at regional and national levels, as well as the area of residence, educational level, and wealth quintile.

**Findings:**

Despite progress, only 18 countries are projected to reach the 80% CCI target by 2030. Regionally, CCI is projected to increase in all regions of Asia (in southern Asia from 51·8% in 2000 to 89·2% in 2030; in southeastern Asia from 58·8% to 84·4%; in central Asia from 70·3% to 87·0%; in eastern Asia from 76·8% to 82·1%; and in western Asia from 56·5% to 72·1%), Africa (in sub-Saharan Africa from 46·3% in 2000 to 72·2% in 2030 and in northern Africa from 55·0% to 81·7%), and Latin America and the Caribbean (from 67·0% in 2000 to 83·4% in 2030). By contrast, southern Europe is predicted to experience a decline in CCI over the same period (70·1% in 2000 to 55·2% in 2030). Across LMICs, CCIs are higher in urban areas, in populations in which women have higher education levels, and in populations with a high income.

**Interpretation:**

Governments of countries where the universal target of 80% CCI has not yet been reached must develop evidence-based policies aimed at enhancing RMNCH coverage. Additionally, they should focus on reducing the extent of existing inequalities within their populations to drive progress in RMNCH.

**Funding:**

Hitotsubashi University and Japan Society for the Promotion of Science.

## Introduction

In the quest to achieve universal health coverage (UHC) by 2030, the delivery of essential reproductive, maternal, newborn, and child health (RMNCH) services to all women and children is of utmost importance. Strengthening RMNCH care services is imperative to reduce maternal and child mortality.^1^ To monitor UHC for RMNCH, appropriate indicators are necessary to assess both the effectiveness and equity of coverage. The Countdown to 2030, a global initiative for tracking country-level progress in RMNCH intervention coverage, proposed the composite coverage index (CCI) in 2008.^2^, ^3^ The CCI reflects the coverage of eight types of RMNCH interventions in four domains: family planning (demand for family planning satisfied through modern methods); antenatal and delivery care (at least four antenatal care visits and skilled birth attendance); immunisation (with BCG, three doses of pentavalent, and measles vaccinations); and management of childhood illnesses (oral rehydration solution for diarrhoea and seeking care at a health facility for suspected pneumonia cases).^4^, ^5^ This index has the advantage of providing a general picture of the level of RMNCH coverage in several key intervention domains using a single measure, enabling the identification of populations that are left behind. More importantly, the index also allows for an analysis of inequity in RMNCH coverage by wealth, area of residence, and education within each country. Because it is both comprehensive and simple, the CCI has been widely used in previous peer-reviewed studies,^4^, ^5^, ^6^, ^7^, ^8^, ^9^ as well as non-peer-reviewed publications including visualisation materials. 10, 11, 12

**Research in context**

**Evidence before this study**

Measuring the coverage of reproductive, maternal, newborn, and child health (RMNCH) services is crucial for monitoring progress towards the Sustainable Development Goals (SDGs) and universal health coverage. The composite coverage index (CCI), based on eight indicators, is a crucial tool in understanding the progress of a country towards achieving SDG target 3 (to ensure healthy lives and promote wellbeing for all at all ages). To assess the existing evidence of RMNCH coverage in low-income and middle-income countries (LMICs), before our study we did a literature search on PubMed. The search covered articles published in any language from the database's inception to Jan 25, 2023, and used the search terms (mother OR maternal OR women OR children OR newborn) AND (maternal and child health service OR maternal health service OR maternal health OR child health service OR health service OR women's health service OR reproductive health service) AND (composite coverage index OR CCI OR coverage index) (appendix p 2). Out of 254 articles identified, 35 articles assessed the CCI in LMICs, and 30 were selected for full-text review (appendix pp 3–4). Despite various attempts to provide information about RMNCH coverage index, particularly the CCI, all of the included studies had limitations. 16 studies were limited to specific countries, whereas the remaining studies often focused on a specific region. We also noted some methodological weaknesses. For example, a 2020 study used all available data to monitor progress and project the RMNCH care-services index across various population subgroups within LMICs. The authors concluded that no countries will achieve the universal CCI (100%) by 2030, with only six countries projected to have over 90% CCI. However, the study did not consider any confounding variables in the model and lacked CCI estimation at the regional level. Generating up-to-date CCI estimates and projections up to 2030 (ie, for the remaining 7 years of the SDG era) is now crucial to identify which populations are being left behind. This information will enable health promotion planners to design better-targeted and equitable policies for efficient resource distribution.

**Added value of this study**

This study represents the most comprehensive and up-to-date evaluation of the progress in RMNCH coverage in LMICs towards meeting the SDG 3.8 target of 80% health service coverage by 2030. The Article makes important contributions in two ways. First, it provides a robust time-trend analysis of key RMNCH indicators in a large sample of 70 LMICs to forecast projections of coverage by 2030, both at the national and regional levels, and includes confidence estimates for the forecasts. Second, it investigates the inter-relationships among the trends over time at regional, national, and subnational levels by considering key factors such as socioeconomic status, maternal educational status, and area of residence using a Bayesian hierarchical model. This analysis is essential for governments and international organisations to guide optimal interventions and drive future progress to where it is most needed.

**Implications of all the available evidence**

Our study found that overall RMNCH service coverage has increased from 2000 to 2020, but only 18 countries are projected to reach the 80% coverage target by 2030. Surprisingly, the regional analysis revealed that southern Europe is the only region currently declining in relation to targets. Despite projections of reduced wealth-based, educational, and urban–rural disparities in most countries by 2030, our forecasts indicate that these disparities will persist. Systemic interventions including investment in the education of girls and women, provision of health services in rural areas, and investment in universal outpatient coverage are expected to result in a global benefit of enhanced RMNCH coverage.

Most of the previous studies reported evidence of persistent disparities in RMNCH coverage by wealth, place of residence, and maternal education.^4^, ^6^, ^7^, ^8^, ^9^ Despite several attempts to provide information about the RMNCH coverage index, particularly the CCI, these studies limited their scope to specific countries or regions. Additionally, none have examined trends and projections of the CCI at the national and regional levels, specifically considering various equity strata such as wealth quintiles, educational levels, and geographical regions. To address this gap, the Countdown to 2030 project has been established with the goal of enhancing collaboration among academics and policy makers. The project aims to facilitate the production of comprehensive analyses on population coverage with essential health interventions, with a specific focus on identifying inequalities within countries.^2^, ^3^ With only 7 years left in the Sustainable Development Goals era, it is crucial that low-income and middle-income countries (LMICs) and international organisations invest in, implement, expedite, and scale up strategies to overcome these inequalities and reach the universal 80% CCI target by 2030. In this context, updating CCI estimates and projections up to 2030 using the latest available data is essential to identify populations that are being left behind, thereby enabling health promotion planners to design more equitable and better-targeted policies for efficient resource distribution.

The present study aimed to determine the extent to which LMICs have succeeded in reducing inequalities in RMNCH coverage over the past two decades and to forecast future trends up to 2030. Specifically, the objectives of the study were to estimate recent trends in CCI and develop projections up to 2030 for 70 LMICs at regional and national levels and assess the magnitude of inequality gaps in CCI by area of residence, education, and wealth from 2000 to 2030.

## Methods

### Data sources

In this cross-sectional secondary data analysis, data were extracted from the WHO Equity Monitor and Demographic Health Survey (DHS) Statcompiler. The WHO Equity Monitor database was derived from both the DHS and Multiple Indicator Cluster Survey (MICS), both cross-sectionally representative surveys conducted primarily in LMICs. The two surveys used similar multistage cluster sampling methods to select women of reproductive age (15–49 years) and children younger than 5 years for inclusion, making them highly comparable. Countries are grouped into regions according to the UN classification system. Using the World Bank 2022 income classification, country income groups were determined by their gross national income per capita. We selected countries on the basis of whether they belonged to LMICs, had complete information about the predictors between 2000 and 2030, and had at least one data point related to CCI. These criteria led us to include 70 LMICs in our study. In 20 countries, there was only one data point, three countries had a survey dating back more than 10 years, and the remainder had more than three data points. Detailed research in context and data are provided in the appendix (pp 2–6).

### Outcome variable

Countdown to 2015 developed the CCI to measure interventions for RMNCH, stratifying by variables such as education level, wealth quintile, and residence (urban or rural).^2^, ^13^ In surveys such as the DHS and MICS, these variables are routinely collected in most LMICs. CCI reflects the weighted average of four domains of RMNCH interventions (family planning, maternity care, child immunisation, and case management). This index was calculated as:

$$\begin{array}{c} CCI=1/4(DFPSm+(\frac{ANC4+SBA}{2}+\frac{BCG+2\times DPT3+MSL}{2}\\ +\frac{ORS+CAREP}{2}) \end{array}$$

where DFPSm is demand for family planning satisfied with modern methods, ANC4 is four or more antenatal care visits with any provider, SBA is skilled birth attendant, BCG is bacille Calmette–Guérin vaccine, DPT3 is three doses of the diphtheria, pertussis, and tetanus vaccine, MSL is measles immunisation, ORS is oral rehydration salts, and CAREP is careseeking for suspected pneumonia. The details of the CCI computation are provided in the appendix (pp 7–8).

### Independent variables

Following previous studies,^14^, ^15^, ^16^, ^17^ we also considered several country-level factors, such as the sociodemographic index (SDI), development assistance for health (DAH), human resources for health (HRH), government gross domestic product (GDP) spending on health (GGDPH), and GDP per capita (GDPC). The details of the predictor variables are provided in the appendix (p 9).

### Statistical analysis

We used Bayesian hierarchical linear regression models to estimate the trends in CCIs over time, country, region, area of residence, wealth quintile, and maternal education level. The Bayesian approach offers several benefits, including the ability to incorporate previous knowledge of parameters with new data to enhance the estimation of parameters and reducing the potential biases associated with small sample sizes and model misspecifications. Additionally, Bayesian models enable a more straightforward interpretation of the calculated estimates and associated uncertainties, presenting credible intervals (CrIs) instead of CIs. The model was used to predict the posterior distribution of logit-transformed CCI and calculate the probability of reaching the UHC target of 80% coverage by 2030. The outcome variable was logit transformed to ensure that the predicted value decreased within the probability range of 0% to 100%. Specifically, the following basic model was used to estimate the trends in and projections of CCI up to 2030, at the national, urban, and rural levels:

$$\begin{array}{l} y_{ijkl}\sim N\left(X\beta_{ijk},\tau^{2}\right)\\ y_{ijkl}=\alpha_{ijk}+\beta_{ijk}year_{ijk}+\beta_{2,ijk}residence_{ijk}+\beta_{3,jk}SDI_{ijk}+\beta_{4,jk}GGDPH_{ijk}\\ +\beta_{5,jk}GDPC_{ijk} \end{array}$$

where *y*_ijkl_ is the logit-transformed probability corresponding to the CCI in the *i*th year for the *j*th country in the *k*th region. The model incorporated covariates that aid in predicting CCI, including year, residence area, and time-varying country-level factors such as SDI, GGDPH, and GDPC. Note that *residence*_ijk_={*National*_ijk_, *Urban*_ijk_, *Rural*_ijk_} represents national, urban, and rural levels and is encoded as a dummy variable. After estimating the regression parameters, we can predict the CCI at the national, urban, and rural levels. The term *a*_ijk_ gives the random intercept. The error term ɛ_ijk_ quantifies random variations in CCI that are not explained by the covariates. We also incorporated interactions of residence with country to account for potential changes in associations over time, particularly in relation to the availability of treatments for maternal and child health.

To explore disparities in CCI based on wealth, the basic model was extended to estimate the quintile-specific CCI using the same country-level covariates. The wealth quintile model incorporates the following variables: year, wealth quintile, SDI, GGDPH, and GDPC. The wealth quintiles are categorised into five groups, from the lowest-income (Q1) to the highest-income (Q5). Note that *wealth*_ijk_={*Q*1_ijk_, *Q*2_ijk_, *Q*3_ijk_, *Q*4_ijk_, *Q*5_ijk_} is represented by dummy variables encoding each wealth quintile from Q1 to Q5. A similar methodology was used to explore disparities in CCI based on maternal education levels. Detailed information about the Bayesian models and corresponding parameters is provided in the appendix (pp 10–11).

Initially, we established a baseline model that included several predictor variables such as SDI, DAH, HRH, GGDPH, and GDPC. We then applied variation inflation factor (VIF) analysis to identify any potential multicollinearity. Variables with VIFs greater than 5 were subsequently eliminated one by one. Furthermore, we applied the deviance information criteria (DIC) to calculate DIC values for each model. Finally, we conducted posterior predictive checks to assess the model fit. We compared the predicted and true proportions of the simulated CCI proportion for every Markov chain Monte Carlo iteration. We examined the sensitivity of our results by altering priors for the hyperparameters. Since the hyperparameter has some influence on all intercept coefficients, the half-Cauchy distribution (weekly informative prior) was applied to perform sensitivity analysis instead of gamma distribution, hyperparameters (τ, αk, and α) approximately half-Cauchy (centre 0, scale 25). After altering prior distribution, the median absolute differences between the two sets of results were also compared. Model diagnosis was performed using trace plots and potential scale reduction factors (PSRFs). Convergence was interpreted as PSRF values near 1·0. We calculated the difference between the predicted values and the true values used to generate the data. The final Bayesian model for CCI prediction used SDI, GGGPH, and GDPC as potential predictor variables based on previous studies,^14^, ^15^, ^16^, ^17^ lowest DIC value (appendix p 11), and posterior predictive check p value (appendix p 11). To obtain estimates and projections for country aggregates, such as regions, we calculated weighted averages of the constituent country-level estimates. We used a random effects meta-analysis to derive regional level averages. To calculate the mean and 95% CrIs for the regional level model, we used the posterior samples.

We measured wealth-based and education-based inequalities in coverage by using the slope index of inequality (SII). We calculated the SII using a regression model that uses the natural logarithm of the proportion of the dependent variable, in our case CCI, to create a continuous criterion on which linear regression is done with the wealth index or level of education as an explanatory variable. Statistical analysis was done using R 4.2.0 and Stata version 17MP.

### Ethics approval

This manuscript is based on secondary data that are already freely available. Both the DHS and MICS surveys were done in compliance with the ethical requirements of the respective institutions, which ensured informed consent (for children, consent was given by their caregivers) and confidentiality of the respondents' information. Analyses were done using anonymised, publicly accessible databases. Consequently, the authors of this manuscript were not required to obtain any further ethics clearance for their research.

### Role of the funding source

The funders of the study had no role in study design, data collection, data analysis, data interpretation, or writing of the report.

## Results

On the basis of our posterior predictive check, we found p values near 0·5, indicating that there was no absence of fit in the model (appendix p 12). We did a sensitivity analysis by altering the priors of the hyperparameters. After altering the prior distribution from a γ distribution to a half-Cauchy distribution, the results did not vary significantly (appendix pp 13–14). Regarding model diagnostics, the PSRF values indicate that the point estimate and upper limit of the PSRF for the model were close to 1 (appendix pp 15–16).

**Figure 1:**
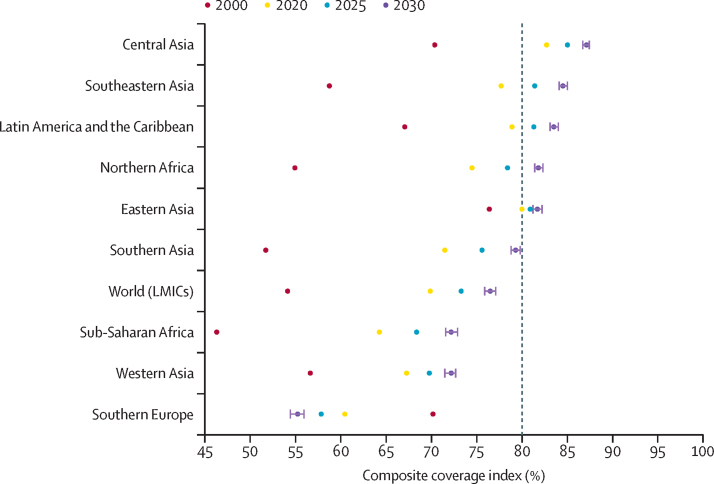
Estimated composite coverage index at the regional level from 2000 to 2020 and projections for 2025 and 2030 Error bars are 95% CIs. Vertical dashed line are universal targets by 2030. LMICs=low-income and middle-income countries.

The predicted CCIs for each region are presented in figure 1. Over the past two decades, the overall coverage of all 70 LMICs has increased. Although this positive trend is expected to continue, projections indicate that the UHC target of 80% coverage will not be reached by 2030. In 2020, only two of the nine regions (central Asia and eastern Asia) were estimated to have reached 80% coverage. On the basis of current trends, three additional regions were predicted to be on track to meet this universal target by 2030 (southeast Asia, 84·4%; northern Africa, 81·7%; and Latin America and the Caribbean, 83·4%). However, it is worth noting that southern Europe was estimated to have exhibited a significant decline in coverage over time, from 70·1% in 2000 to 55·2% in 2030.

**Figure 2:**
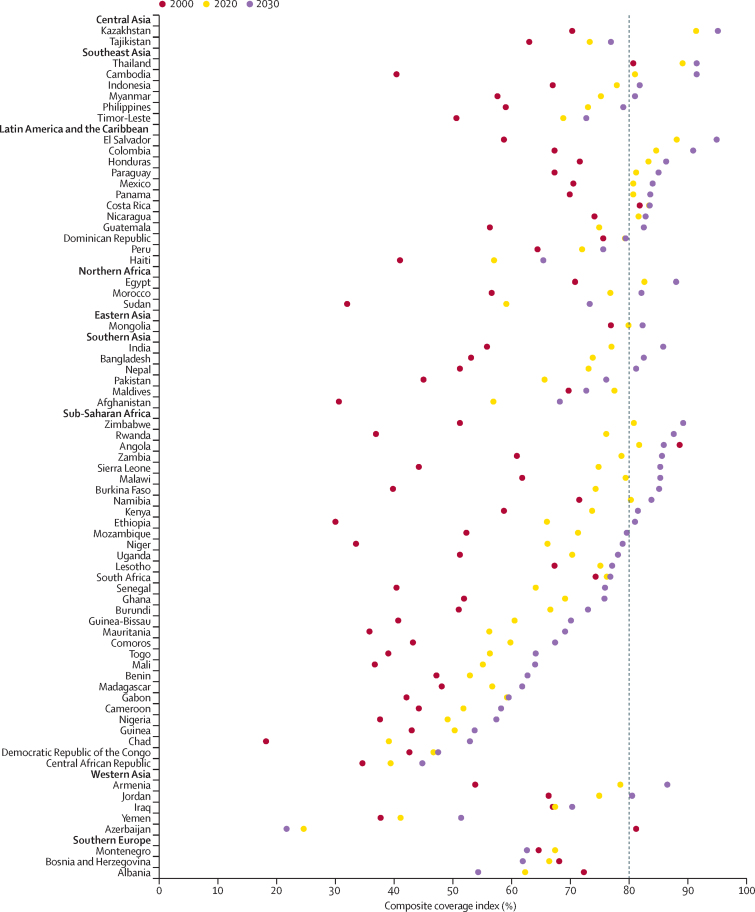
Estimated composite coverage index at the national level from 2000 to 2020 and projection from 2025 to 2030 Regions are rank ordered by predictions for meeting 2030 composite coverage index target coverage from highest to lowest, and nations within regions are rank ordered by 2030 projections. Vertical dashed line=universal target by 2030.

Our projections suggest that 29 of the 70 LMICs will be predicted to reach the 80% CCI target at the national level by 2030, with a probability of at least 70% (figure 2). The country-specific predicted CCIs from 2000 to 2030 are shown in the appendix (pp 17–20). Despite noticeable improvements in CCI worldwide over the past two decades, there are still large disparities between countries. For example, the CCI in Azerbaijan is projected to decrease from 78·3% in 2000 to 25·5% in 2030, whereas the CCI in Ethiopia is expected to increase significantly from 29·9% to 80·8% over the same period. The performance landscape is also changing. In 2000, Chad (18·5%) was the lowest-performing country and Angola (89·3%) the highest-performing country, but by 2030, Azerbaijan (25·5%) and El Salvador (95·5%) are expected to set new extremes.

**Figure 3:**
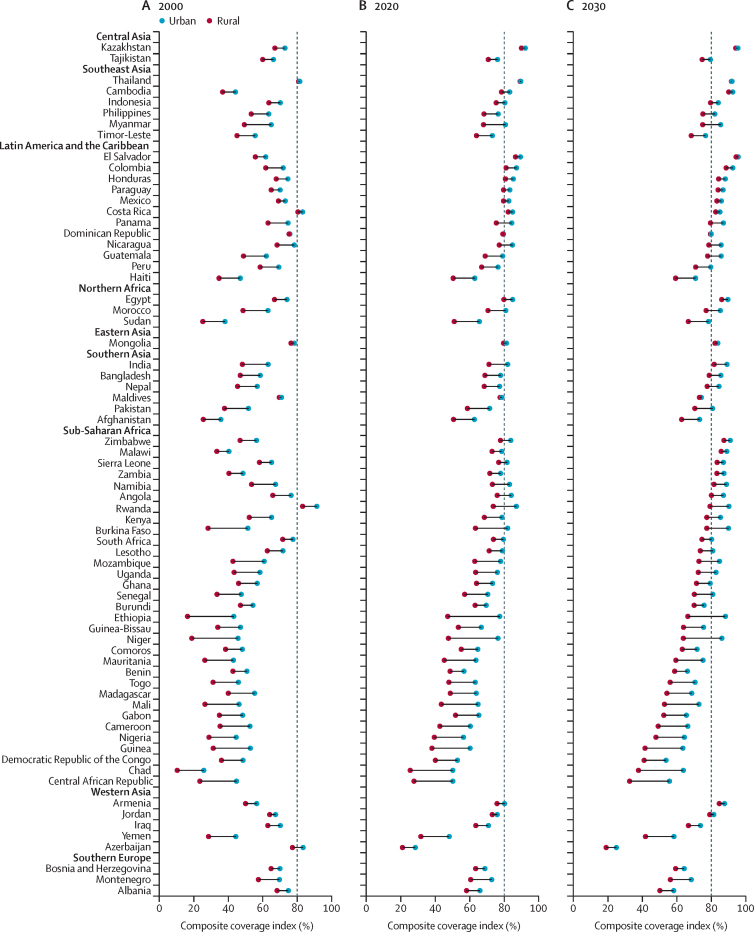
Estimated composite coverage index by place of residence Regions are rank ordered by predictions for meeting 2030 composite coverage index target coverage from highest to lowest, and nations are rank ordered within regions based upon 2030 projections. Vertical dashed lines=universal target by 2030.

Figure 3 shows the predicted CCIs based on area of residence (urban or rural) for each country for the years 2000, 2020, and 2030. The detailed tabulated values can be found in the appendix (pp 21–32). Most countries recorded improvements in CCIs in both urban and rural areas between 2000 and 2020. In 2000, the CCI ranged from 26% to 92% in urban areas and from 10% to 84% in rural areas; however, in 2020, the CCI increased to 32–93% in urban areas and 24–90% in rural areas. During these years, some countries made impressive progress, such as Cambodia, Colombia, the Dominican Republic, Egypt, El Salvador, Honduras, Kazakhstan, Mexico, and Paraguay, which reached 80% CCI in both urban and rural areas in 2020, despite not meeting this target in either area in 2000. However, projections for 2030 indicate static CCIs with not much improvement in both the areas (urban 28–96% and rural 22–95%). The projections further suggest that 42 of the 70 LMICs will reach 80% CCI in urban areas by 2030, whereas only 18 countries will reach this target in rural areas.

**Figure 4:**
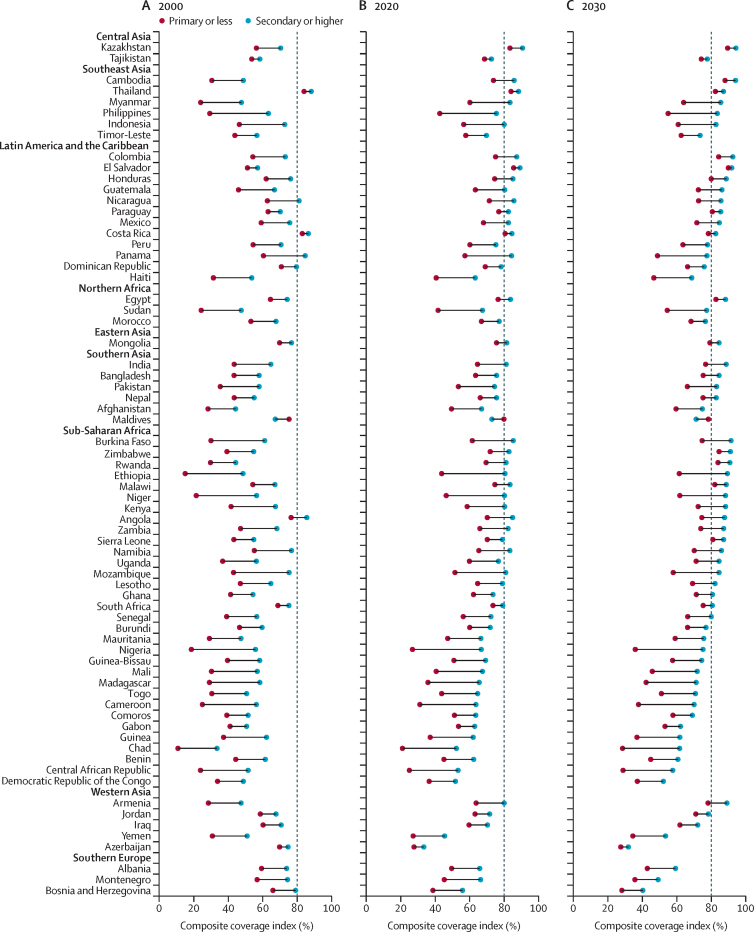
Estimated composite coverage index by maternal education level Regions are rank ordered by predictions for meeting 2030 composite coverage index target coverage from highest to lowest, and nations are rank ordered within regions on the basis of 2030 projections. Vertical dashed lines=universal target by 2030.

The predicted CCIs by maternal education level are displayed in figure 4 and further detailed in the appendix (pp 33–35). The progress towards reaching 80% CCI among women with no education has been slow, with only two countries (Costa Rica and Thailand) reaching this target in 2000, five countries (Costa Rica, El Salvador, Kazakhstan, the Maldives, and Thailand) in 2020, and 12 countries (Cambodia, Colombia, Egypt, El Salvador, Honduras, Kazakhstan, Malawi, Paraguay, Rwanda, Sierra Leone, Thailand, and Zimbabwe) in 2030. By contrast, women with secondary or higher education have shown greater progress, with five countries (Angola, Costa Rica, Nicaragua, Panama, and Thailand) reaching the target in 2000, 28 countries in 2020, and 38 countries in 2030.

**Figure 5:**
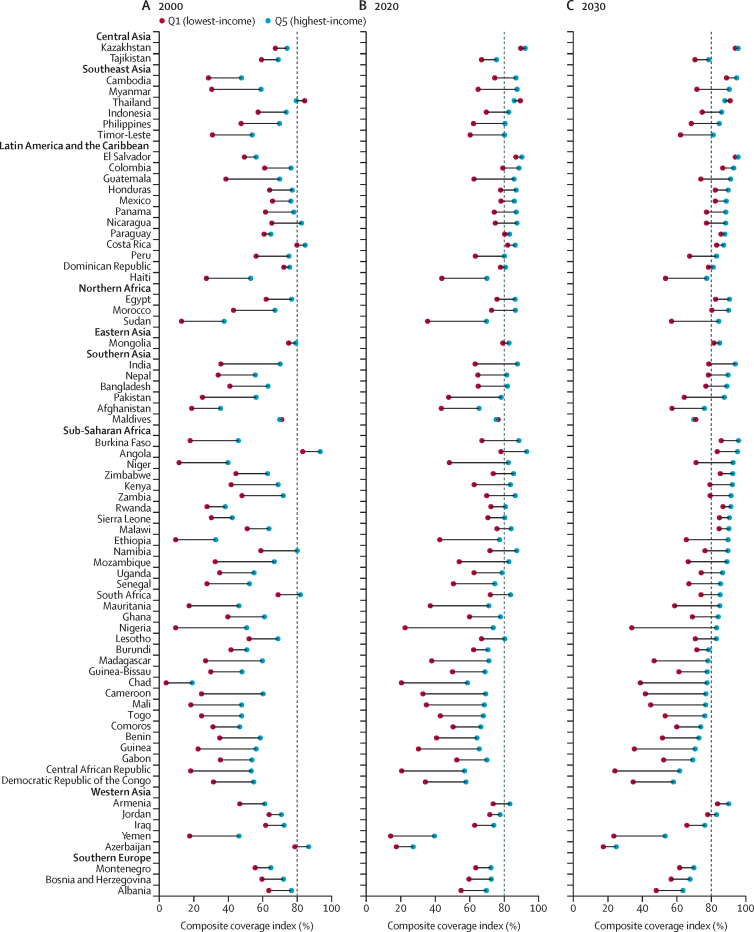
Estimated composite coverage index by wealth quintile Regions are rank ordered by predictions for meeting 2030 composite coverage index target coverage from highest to lowest, and nations are rank ordered within regions by 2030 projections. Vertical dashed lines=universal target by 2030.

The predicted CCIs by wealth quintile are shown in figure 5 and further detailed in the appendix (pp 36–39). In 2000, only five countries (Angola, Azerbaijan, South Africa, Costa Rica, and Nicaragua) reached the 80% target for the highest-income population group, whereas the target was reached in only two countries (Angola and Thailand) for the lowest-income population group. However, over the past two decades, there has been a significant improvement in coverage for the highest-income population group, with 43 countries reaching the target by 2020 and an additional nine projected to reach it by 2030. On the other hand, progress has been slower for the lowest-income population groups, with only four countries (Costa Rica, El Salvador, Kazakhstan, and Paraguay) reaching the 80% target by 2020, but with an additional 14 countries expected to reach it over the next decade. Overall, whereas most countries have shown progress in coverage for both the lowest-income and highest-income groups since 2000, it is worth noting that a few countries have shown a declining trend (Angola, Albania, Azerbaijan, and Bosnia and Herzegovina).

**Figure 6:**
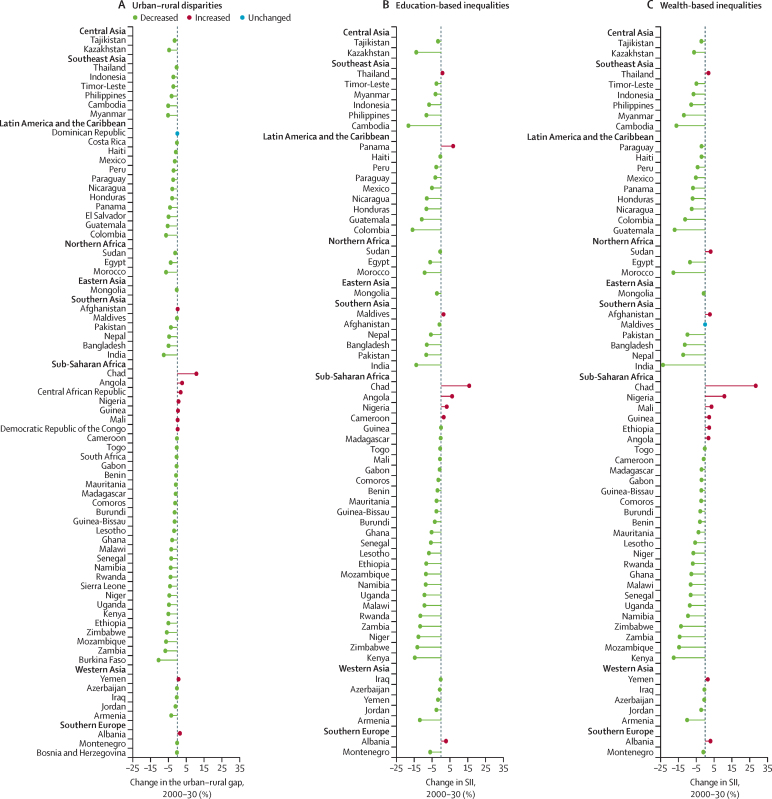
Change in inequalities in composite coverage index from 2000 to 2030 Given that the urban–rural equity stratifier variable is in a nominal scale, percentage difference was used to calculate absolute inequality. Education and wealth quintiles are ordinal variables, so we used a regression model to calculate the SII. SII=slope index of inequality.

The gaps in CCI by area of residence, maternal education level, and wealth quintiles over the period 2000–30 are displayed in figure 6 and in the appendix (pp 40–45) to illustrate the magnitude and evolution of inequalities in access to essential maternal and child health services. Results show that CCI is generally higher in urban areas (figure 6A), with inequality in coverage by area of residence decreasing from 2000 to 2020 and expected to continue to 2030. The data also show that inequalities in coverage by maternal education level are decreasing overall (figure 6B). In 2000, 50 countries exhibited inequalities as high as 20 percentage points between populations in which women were educated and those in which they were not. This number has decreased to 41 percentage points in 2020 and is projected to decrease further to 38 percentage points in 2030. Finally, we observed a similar decreasing trend for wealth-based inequalities in coverage. Although 43 countries exhibited inequalities as high as 20 percentage points in 2000, this number decreased to 35 percentage points in 2020 and is expected to further decrease to 26 percentage points in 2030. In 2030, the CCI projected that the greatest disparities between rich and poor households were found in Nigeria, Chad, Central African Republic, Guinea, Cameroon, Mali, Madagascar, Yemen, Sudan, and Mauritania. Here, the CCI was 30–50 percentage points higher for rich households than for poor households. By contrast, the most equitable figures were found in Kazakhstan, El Salvador, Paraguay, Mongolia, Costa Rica, and the Dominican Republic, with the CCI only 0–5 percentage points higher for rich households than poor households. We predict that 14 countries will continue to perform particularly well at reducing the magnitude of these inequalities between 2000 and 2030, with projected reductions reaching at least 10 percentage points (Armenia, Colombia, Bangladesh, Myanmar, Nepal, Zimbabwe, Zambia, Mozambique, Cambodia, Guatemala, Kenya, Morocco, Burkina Faso, and India).

## Discussion

Monitoring progress in the RMNCH coverage index is essential to evaluate whether countries and regions are making progress towards the goal of universal coverage. The present study models the projected coverage and inequalities in the CCI for 70 LMICs up to 2030. Using large-scale datasets, we found that overall RMNCH coverage has improved across all regions, with the exception of southern Europe, from 2000 to 2020 and beyond. Despite this overall progress, there remain substantial gaps in coverage, related to socioeconomic status, education level, and area of residence, both at the national and regional levels. These gaps are projected to persist over the next 8 years and will impede the attainment of the 80% CCI target by 2030. Nevertheless, two countries (Angola and Costa Rica) reached the 80% CCI target in 2000, and 14 additional countries managed to reach it by 2020. Additionally, 18 more countries are projected to reach the target by 2030.

All regions in the current study showed upward trends in coverage, except southern Europe, where coverage declined from 70·1% in 2000 to 60·4% in 2020. During the Millennium Development Goals era, effective RMNCH intervention coverage was scaled up in many LMICs to reduce maternal and child morbidity and mortality.^18^ This global initiative led to rapid progress in several RMNCH care services, such as increasing access to family planning using modern contraceptive methods, increasing the number of births attended by skilled health-care providers, and increasing child vaccinations.^19^ The observed improvements in RMNCH coverage in countries such as Armenia and Jordan in western Asia could be attributed to better access to these services.^9^ Similarly, the significant progress noted in sub-Saharan Africa, where Rwanda's coverage increased substantially from 36·7% in 2000 to 75·6% in 2020, could be caused by strong political commitment, successful policy reforms, reducing out-of-pocket expenditures, and strengthening community-based health-care delivery mechanisms.^20^ However, the downward trend in southern Europe is difficult to explain. The southern European region in this study consists of three western Balkan countries: Albania, Bosnia and Herzegovina, and Montenegro. These countries, which are not EU members, face unique health and socioeconomic challenges. One major challenge is the high emigration rates in the medical sector, which exacerbate labour shortages in their health-care systems.^21^ Consequently, the per-capita availability of medical staff in these countries is lower than in EU and Organisation for Economic Co-operation and Development (OECD) nations. Emigration data highlight a substantial outflow of home-trained doctors and nurses to OECD countries, with Albania having the highest emigration rates in the region for both professions. The number of doctors and nurses from the western Balkans working in OECD countries surpasses the number of home-trained professionals. Although this interpretation should be approached cautiously, there is a possibility that the rising medical brain drain, perhaps linked to EU expansion, has had a role in the decline of RMNCH coverage.

In addition to providing an understanding of where progress has been made in RMNCH coverage, it is equally important to understand the drivers behind regional and national findings, such as differences between urban and rural areas, wealth inequities, and maternal education. This evidence can inform the best targeting of scarce resources to drive maximum change. Regarding urban–rural differences, pronounced rural inequalities exist. As reported in previous literature,^9^, ^22^ rural dwellers face numerous obstacles in accessing health-care services, including a scarcity of qualified health professionals, geographical challenges, adverse weather conditions, and financial hurdles, leading to higher rates of morbidity and mortality in this population.^23^, ^24^ Importantly, the present study showed that the coverage gap between urban and rural areas has decreased since 2000, as some countries have implemented successful strategies to improve RMNCH coverage in both areas. For example, Cambodia has made important efforts to improve the quantity and quality of its health workforce and infrastructure, especially in the public sector,^24^ by ensuring regular presence of health-care providers in both urban and rural centres through work promotion and social benefit schemes.^24^ Additionally, over the past decades, many LMICs have faced substantial migration from rural to urban areas, leading to a decline in urban advantages.^25^ Such within-country exoduses lead to increased urban poverty, which might eventually contribute to reducing wealth-based and area-based coverage inequalities. However, Ethiopia is a counter example of a country where the coverage gap between urban and rural areas has increased. Investments to expand urbanisation between 2010 and 2019 led to higher inequalities in coverage between urban and rural areas. Although urban coverage increased from 43·3% in 2000 to 77·4% in 2020, it increased only from 16·5% to 47·1% in rural areas over the same period.^26^

The pro-rich inequality in coverage is expected to persist over time in most countries. Of the eight countries that had a coverage gap of at least 30 percentage points between the highest-income and lowest-income quintiles in 2000, two-thirds are expected to still have such a disparity by 2030. Heterogeneity in coverage was noticeable at both the national and regional levels, which is in line with previous findings.^4^, ^6^ For example, in Bangladesh, the government is yet to provide health insurance, making health-care access strongly associated with people's incomes.^27^, ^28^ Wealth-based inequalities in coverage can also occur in countries with social health insurance schemes, such as Sudan, because of inadequate investment in the health system and uncontrolled communicable diseases.^29^ The Maldives and Thailand are notable exceptions, because the lowest income quintile had higher RMNCH coverage than the wealthiest quintile. The Maldives has benefited from substantial financial and social improvements over the past decades, allowing them to expand their public health coverage and national insurance scheme, making substantial progress towards UHC.^30^, ^31^ Thailand has a well established public health coverage system thanks to its universal health scheme, which covers 99·5% of the population for essential health services.^32^ Another example worth mentioning is Zimbabwe, which successfully reduced its wealth-based coverage gap while also improving coverage for both the highest-income quintile (the 80% target was achieved in 2020) and the lowest-income quintile (the 80% target is expected to be achieved by 2030) of its population. This success is attributed to substantial improvements in the country's political and economic conditions and support from international development agencies.^33^

Level of maternal education has an important effect on RMNCH coverage. In most countries, the CCI is higher in populations in which women have higher levels of education. This might be caused by the fact that women with lower or no education tend to seek health-care services less frequently than women with higher education.^34^ Ghana has successfully reduced maternal and child mortality by prioritising education and health care through funding of public health services and providing free universal primary and secondary education.^35^ In India, education has been a key determinant in the use of antenatal care and skilled-birth-attendant care services among young mothers.^36^ Educated women were found to have better access to health-care information, a greater awareness of the negative consequences of not receiving maternal care, and made better decisions about health-care service use. These examples suggest that increased access to education, particularly for girls and women, is associated with higher RMNCH coverage.^16^, ^17^

We used a Bayesian hierarchical model to estimate the trends in CCI and develop projections up to 2030 for 70 LMICs at both the regional and national levels. Bayesian models were chosen for their ability to efficiently handle complex data structures, small sample sizes, and model misspecifications. We did subgroup analyses on the basis of socioeconomic status, maternal educational status, and area of residence to assess the magnitude and evolution of coverage inequalities within countries. The use of data from the national DHS and MICS surveys was an important strength of this study, enabling us to do a robust time-trend analysis of key RMNCH indicators and to disaggregate coverage indicators at both the national and regional levels.

This study also had some limitations. First, the CCI was used as a composite measure of RMNCH coverage, which might have limited the identification of differential inequalities in specific indicators, such as family planning, immunisation, or delivery care. However, using several standalone indicators would have complicated cross-country and cross-regional comparative assessments. Second, the association between coverage and wealth index might be biased by omitted variables. For example, higher-income individuals are more likely to live in urban areas, which might have led to an overestimation of the positive association between wealth and coverage. Third, the use of wealth indices has facilitated the monitoring of inequalities and targeted interventions in most countries. Comparing countries on the basis of asset indices is sensible given that these indices provide a measure of relative wealth. For example, it is possible that the lowest-income quintile in a middle-income country possesses greater wealth than the third quintile in a low-income nation. Considering country income as an alternative solution for cross-country analysis is a viable option. It is worth noting that the SDI already accounts for per-capita income of countries. We believe that by making the necessary adjustments to the SDI, we can effectively account for the effect of income on CCI predictions. Fourth, the multicountry data were based on household surveys not necessarily done in every country each year. For estimations and projections of RMNCH service coverage for individual countries, our empirical approach complements missing data for a country with data from neighbouring countries under a missing-at-random assumption. However, data availability might reflect unobserved but systematic heterogeneity across countries. For example, if data missingness is determined by third factors correlating with restricted RMNCH service coverage, our estimations and projections of RMNCH coverage for countries with missing data would be optimistic, leading to a conservative implication for policy actions. Nevertheless, we found that our approach yielded reasonably accurate data imputation through model adequacy checks (appendix pp 12–13), and that overall results were robust to sensitivity testing. Additionally, data regarding ethnicity were not available for analysis. Finally, the quality of health interventions was not considered in the calculation of the CCI, despite being crucial to UHC.

The present analysis sheds light on the remaining challenges in achieving universal coverage of RMNCH interventions. Despite the gradual improvement in access to health-care services in the majority of LMICs, the progress is unequal between countries and regions and falls short of meeting UHC globally. To reach the target of 80% CCI by 2030, many LMICs must step up their efforts in increasing RMNCH coverage, particularly among the lowest-income, rural, and less-educated populations who currently have lower coverage levels. The actions taken by governments must not only be guided by efficiency, but also consider equity considerations to reduce the magnitude of inequality gaps within their population over time.

Further investigation is imperative to uncover the underlying facilitators and obstacles in accessing RMNCH interventions, and the contributing factors to the inequality gaps as identified in this Article. Further research on the quality of health interventions would enhance the evidence. Together, this information is crucial for designing cost-effective, culturally tailored interventions that can enhance overall coverage while concurrently reducing disparities within countries. Our findings on identifying the inequities across LMICs can have an important role in the development of effective strategies, which will not only be crucial for intersectoral programming, policy making, and public health investments, but also in mitigating inequalities that disproportionately affect women and children in underprivileged populations.

## Data sharing

Requests for data sharing will be considered by the corresponding author. All analyses were carried out using publicly available data, and the sources are fully cited in the text. The data related to outcome variable can be accessed at https://www.who.int/data/gho/data/indicators.

## Declaration of interests

We declare no competing interests.
