# Supplementary material for: Reproductive, maternal, newborn, and child health intervention coverage in 70 low-income and middle-income countries, 2000–30: trends, projections, and inequities
Source: Lancet Glob Health. 2023 Sep 4;11(10):e1531–43. doi: 10.1016/S2214-109X(23)00358-3 (PMC10509036; doi:10.1016/S2214-109X(23)00358-3)
Supplement: Supplementary appendix [file mmc1.pdf]

THE LANCET
Global Health

Supplementary appendix

This appendix formed part of the original submission and has been peer reviewed. We post it as supplied by the authors.

Supplement to: Rahman MM, Rouyard T, Khan ST, et al. Reproductive, maternal, newborn, and child health intervention coverage in 70 low-income and middle-income countries, 2000–30: trends, projections, and inequities. *Lancet Glob Health* 2023; published online Sept 4. https://doi.org/10.1016/S2214-109X(23)00358-3.

Supplemental appendix

Reproductive, maternal, newborn and child health intervention coverage in 70 LMICs, 2000-2030: Trends, Projections and Inequities

Table S1: PubMed search January 25, 2023

#	Query	Results	Time
**4**	#1 AND #2 AND #3	254	20:47:59
**3**	"Composite coverage index"[tw] OR "CCI"[tw] OR "coverage index"[tw]	9,194	20:47:43
**2**	"Maternal and child health services"[tw] OR "maternal & child health services"[tw] OR "Maternal and child health service"[tw] OR "Maternal-Child Health Services"[tw] OR "Maternal-Child Health Service"[tw] OR "Maternal Health Services"[tw] OR Maternal Health Service[MeSH] OR "Maternal Health Service"[tw] OR "Maternal Health"[MeSH] OR "Maternal Health"[tw] OR "Child Health Services" [tw] OR "Child Health Service"[tw] OR "Health Services" [MeSH] OR "Health Services"[tw] OR "health service"[tw] OR "Maternal-Child Health"[tw] OR "Women's Health Service"[tw] OR "Women's Health Services"[tw] OR "maternal and neonatal health"[tw] OR "health care"[tw] OR "Maternal health care services"[tw] OR "Maternal and newborn health*" OR "maternal and child health*" OR "reproductive health service*" OR "reproductive health"[tw]	3,118,747	20:47:00
**1**	mother[MeSH] OR mother[tw] OR maternal [MeSH] OR maternal[tw] OR women[tw] OR children[tw] OR pediatric* OR paediatric* OR newborn[MeSH] OR newborn[tw] OR child[tw]	4,725,628	20:46:50

Table S2: Research in context literature

Authors	Title	Journal	Volume	Issue	Pages
Akseer et al. (2016)	Coverage and inequalities in maternal and child health interventions in Afghanistan	BMC Public Health	16	Suppl 2	119-137
Akseer et al. (2019)	Association of Exposure to Civil Conflict with Maternal Resilience and Maternal and Child Health and Health System Performance in Afghanistan	JAMA Network Open	2	11	e1914819
Barros et al. (2020)	Are the poorest poor being left behind? Estimating global inequalities in reproductive, maternal, newborn and child health	BMJ Global Health	5	1	e002229
Baye et al. (2020)	Socio-Economic Inequalities in Child Stunting Reduction in Sub-Saharan Africa	Nutrients	12	1	253
Boerma et al. (2008)	Mind the gap: equity and trends in coverage of maternal, newborn, and child health services in 54 Countdown countries	The Lancet	371	9620	1259-67
Bouilly et al. (2020)	Maternal and child health inequalities among migrants: the case of Haiti and the Dominican Republic	Revista Panamericana de Salud Pública	44		e144
Colomé-Hidalgo et al. (2021)	Exploring wealth-related inequalities in maternal and child health coverage in Latin America and the Caribbean	BMC Public Health	21		1-7
Corsi and Subramanian (2014)	Association between coverage of maternal and child health interventions, and under-5 mortality: a repeated cross-sectional analysis of 35 sub-Saharan African countries	Global Health Action	7	1	24765
Faye et al. (2020)	Large and persistent subnational inequalities in reproductive, maternal, newborn and child health intervention coverage in sub-Saharan Africa	BMJ Global Health	5	1	e002232
Ferreira et al. (2022)	Geographic inequalities in health intervention coverage - mapping the composite coverage index in Peru using geospatial modelling	BMC Public Health	22	1	1-9
Franca et al. (2016)	Coverage and equity in reproductive and maternal health interventions in Brazil: impressive progress following the implementation of the Unified Health System	International Journal for Equity in Health	15		1-2
Gandhi et al. (2021)	Level of inequality and the role of governance indicators in the coverage of reproductive maternal and child healthcare services: Findings from India	Plos One	16	11	e0258244
Garchitorena et al. (2018)	Early changes in intervention coverage and mortality rates following the implementation of an integrated health system intervention in Madagascar	BMJ Global Health	3	3	e000762
Gebremedhin et al. (2023)	Determinants of continuum of care for maternal, newborn, and child health services in Ethiopia: Analysis of the modified composite coverage index using a quantile regression approach	Plos One	18	1	e0280629
Hasan et al. (2020)	Meeting the Global Target in Reproductive, Maternal, Newborn, and Child Health Care Services in Low- and Middle-Income Countries	Global Health: Science and Practice	8	4	654-655
Kachoria et al. (2022)	The association of religion with maternal and child health outcomes in South Asian countries	Plos One	17	7	e0271165

Modi et al. (2019)	mHealth intervention "ImTeCHO" to improve delivery of maternal, neonatal, and child care services-A cluster-randomized trial in tribal areas of Gujarat, India	Plos Medicine	16	10	e1002939
Ngyuen et al. (2021)	Trends in, projections of, and inequalities in reproductive, maternal, newborn and child health service coverage in Vietnam 2000-2030: A Bayesian analysis at national and sub-national levels	The Lancet Regional Health-Western Pacific	15		100230
Oh et al. (2020)	Factors associated with the continuum of care for maternal, newborn and child health in The Gambia: a cross-sectional study using Demographic and Health Survey 2013	BMJ Open	10	11	e036516
Pandey et al. (2021)	Progress in Reducing Inequalities in Reproductive, Maternal, Newborn and Child Health Services in Nepal	Journal of Nepal Health Research Council	19	1	140-147
Parvin et al. (2022)	Socioeconomic inequalities in the continuum of care across women's reproductive life cycle in Bangladesh	Scientific Reports	12	1	15618
Restrepo-Méndez et al. (2015)	Progress in reducing inequalities in reproductive, maternal, newborn,' and child health in Latin America and the Caribbean: an unfinished agenda	Revista Panamericana de Salud Pública	38	1	9-16
Sakuma et al. (2019)	Determinants of continuum of care for maternal, newborn, and child health services in rural Khammouane, Lao PDR	Plos One	14	4	e0215635
Singh et al. (2013)	Equity in maternal, newborn, and child health care coverage in India	Global Health Action	6	1	22217
Thapa et al. (2020)	Equity and Coverage in the Continuum of Reproductive, Maternal, Newborn and Child Health Services in Nepal-Projecting the Estimates on Death Averted Using the LiST Tool	Maternal and Child Health Journal	24		22-30
Victora et al. (2012)	How changes in coverage affect equity in maternal and child health interventions in 35 Countdown to 2015 countries: an analysis of national surveys	The Lancet	380	9848	1149-1156
Victora et al. (2017)	The contribution of poor and rural populations to national trends in reproductive, maternal, newborn, and child health coverage: analyses of cross-sectional surveys from 64 countries	The Lancet Global Health	5	4	e402-7
Wehrmeister et al. (2016)	Summary indices for monitoring universal coverage in maternal and child health care	Bulletin of the World Health Organization	94	12	903
Wehrmeister et al. (2020)	Measuring universal health coverage in reproductive, maternal, newborn and child health: An update of the composite coverage index	Plos One	15	4	e0232350
Wehrmeister et al (2020)	Wealth-related inequalities in the coverage of reproductive, maternal, newborn and child health interventions in 36 countries in the African Region	Bulletin of the World Health Organization	98	6	394

Table S3: Country specific data Sources

Country	Survey name and year
Afghanistan	DHS 2015
Albania	DHS 2008, 2017
Algeria	MICS 2012, 2018
Angola	DHS 2015
Armenia	DHS 2000, 2005, 2010, 2015
Azerbaijan	DHS 2006
Bangladesh	DHS 1999, 2004, 2007, 2011, 2014, 2017
Belize	MICS 2011, 2015
Benin	DHS 2001, 2006, 2011, 2017; MICS 2014
Bolivia	DHS 1998, 2003, 2008
Bosnia and Herzegovina	MICS 2011
Burundi	DHS 2010, 2016
Burkina Faso	DHS 1998, 2003, 2010
Cambodia	DHS 2000, 2005, 2010, 2014
Cameroon	DHS 1998, 2004, 2011, 2018; MICS 2014
Central African Republic	MICS 2010, 2018
Chad	DHS 2004, 2014; MICS 2010
Colombia	DHS 2000, 2005, 2010
Comoros	DHS 2012
Congo	DHS 2005, 2011; MICS 2014
Cote d'Ivoire	DHS 1998, 2011; MICS 2016
Costa Rica	MICS 2011, 2018
Democratic Republic of the Congo	DHS 2007, 2013; MICS 2010, 2017
Dominican Republic	DHS 1999, 2002, 2007, 2013; MICS 2014, 2019
Egypt	DHS 2000, 2005, 2008, 2014
El Salvador	MICS 2014
Eswatini	DHS 2006; MICS 2010, 2014
Ethiopia	DHS 2000, 2005, 2011, 2016
Gabon	DHS 2000, 2012
Gambia	DHS 2013, 2019; MICS 2010, 2018
Ghana	DHS 1998, 2003, 2008, 2014; MICS 2011, 2017
Guatemala	DHS 1998, 2014
Guinea	DHS 1999, 2005, 2012, 2018
Guinea-Bissau	MICS 2014, 2018
Guyana	DHS 2009; MICS 2014, 2019
Haiti	DHS 2000, 2005, 2012, 2016
Honduras	DHS 2005, 2011; MICS 2019
India	DHS 1998, 2005, 2015
Indonesia	DHS 2002, 2007, 2012, 2017
Iraq	MICS 2006, 2011, 2018
Jordan	DHS 2002, 2007, 2012, 2017
Kenya	DHS 1998, 2003, 2008, 2014
Kazakhstan	DHS 1999, MICS 2010
Kiribati	MICS 2018
Kyrgyzstan	DHS 2012; MICS 2014, 2018
Laos	MICS 2011, 2017
Lesotho	DHS 2004, 2009, 2014; MICS 2018
Liberia	DHS 2007, 2013, 2019
Madagascar	DHS 2003, 2008, MICS 2018
Malawi	DHS 2000, 2004, 2010, 2015; MICS 2013, 2019
Maldives	DHS 2009, 2016
Mali	DHS 2001, 2006, 2012, 2018; MICS 2009, 2015
Mauritania	MICS 2011, 2015
Mexico	MICS 2015
Mongolia	MICS 2010, 2013, 2018
Montenegro	MICS 2013

Morocco	DHS 2003
Mozambique	DHS 2003, 2011, 2015
Myanmar	DHS 2015
Namibia	DHS 2000, 2006, 2013
Nepal	DHS 2001, 2006, 2011, 2016; MICS 2010, 2014, 2019
Nicaragua	DHS 2001
Niger	DHS 1998, 2006, 2012
Nigeria	DHS 1999, 2003, 2008, 2013, 2018; MICS 2007, 2011, 2016
Pakistan	DHS 2006, 2012, 2017
Panama	MICS 2013
Papua New	DHS 2016
Paraguay	MICS 2016
Peru	DHS 2000, 2004, 2005, 2006, 2007, 2008, 2009, 2010, 2011, 2012, 2013, 2014, 2015, 2016, 2017, 2018, 2019, 2020
Philippines	DHS 1998, 2003, 2008, 2013, 2017
Republic of Moldova	DHS 2005; MICS 2012
Rwanda	DHS 2000, 2005, 2010, 2014, 2019
Samoa	MICS 2019
Sao Tome and Principe	DHS 2008; MICS 2014, 2019
Senegal	DHS 2005, 2010,2012, 2014, 2015, 2016, 2017, 2018, 2019
Sierra Leone	DHS 2008, 2013,2019; MICS 2010, 2017
South Africa	DHS 1998, 2016
Sudan	MICS 2010, 2014
Tajikistan	DHS 2012, 2017
Thailand	MICS 2012, 2015
The former Yugoslav Republic of Macedonia	MICS 2011
Timor-Leste	DHS 2009, 2016
Togo	DHS 1998, 2013; MICS 2010, 2017
Tonga	MICS 2019
Tunisia	MICS 2011, 2018
Turkmenistan	MICS 2015
Tuvalu	MICS 2019
Uganda	DHS 2000, 2006, 2011, 2016
Ukraine	MICS 2012
United Republic of Tanzania	DHS 2004, 2010, 2015
Viet Nam	DHS 2002, MICS 2010, 2013
Yemen	DHS 2013
Zambia	DHS 2001, 2007, 2013, 2018
Zimbabwe	DHS 2005, 2010, 2015; MICS 2014

Note. DHS: Demographic and Health Surveys, MICS: Multiple Indicator Cluster Survey. Survey year 1998 and 1999 were assumed as survey year 2000. Red color indicate single data point.

Appendix e-method 1: Coverage Composite Index (CCI) computation

CCI is the weighted average of percentage coverage of the eight interventions along four stages of continuum of care: reproductive care; maternal care; childhood immunization; and management of childhood illness. For the current study, data are derived from re-analysis of Demographic and Health Surveys (DHS) and Multiple Indicator Cluster Surveys (MICS) micro-data using the standard indicator definitions as published in DHS and MICS. Information was obtained and on women aged 15–49 years and children aged less than 5 years. The index is calculated as below:

$$CCI = 1/4(DFPSm + \frac{ANC4 + SBA}{2} + \frac{BCG + 2 \times DPT3 + MSL}{4} + \frac{ORS + CAREP}{2})$$

where DFPSm is demand for family planning satisfied with modern methods; ANC4 is 4+ antenatal care visits with any provider; SBA is skilled birth attendant; BCG is bacille Calmette–Guérin vaccine, DPT3 is three doses of Diphtheria, Pertussis, and Tetanus vaccine; MSL is measles immunization; ORS is oral rehydration salts; and CAREP is care seeking for suspected pneumonia. The indicators had equal weights, except for DPT3, which received a weight of two because it requires more than one dose or more than one contact with the provider.

1) D**FPSm:** It is defined as the percentage of women of reproductive age (15-49 years) whose demand for family planning is satisfied with modern methods. These include oral contraceptive pill, condoms (male and female), intrauterine devices, sterilization (male and female), injectables, implants (e.g., Norplant), diaphragm, spermicidal agents (foam or jelly), patches, vaginal ring, lactational amenorrhea method (LAM) and emergency contraception (the day-after pill). The numerator is the percentage of women of reproductive age (15-49 years old) who are currently using, or whose partner is currently using, at least one modern contraceptive method. The denominator is the total demand for family planning [the sum of contraceptive prevalence (any method) and the unmet need for family planning].

2) **SBA:** It is defined as the proportion of births attended by skilled health personnel (generally doctors, nurses or midwives but can refer to other health professionals providing childbirth care). It is calculated as the number of births attended by skilled health personnel (doctor, nurse and/or midwife) expressed as total number of live births in the same period [(Number of births attended by skilled health personnel / Total number of live births) x 100]. In the DHS and MICS surveys, the respondent is asked about each live birth and who had helped them during childbirth for a period up to five years (or three years) before the interview.

3) **ANC4:** It is defined as the percentage of women aged 15-49 with a live birth in a given time period that received antenatal care four or more times. It is calculated as (Number of women aged 15-49 attended at least four times during pregnancy by any provider for reasons related to the pregnancy / Total number of women aged 15-49 with a live birth in the same period) x 100. The indicator is based on a standard question that asks if and how many times the health of the woman was checked during pregnancy, and due to data limitations, it is not possible to determine the type of provider for each visit.

4) **DPT3:** It is defined as the percentage of one-year-olds (aged between 12-23 months) who received three doses of the combined diphtheria, pertussis and tetanus toxoid vaccine in a given year. The indicator is derived by dividing the total number of vaccinations given by the number of children in the target population and the vaccine time is estimated before the DHS and MICS surveys.

5) **MSL:** It is defined as the percentage of children under one year of age who have received at least one dose of measles-containing vaccine in a given year. The indicator is derived by dividing the total number of vaccinations given by the number of children in the target population and the vaccine time is estimated either any time before the survey or before the age of 12 months.

6) **BCG:** It is defined as the percentage of one-year-olds who have received one dose of Bacillus Calmette-Guérin (BCG) vaccine in a given year. The indicator is derived by dividing the total number of vaccinations given by the number of children in the target population and the vaccine time is estimated before the DHS and MICS surveys.

7) **ORS:** It is defined as the percentage of children under 5 years of age with diarrhoea in the two weeks preceding the survey receiving ORs (fluids made from ORS packets or pre-packaged ORS fluids). Mothers or caregivers of children under five years of age are asked if the child had diarrhoea at any time in the past 2 weeks, and if so, whether or not ORs was given. A limitation of this measurement approach is that the severity of diarrhoea is not ascertained.

8) **CAREP:** It is defined as the percentage of children under 5 years of age with symptoms of pneumonia [cough and difficult breathing (not due to a problem in the chest and a blocked nose)] in the two weeks preceding the survey taken to an appropriate health facility or provider. The definition is based on the mother's perceptions of a child who has a cough; is breathing faster than usual with short, quick breaths; or is having difficulty breathing, excluding children who had only a blocked nose. Mothers or caregivers of children under five years are asked if the child had symptoms of acute respiratory infection (ARI), and if so, whether treatment was sought and where it was sought.

References:

• WHO. Data. GHO. Indicators. Available at: https://www.who.int/data/gho/data/indicators (Accessed on 05.11.22).
• Boerma JT, Bryce J, Kinfu Y, Axelson H, Victora CG. Mind the gap: equity and trends in coverage of maternal, newborn, and child health services in 54 Countdown countries. Lancet (London, England). 2008 Apr 1;371(9620):1259-67.
• Boerma T, AbouZahr C, Evans D, Evans T. Monitoring intervention coverage in the context of universal health coverage. PLoS medicine. 2014 Sep 22;11(9):e1001728.
• Wehrmeister FC, Restrepo-Mendez MC, Franca GV, Victora CG, Barros AJ. Summary indices for monitoring universal coverage in maternal and child health care. Bulletin of the World Health Organization. 2016 Dec 12;94(12):903.
• Barros AJ, Victora CG. Measuring coverage in MNCH: determining and interpreting inequalities in coverage of maternal, newborn, and child health interventions. PLoS Med. 2013;10(5):e1001390.

Appendix e-method 2: predicator variables

Socio-demographic index (SDI):

A socio-demographic index (SDI) is a measure of socioeconomic development that can be used to compare countries at a global level. It is an important indicator of a country's health outcomes[1] and is calculated by taking into account a variety of factors, such as per capita income, total fertility rate, and educational attainment. [2] SDI is calculated using lag-distributed per capita income, total fertility rate, and educational attainment data. Countries with a SDI value between 0 and 1 typically display lower educational attainment, lower per capita income, and higher total fertility rates than those near 1. Conversely, countries with a SDI value close to 1 tend to have higher incomes and educational attainment, as well as lower fertility rates. The Global Burden of Disease Study has provided SDI values for countries from 2000-2030, providing a useful snapshot of socioeconomic development during this period.[2] The SDI data can be found at http://www.healthdata.org/health-financing/.

Per capita total health expenditure:

Data on per capita total health expenditures in constant 2018 US dollars for the period 2000-2016 and forecasted per capita total health expenditures for the period 2017-2030 were obtained from studies conducted by the Institute for Health Metrics and Evaluation (IHME), which gathered data from studies conducted by the Institute. IHME forecasted total health expenditures per capita using health expenditure estimates for 1995-2016, taking into account factors such as total fertility rate, proportion of elderly population (65 years and older), per capita GDP, and total government spending. Health expenditures for 2000-2016 have been compared with projected values for 2017-2030 to determine total health expenditures per capita. GDP spent on health data can be found at http://www.healthdata.org/health-financing/.

GDP per capita:

Gross domestic product (GDP) per capita is an important economic measure used by the International Monetary Fund (IMF) to compare the economic output of countries worldwide. GDP per capita is a measure of the total economic output of a country divided by its population. GDP per capita data was obtained from the IMF for the years 2000-2027, and then for 2028 to 2030 we assumed the same GDP per capita as in 2027. This GDP data comes from the IMF's website: https://www.imf.org/en/Publications/WEO/.

Wealth index

As a proxy measure of socioeconomic status, the wealth index is widely used in low and middle income countries as an asset-based measure of household wealth. [3] Wealth index information is usually included in nationally representative surveys such as the DHS and MICS. An index of household wealth is typically calculated based on several factors, such as the availability of water and sanitation facilities. An estimation of a wealth score is then performed using principal component analysis (PCA), which represents a household's position relative to the rest of the households in a country. [4,5]To determine a relative measure of household wealth, all households in a survey are divided into quintiles, and the lowest 20% values represent the poorest 20% of households (i.e., the poorest 20% of households), and the highest 20% values represent the wealthiest households.

Appendix e-method 3: Bayesian model

Bayesian approach was used and favoured with the aim to project probabilities, which would not be possible using a frequentist approach. The essential difference between the two approaches is how probability is used. If we were to use a frequentist approach, the 95% CIs cannot calculate the probability of observing a future value, and hence we would be limited to using probability to *only* model certain processes (using process of "sampling"). For the purpose of our study, we were required to use probability more widely to model both sampling and other kinds of uncertainty which could only be conducted using Bayesian approach. As a result of the advantage to produce probabilistic-oriented inferences, Bayesian methods are increasingly being applied to scientific fields, particularly in ecology where many cases outperform deterministic approaches. Since ecological modelling is characterized by high uncertainty due to the complex and often unknown cause-effect relationships among variables, a probabilistic approach is necessary to yield distributions of possible outcomes - in essence, transforming uncertainty into probability thresholds.

This unique advantage of Bayesian approach over frequentist approach was key to conducting our study. Another advantage of using Bayesian methods in our study was the ability to combine prior knowledge about parameters with evidence from data. This method is favored for analysis of hierarchical models such as our study, which enables: flexibility in specifying hierarchical structures of parameters using priors; ability to handle small samples and model misspecification (overparameterization of the likelihood can be resolved with well-chosen priors); explicit handling of uncertainty; and intuitive and easy interpretation of results (credible interval versus confidence interval). In frequentist approach, confidence interval (CI) talks about central area that contains 95% of distribution. Clearly, 95% **CI** means that with a large number of repeated samples, 95% of such calculated CIs would include the true value of the parameter. In the Bayesian approach, credible interval (CrI) provides an interval in which there is a 95% change of parameter lying, we can drive this directly from our posterior distribution. The 95% CI included the fixed parameter 95% of the trials under the null model, whereas the 95% CrI contains the parameter with a probability of 0.95.

The following Bayesian hierarchical model was used to estimate the trends in, and projections of CCI up to 2030, at country and residence level:

$$y_{ijk} \sim N(X\beta_{ijk}, \tau^2)$$

$$y_{ijk} = \alpha_{ijk} + \beta_{1,ijk}year_{ijk} + \beta_{2,ijk}residence_{ijk} + \beta_{3,jk}SDI_{ijk} + \beta_{4,jk}GGDPH_{ijk} + \beta_{5,jk}GDPC_{ijk} + \varepsilon_{ijk}$$

where y_{ijk} is the logit-transformed probability corresponding to the CCI in the *i*th year for the *j*th country in the *k*th region, and ε_{ijk}quantifies random variations in CCI that were not explained by covariates. The random intercept α_{ijk} was modeled hierarchically by country j and region k:

$$\alpha_{ijk} \sim N(\mu_{ij}, \sigma_{ij}^2)$$

$$\mu_{ij} \sim N(\mu_i, \sigma_i^2)$$

$$\mu_i \sim N(\mu, \sigma^2)$$

The model incorporated covariates that aid in predicting CCI, including year, residence area, and time-varying country-level factors such as SDI, GGDPH, and GDPC. Note that $residence_{ijk} = \{National_{ijk}, Urban_{ijk}, Rural_{ijk}\}$ represents national, urban, and rural levels and is encoded as a dummy variable. After estimating the regression parameters, we can predict the CCI at the national, urban, and rural levels. The term α_{ijk} gives the random intercept. The error term ε_{ijk} quantifies random variations in CCI that are not explained by the covariates. We also incorporated interactions of residence with country to account for potential changes in associations over time, particularly in relation to the availability of treatments for maternal and child health.

The slope β_{ijk} was modeled hierarchically by year *i*, country *j* and region *k*:

$$\beta_{ijk} \sim N(\beta_{ij}, \sigma_{ij}^2)$$

$$\beta_{ij} \sim N(\beta_i, \sigma_i^2)$$

$$\beta_i \sim N(\beta, \sigma^2)$$

Non-informative priors were defined as

$$\beta \sim N(0, 10000)$$

$$\sigma_i, \sigma, \mu, \tau \sim gamma(0.001, 0.001)$$

To explore disparities in CCI based on wealth, the basic model was extended to estimate the quintile-specific CCI using the same country-level covariates. The wealth quintile model incorporates the following variables: year, wealth quintile, SDI, GGDPH, and GDPC. The wealth quintiles are categorised into five groups: lower (Q1), middle (Q3), upper (Q4), and highest (Q5). Note that $wealth_{ijk} = \{Q1_{ijk}, Q2_{ijk}, Q3_{ijk}, Q4_{ijk}, Q5_{ijk}\}$ is represented by dummy variables encoding each wealth quintile from Q1 to Q5. A similar methodology was used to explore disparities in CCI based on maternal education levels. Initially, we calculated the CCI for each country, residence, and year based on the posterior predictive distribution. To derive estimates and projections for country aggregates, such as regions, we calculated weighted averages of the constituent country-level estimates. A random effects meta-analysis was used to obtain an average at the regional and global levels. We used the posterior samples for the aggregate regional level model to calculate the mean and 95% credible intervals (CrIs).

Table. S4: Model selection

Model (variables)	p^D	DIC	Mean deviance (95% CrI)	PPC
Model 1: CCI=year	4.8	1252.49	1247.71 (1243.56-1255.22)	0.498
Model 2: CCI=year+SDI	6.3	889.94	883.62 (878.85-892.17)	0.498
Model 3: CCI=year+SDI+ GGDPH	6.9	743.633	736.72 (731.38-745.73)	0.468
Model 4: CCI=year+SDI+ GDP	6.8	878.42	871.60 (866.41-880.33)	0.507
Model 5: CCI=year+SDI+ GGDPH+GDP	8.2	745.88	737.66 (731.87-747.26)	0.483
Model 6: CCI= year+SDI+ GGDPH+GDP +DAH	8.6	745.63	737.06 (730.62-746.54)	0.496
Model 7: CCI= year+SDI+ GGDPH+GDP +HRH	9.4	743.05	733.70 (727.30-744.17)	0.487
Model 8: CCI= year+SDI+DAH+HRH	7.9	877.56	869.65 (863.97-879.18))	0.502
Model 9: CCI= year+SDI+ GGDPH+DAH+HRH	10.0	742.96	732.94 (726.40-743.01)	0.504
Model 10: CCI= year+SDI+ GGDPH+GDP+ DAH+HRH	10.3	744.01	733.68 (726.78-733.10)	0.505

DIC, deviance information criterion; PPC, Posterior predictive check plot

SDI, SDI, sociodemographic index, DAH, development assistance for health; HRH, human resource for health; GGDPH, government gross domestic product spending on health; and GDPC, and GDP per capita;

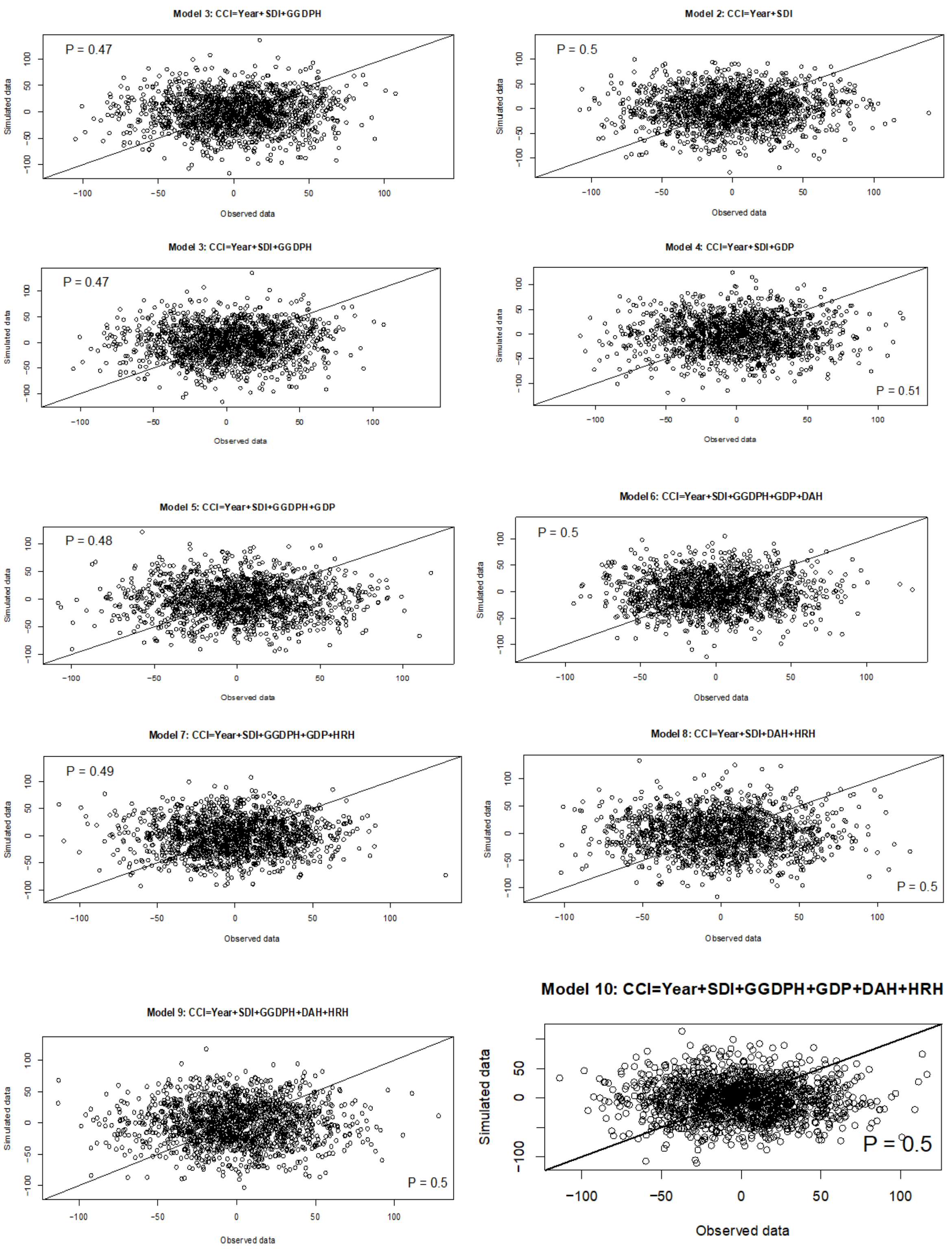

Figure S1: Posterior predictive check plot

Table S5: Posterior mean difference by altering prior distribution on hyperparameters

Country	Posterior mean differences by changing priors (vague vs weekly)						
	2000	2005	2010	2015	2020	2025	2030
Afghanistan	0.30	0.70	0.60	0.50	0.60	0.70	0.90
Albania	0.50	0.40	0.20	0.10	0.10	-0.20	-0.20
Angola	-0.60	-1.40	-0.80	-0.10	-0.60	-0.20	0.10
Armenia	-0.10	-0.10	-0.10	0.00	0.00	0.00	0.00
Azerbaijan	4.90	2.60	-3.80	-4.30	-4.30	-5.00	-5.30
Bangladesh	0.10	0.10	0.00	0.00	0.10	0.20	0.30
Benin	-0.30	-0.20	0.00	-0.10	0.10	0.00	-1.10
Bosnia and Herzegovina	-0.70	-0.50	-0.10	0.40	1.00	1.70	2.00
Burkina Faso	0.00	-0.10	-0.10	-0.20	-0.30	-0.30	-0.30
Burundi	-0.40	-0.30	-0.20	-0.10	0.10	0.20	0.30
Cambodia	0.00	0.10	-0.10	-0.10	-0.20	-0.30	-0.20
Cameroon	0.00	0.00	0.00	0.10	0.20	0.00	0.10
Central African Republic	0.10	0.20	0.10	0.10	0.10	0.20	0.20
Chad	0.10	0.00	0.00	0.10	0.50	0.90	1.20
Colombia	-0.10	0.00	0.00	0.00	-0.10	-0.10	-0.10
Comoros	0.60	0.40	0.20	0.20	0.20	0.10	0.10
Costa Rica	-0.50	-0.40	-0.10	0.00	0.10	0.20	0.30
Democratic Republic of the Congo	-0.30	-0.10	0.00	0.00	0.30	0.50	0.80
Dominican Republic	0.10	0.00	0.10	0.00	0.00	0.10	0.10
Egypt	0.00	0.00	0.10	0.00	0.00	0.10	0.10
El Salvador	-2.40	-1.90	-1.10	0.20	0.10	0.90	0.90
Ethiopia	0.10	0.00	0.10	0.20	0.20	0.30	0.20
Gabon	0.10	0.00	0.00	0.50	0.60	1.20	1.60
Ghana	-0.10	-0.20	-0.20	-0.10	0.00	0.10	0.00
Guatemala	-0.20	-0.30	-0.20	0.20	-0.10	0.40	0.40
Guinea	0.00	0.10	0.10	0.10	-0.10	-0.20	-0.20
Guinea-Bissau	0.20	0.20	-0.10	0.00	-0.10	-0.20	-0.20
Haiti	-0.10	-0.10	-0.10	-0.10	-0.20	-0.20	-0.30
Honduras	0.00	-0.10	0.00	0.00	0.00	-0.10	-0.10
India	0.00	0.10	0.00	0.00	0.10	-0.30	-0.20
Indonesia	0.40	0.10	0.10	0.00	0.00	0.20	0.10
Iraq	0.00	-0.10	-0.10	-0.10	0.00	0.00	0.10
Jordan	0.00	-0.10	-0.20	0.20	0.20	0.10	0.00
Kazakhstan	0.20	0.10	0.10	0.00	0.10	0.10	0.10

Kenya	0.10	0.10	0.00	-0.20	-0.20	-0.30	-0.40
Lesotho	-0.10	0.00	0.00	0.00	0.20	0.20	0.30
Madagascar	-0.30	-0.10	0.20	0.20	0.30	0.20	0.20
Malawi	0.00	0.10	0.10	0.00	0.00	0.00	0.00
Maldives	-0.10	-0.10	0.00	0.10	-0.10	0.10	0.00
Mali	0.00	-0.10	0.00	0.00	0.00	0.00	0.00
Mauritania	-0.60	-0.40	-0.10	0.20	0.40	1.30	1.60
Mexico	-0.70	-0.50	-0.40	0.00	-0.10	0.20	0.40
Mongolia	-0.10	-0.20	-0.20	0.00	0.00	0.10	0.10
Montenegro	0.90	0.60	0.00	-0.40	-0.30	-1.50	-1.70
Morocco	0.70	-0.10	-0.90	-1.30	-1.30	-1.40	-1.30
Mozambique	0.20	0.20	0.10	0.30	0.40	0.40	0.50
Myanmar	1.30	1.10	0.80	0.10	-0.40	-0.10	0.00
Namibia	0.10	0.00	0.00	0.10	-0.70	-0.80	-0.90
Nepal	0.00	0.10	0.20	0.10	0.00	-0.10	0.00
Nicaragua	0.00	0.00	0.00	0.00	0.10	0.10	0.10
Niger	-0.10	-0.10	-0.10	-0.20	-0.20	-0.30	-0.30
Nigeria	-0.40	0.00	0.10	0.20	-0.20	-0.30	-0.20
Pakistan	1.40	0.40	0.30	0.00	0.10	-0.40	-0.60
Panama	-0.60	-0.50	-0.20	0.30	0.20	0.80	1.10
Paraguay	-0.30	-0.30	-0.20	-0.10	-0.20	-0.20	-0.20
Peru	-0.20	0.00	0.00	-0.10	0.20	0.20	0.40
Philippines	-0.10	0.00	0.00	0.00	0.10	-0.10	0.00
Rwanda	0.10	0.20	0.20	0.00	0.00	-0.10	-0.10
Senegal	-0.20	-0.10	0.00	0.10	0.00	-0.50	-0.30
Sierra Leone	0.30	0.10	0.00	0.00	0.00	-0.10	-0.10
South Africa	-0.10	0.10	0.10	-0.10	-0.60	-0.70	-0.90
Sudan	0.60	0.30	0.10	0.00	0.10	-0.20	-0.30
Tajikistan	0.10	0.10	0.20	0.20	0.10	0.20	0.20
Thailand	-0.20	-0.20	-0.10	-0.10	0.00	0.20	0.20
Timor-Leste	-0.40	0.00	-0.20	-0.10	-0.20	0.90	1.20
Togo	0.00	-0.10	-0.10	0.10	0.20	0.40	0.50
Uganda	0.20	0.10	0.10	0.10	0.10	0.10	0.00
Yemen	-0.60	-0.60	-0.60	0.40	0.70	1.00	1.20
Zambia	0.10	0.00	-0.20	0.00	0.10	0.10	0.20
Zimbabwe	-0.90	0.00	0.10	0.00	0.30	0.10	0.20
All countries (mean)	0.03	-0.01	-0.09	-0.03	-0.03	0.01	0.04

Table S6: National level estimate of Gelman Rubin Potential scale reduction factors (PSRF)

Country	Potential scale reduction factors (Point estimate; Upper C.I)						
	2000	2005	2010	2015	2020	2025	2030
Afghanistan	(1.021;1.093)	(1.021;1.093)	(1.021;1.093)	(1.021;1.093)	(1.021;1.093)	(1.021;1.093)	(1.021;1.093)
Albania	(1.021;1.093)	(1.021;1.093)	(1.021;1.093)	(1.021;1.093)	(1.021;1.093)	(1.021;1.093)	(1.021;1.093)
Angola	(1.021;1.093)	(1.021;1.093)	(1.021;1.093)	(1.021;1.093)	(1.021;1.093)	(1.021;1.093)	(1.021;1.093)
Armenia	(1.021;1.093)	(1.021;1.093)	(1.021;1.093)	(1.021;1.093)	(1.021;1.093)	(1.021;1.093)	(1.021;1.093)
Azerbaijan	(1.021;1.093)	(1.021;1.093)	(1.021;1.093)	(1.021;1.093)	(1.021;1.093)	(1.021;1.093)	(1.021;1.093)
Bangladesh	(1.021;1.093)	(1.021;1.093)	(1.021;1.093)	(1.021;1.093)	(1.021;1.093)	(1.021;1.093)	(1.021;1.093)
Benin	(1.021;1.093)	(1.021;1.093)	(1.021;1.093)	(1.021;1.093)	(1.021;1.093)	(1.021;1.093)	(1.021;1.093)
Bosnia	(1.021;1.093)	(1.021;1.093)	(1.021;1.093)	(1.021;1.093)	(1.021;1.093)	(1.021;1.093)	(1.021;1.093)
Burkina Faso	(1.021;1.093)	(1.021;1.093)	(1.021;1.093)	(1.021;1.093)	(1.021;1.093)	(1.021;1.093)	(1.021;1.093)
Burundi	(1.021;1.093)	(1.021;1.093)	(1.021;1.093)	(1.021;1.093)	(1.021;1.093)	(1.021;1.093)	(1.021;1.093)
Cambodia	(1.021;1.093)	(1.021;1.093)	(1.021;1.093)	(1.021;1.093)	(1.021;1.093)	(1.021;1.093)	(1.021;1.093)
Cameroon	(1.021;1.093)	(1.021;1.093)	(1.021;1.093)	(1.021;1.093)	(1.021;1.093)	(1.021;1.093)	(1.021;1.093)
Central African Republic	(1.021;1.093)	(1.021;1.093)	(1.021;1.093)	(1.021;1.093)	(1.021;1.093)	(1.021;1.093)	(1.021;1.093)
Chad	(1.021;1.093)	(1.021;1.093)	(1.021;1.093)	(1.021;1.093)	(1.021;1.093)	(1.021;1.093)	(1.021;1.093)
Colombia	(1.021;1.093)	(1.021;1.093)	(1.021;1.093)	(1.021;1.093)	(1.021;1.093)	(1.021;1.093)	(1.021;1.093)
Comoros	(1.021;1.093)	(1.021;1.093)	(1.021;1.093)	(1.021;1.093)	(1.021;1.093)	(1.021;1.093)	(1.021;1.093)
Costa Rica	(1.021;1.093)	(1.021;1.093)	(1.021;1.093)	(1.021;1.093)	(1.021;1.093)	(1.021;1.093)	(1.021;1.093)
DRC	(1.021;1.093)	(1.021;1.093)	(1.021;1.093)	(1.021;1.093)	(1.021;1.093)	(1.021;1.093)	(1.021;1.093)
Dominican Republic	(1.021;1.093)	(1.021;1.093)	(1.021;1.093)	(1.021;1.093)	(1.021;1.093)	(1.021;1.093)	(1.021;1.093)
Egypt	(1.021;1.093)	(1.021;1.093)	(1.021;1.093)	(1.021;1.093)	(1.021;1.093)	(1.021;1.093)	(1.021;1.093)
El Salvador	(1.021;1.093)	(1.021;1.093)	(1.021;1.093)	(1.021;1.093)	(1.021;1.093)	(1.021;1.093)	(1.021;1.093)
Ethiopia	(1.021;1.093)	(1.021;1.093)	(1.021;1.093)	(1.021;1.093)	(1.021;1.093)	(1.021;1.093)	(1.021;1.093)
Gabon	(1.021;1.093)	(1.021;1.093)	(1.021;1.093)	(1.021;1.093)	(1.021;1.093)	(1.021;1.093)	(1.021;1.093)
Ghana	(1.021;1.093)	(1.021;1.093)	(1.021;1.093)	(1.021;1.093)	(1.021;1.093)	(1.021;1.093)	(1.021;1.093)
Guatemala	(1.021;1.093)	(1.021;1.093)	(1.021;1.093)	(1.021;1.093)	(1.021;1.093)	(1.021;1.093)	(1.021;1.093)
Guinea	(1.021;1.093)	(1.021;1.093)	(1.021;1.093)	(1.021;1.093)	(1.021;1.093)	(1.021;1.093)	(1.021;1.093)
Guinea-Bissau	(1.021;1.093)	(1.021;1.093)	(1.021;1.093)	(1.021;1.093)	(1.021;1.093)	(1.021;1.093)	(1.021;1.093)
Haiti	(1.021;1.093)	(1.021;1.093)	(1.021;1.093)	(1.021;1.093)	(1.021;1.093)	(1.021;1.093)	(1.021;1.093)
Honduras	(1.021;1.093)	(1.021;1.093)	(1.021;1.093)	(1.021;1.093)	(1.021;1.093)	(1.021;1.093)	(1.021;1.093)
India	(1.021;1.093)	(1.021;1.093)	(1.021;1.093)	(1.021;1.093)	(1.021;1.093)	(1.021;1.093)	(1.021;1.093)
Indonesia	(1.021;1.093)	(1.021;1.093)	(1.021;1.093)	(1.021;1.093)	(1.021;1.093)	(1.021;1.093)	(1.021;1.093)
Iraq	(1.021;1.093)	(1.021;1.093)	(1.021;1.093)	(1.021;1.093)	(1.021;1.093)	(1.021;1.093)	(1.021;1.093)
Jordan	(1.021;1.093)	(1.021;1.093)	(1.021;1.093)	(1.021;1.093)	(1.021;1.093)	(1.021;1.093)	(1.021;1.093)
Kazakhstan	(1.021;1.093)	(1.021;1.093)	(1.021;1.093)	(1.021;1.093)	(1.021;1.093)	(1.021;1.093)	(1.021;1.093)
Kenya	(1.021;1.093)	(1.021;1.093)	(1.021;1.093)	(1.021;1.093)	(1.021;1.093)	(1.021;1.093)	(1.021;1.093)
Lesotho	(1.021;1.093)	(1.021;1.093)	(1.021;1.093)	(1.021;1.093)	(1.021;1.093)	(1.021;1.093)	(1.021;1.093)

Madagascar	(1.021;1.093)	(1.021;1.093)	(1.021;1.093)	(1.021;1.093)	(1.021;1.093)	(1.021;1.093)	(1.021;1.093)
Malawi	(1.021;1.093)	(1.021;1.093)	(1.021;1.093)	(1.021;1.093)	(1.021;1.093)	(1.021;1.093)	(1.021;1.093)
Maldives	(1.021;1.093)	(1.021;1.093)	(1.021;1.093)	(1.021;1.093)	(1.021;1.093)	(1.021;1.093)	(1.021;1.093)
Mali	(1.021;1.093)	(1.021;1.093)	(1.021;1.093)	(1.021;1.093)	(1.021;1.093)	(1.021;1.093)	(1.021;1.093)
Mauritania	(1.021;1.093)	(1.021;1.093)	(1.021;1.093)	(1.021;1.093)	(1.021;1.093)	(1.021;1.093)	(1.021;1.093)
Mexico	(1.021;1.093)	(1.021;1.093)	(1.021;1.093)	(1.021;1.093)	(1.021;1.093)	(1.021;1.093)	(1.021;1.093)
Mongolia	(1.021;1.093)	(1.021;1.093)	(1.021;1.093)	(1.021;1.093)	(1.021;1.093)	(1.021;1.093)	(1.021;1.093)
Montenegro	(1.021;1.093)	(1.021;1.093)	(1.021;1.093)	(1.021;1.093)	(1.021;1.093)	(1.021;1.093)	(1.021;1.093)
Morocco	(1.021;1.093)	(1.021;1.093)	(1.021;1.093)	(1.021;1.093)	(1.021;1.093)	(1.021;1.093)	(1.021;1.093)
Mozambique	(1.021;1.093)	(1.021;1.093)	(1.021;1.093)	(1.021;1.093)	(1.021;1.093)	(1.021;1.093)	(1.021;1.093)
Myanmar	(1.021;1.093)	(1.021;1.093)	(1.021;1.093)	(1.021;1.093)	(1.021;1.093)	(1.021;1.093)	(1.021;1.093)
Namibia	(1.021;1.093)	(1.021;1.093)	(1.021;1.093)	(1.021;1.093)	(1.021;1.093)	(1.021;1.093)	(1.021;1.093)
Nepal	(1.021;1.093)	(1.021;1.093)	(1.021;1.093)	(1.021;1.093)	(1.021;1.093)	(1.021;1.093)	(1.021;1.093)
Nicaragua	(1.021;1.093)	(1.021;1.093)	(1.021;1.093)	(1.021;1.093)	(1.021;1.093)	(1.021;1.093)	(1.021;1.093)
Niger	(1.021;1.093)	(1.021;1.093)	(1.021;1.093)	(1.021;1.093)	(1.021;1.093)	(1.021;1.093)	(1.021;1.093)
Nigeria	(1.021;1.093)	(1.021;1.093)	(1.021;1.093)	(1.021;1.093)	(1.021;1.093)	(1.021;1.093)	(1.021;1.093)
Pakistan	(1.021;1.093)	(1.021;1.093)	(1.021;1.093)	(1.021;1.093)	(1.021;1.093)	(1.021;1.093)	(1.021;1.093)
Panama	(1.021;1.093)	(1.021;1.093)	(1.021;1.093)	(1.021;1.093)	(1.021;1.093)	(1.021;1.093)	(1.021;1.093)
Paraguay	(1.021;1.093)	(1.021;1.093)	(1.021;1.093)	(1.021;1.093)	(1.021;1.093)	(1.021;1.093)	(1.021;1.093)
Peru	(1.021;1.093)	(1.021;1.093)	(1.021;1.093)	(1.021;1.093)	(1.021;1.093)	(1.021;1.093)	(1.021;1.093)
Philippines	(1.021;1.093)	(1.021;1.093)	(1.021;1.093)	(1.021;1.093)	(1.021;1.093)	(1.021;1.093)	(1.021;1.093)
Rwanda	(1.021;1.093)	(1.021;1.093)	(1.021;1.093)	(1.021;1.093)	(1.021;1.093)	(1.021;1.093)	(1.021;1.093)
Senegal	(1.021;1.093)	(1.021;1.093)	(1.021;1.093)	(1.021;1.093)	(1.021;1.093)	(1.021;1.093)	(1.021;1.093)
Sierra Leone	(1.021;1.093)	(1.021;1.093)	(1.021;1.093)	(1.021;1.093)	(1.021;1.093)	(1.021;1.093)	(1.021;1.093)
South Africa	(1.021;1.093)	(1.021;1.093)	(1.021;1.093)	(1.021;1.093)	(1.021;1.093)	(1.021;1.093)	(1.021;1.093)
Sudan	(1.021;1.093)	(1.021;1.093)	(1.021;1.093)	(1.021;1.093)	(1.021;1.093)	(1.021;1.093)	(1.021;1.093)
Tajikistan	(1.021;1.093)	(1.021;1.093)	(1.021;1.093)	(1.021;1.093)	(1.021;1.093)	(1.021;1.093)	(1.021;1.093)
Thailand	(1.021;1.093)	(1.021;1.093)	(1.021;1.093)	(1.021;1.093)	(1.021;1.093)	(1.021;1.093)	(1.021;1.093)
Timor-Leste	(1.021;1.093)	(1.021;1.093)	(1.021;1.093)	(1.021;1.093)	(1.021;1.093)	(1.021;1.093)	(1.021;1.093)
Togo	(1.021;1.093)	(1.021;1.093)	(1.021;1.093)	(1.021;1.093)	(1.021;1.093)	(1.021;1.093)	(1.021;1.093)
Uganda	(1.021;1.093)	(1.021;1.093)	(1.021;1.093)	(1.021;1.093)	(1.021;1.093)	(1.021;1.093)	(1.021;1.093)
Yemen	(1.021;1.093)	(1.021;1.093)	(1.021;1.093)	(1.021;1.093)	(1.021;1.093)	(1.021;1.093)	(1.021;1.093)
Zambia	(1.021;1.093)	(1.021;1.093)	(1.021;1.093)	(1.021;1.093)	(1.021;1.093)	(1.021;1.093)	(1.021;1.093)
Zimbabwe	(1.021;1.093)	(1.021;1.093)	(1.021;1.093)	(1.021;1.093)	(1.021;1.093)	(1.021;1.093)	(1.021;1.093)

Table S7: Composite coverage index at the national and regional level, 2000-2030

**country**	**Proportion, (95% CrI)**				Proby* achive 80% target in 2030, %
	**2000**	**2010**	**2020**	**2030**	
**Overall (70 countries)**	**54.1 (53.3-54.9)**	**62.3 (61.8-62.7)**	**69.8 (69.4-70.2)**	**76.4 (75.8-76.9)**	
**Southern Asia**	**51.8 (50.9-52.6)**	**62.1 (61.6-62.5)**	**71.4 (70.9-71.8)**	**79.2 (78.6-79.7)**	
Afghanistan	30.7 (15.5-49.6)	44.6 (36.2-53.3)	57.1 (48.1-65.6)	68.5 (48.3-84.6)	8.7%
Bangladesh	53 (49.4-56.7)	63.8 (61.1-66.5)	73.3 (70.1-76.3)	81.3 (75.7-85.7)	80.8%
India	55.7 (51.3-59.9)	66.9 (63.1-70.2)	76.8 (72.4-80.7)	85.3 (79.8-89.7)	98.6%
Maldives	69.3 (58.9-78.5)	71.8 (67.5-75.5)	76.8 (69.9-82.9)	72.4 (61.2-81.6)	6.6%
Nepal	51.2 (47.4-55.1)	62.6 (59.9-65.2)	73.2 (70-76.3)	81.3 (77.9-84.7)	76.2%
Pakistan	45.9 (35.3-57)	55.2 (51.1-59.3)	65.5 (60.1-70.3)	75.6 (68.3-82)	13.3%
**South-eastern Asia**	**58.8 (58-59.5)**	**69 (68.6-69.4)**	**77.7 (77.3-78)**	**84.4 (84-84.8)**	
Cambodia	40.3 (36.1-44.4)	62.3 (58.8-65.4)	80.5 (76.8-83.9)	91 (87.8-93.6)	100%
Indonesia	67 (62.6-71.1)	72.6 (69.4-75.6)	77.8 (74.4-80.9)	81.8 (76.4-86.5)	76.7%
Myanmar	57.5 (30-80.3)	62.8 (42.6-80.2)	75.3 (68.3-81.8)	81 (66.6-91.3)	59.5%
Philippines	59.2 (55.4-62.7)	66.5 (63.5-69.3)	73.3 (69.7-76.8)	79 (73.6-83.6)	35.7%
Thailand	79.9 (67.8-89)	86.2 (83.5-88.8)	89.1 (85.6-92)	91.7 (84.3-96.4)	99.1%
Timor-Leste	50 (35.2-64.6)	59 (54.1-63.7)	68.9 (61.9-75.3)	73.7 (58.8-85.5)	14.3%
**Central Asia**	**70.3 (69.7-71)**	**77 (76.7-77.4)**	**82.6 (82.3-82.9)**	**87 (86.7-87.4)**	
Kazakhstan	70.3 (65.4-74.6)	85.5 (82.8-88)	91.5 (88.3-93.7)	95.1 (91.9-97.1)	100%
Tajikistan	63.1 (48.2-75.2)	68.5 (62.2-73.9)	73.3 (68.2-77.8)	77 (66.4-85.1)	28%
**Eastern Asia**	**76.8 (69-83.4)**	**78.3 (75.1-81.2)**	**79.8 (76.1-83.1)**	**82.1 (74.1-88.4)**	
Mongolia	76.8 (69-83.4)	78.3 (75.1-81.2)	79.8 (76.1-83.1)	82.1 (74.1-88.4)	78.7%
**Western Asia**	**56.5 (55.7-57.4)**	**62 (61.6-62.5)**	**67.2 (66.8-67.6)**	**72.1 (71.4-72.7)**	

Armenia	53.8 (49.5-57.7)	67.5 (64.4-70.3)	78.5 (74.8-81.5)	86.5 (82.5-89.9)	99.6%
Azerbaijan	78.3 (32.4-97.4)	19.1 (2.5-60.4)	27.8 (3.6-75.1)	25.5 (0.9-84.9)	1.7%
Iraq	67.5 (53.7-79.5)	64.1 (60-68.1)	67.7 (59.8-75.2)	70.5 (60.1-80)	2.9%
Jordan	66.9 (61.5-71.8)	74.2 (71-77.4)	75.3 (71.4-79.1)	80.5 (74.6-85.2)	60.4%
Yemen	37.6 (20.5-57.7)	48.4 (41.3-55.4)	41.4 (23.9-60.3)	51.7 (25-77.8)	0.6%
**Sub-Saharan Africa**	**46.3 (45.5-47.1)**	**55.5 (55-55.9)**	**64.2 (63.8-64.7)**	**72.2 (71.5-72.8)**	
Angola	89.3 (63.2-98.9)	49.7 (39-59.6)	81.4 (66.9-92.3)	85.6 (70.3-95.5)	80.8%
Benin	47.2 (43.2-51.2)	49.1 (46.1-51.8)	52.9 (48.7-56.9)	62.8 (51.8-72)	0%
Burkina Faso	40 (35.8-44.1)	58.3 (53.7-62.8)	74.8 (67.5-81.6)	85.5 (78.3-91.7)	92.5%
Burundi	51 (40.8-60.9)	59.2 (54.7-64)	66.3 (59.9-71.8)	72.6 (61.5-81.5)	8.5%
Cameroon	44.3 (40.6-47.9)	48.3 (45.2-51.5)	51.8 (46.9-56.1)	58.2 (52.1-63.9)	0%
Central African Republic	34.9 (26.3-44.9)	37.8 (33.1-43.1)	39.7 (34.3-45.4)	45 (33.5-56.4)	0%
Chad	18.5 (14.6-22.7)	26.8 (23.3-30.5)	39.7 (32.2-47)	54.1 (40.3-66.9)	0%
Comoros	43.1 (28.3-58.7)	51.1 (44.5-57)	60.1 (48-70.7)	67.9 (45.5-84.7)	8.6%
Democratic Republic of the Congo	42.7 (35.9-49)	45 (41.5-48.5)	46.7 (41.3-52.5)	47.5 (36.9-58.7)	0%
Ethiopia	29.9 (26.4-33.7)	45.4 (41.2-49.6)	65.8 (60.5-70.5)	80.8 (74.2-86.2)	65.5%
Gabon	42.2 (37.1-47.7)	54.5 (50.3-58.6)	59.2 (51.1-67.4)	59.7 (44.2-74.5)	0.5%
Ghana	51.9 (48.4-55.4)	62.2 (58.6-65.8)	69 (65.2-72.5)	75.7 (70.4-80.4)	5.1%
Guinea	42.8 (38.2-47.1)	45.2 (41.4-48.8)	49.9 (44.9-54.1)	53.3 (45.3-60.7)	0%
Guinea-Bissau	41.1 (24.8-56.8)	48.6 (41.4-56.3)	60.4 (54.3-66.1)	70 (57-80.5)	3.4%
Kenya	58.5 (54.7-62.3)	66 (62.8-69.1)	73.4 (68.8-77.6)	80.9 (74.8-85.7)	71.9%
Lesotho	67.4 (62.7-72)	72.2 (69.5-74.9)	74.8 (70.5-78.8)	76.3 (67.5-83.5)	21.4%
Madagascar	47.5 (41.9-53.7)	53.5 (48.8-57.9)	56.5 (51.2-62.4)	61.8 (54-69.7)	0%
Malawi	61.9 (57.8-65.5)	70.4 (67.8-73)	79.5 (77-81.7)	85.3 (82.4-88)	99.9%

Mali	36.9 (33-40.8)	46.6 (43-50.3)	55.1 (51-59.2)	63.9 (57.7-69.8)	0%
Mauritania	36 (23.6-49.9)	45.6 (39.4-51.2)	56.3 (48.2-64.1)	69.2 (50.9-82.7)	7.8%
Mozambique	52.4 (46.7-58.1)	57.3 (52.7-62)	71.5 (64.4-78.1)	79.8 (69.3-87.9)	50.1%
Namibia	71.5 (67.4-75)	75.9 (72.6-78.7)	79.5 (70.9-86.6)	82.6 (69.4-92)	77.9%
Niger	33.6 (29.6-38)	49.9 (45.8-54)	66.6 (60.1-72.7)	79.2 (71.5-85.4)	40.5%
Nigeria	37.2 (33.7-40.5)	43.1 (40.2-45.9)	49 (44.2-54.3)	57.4 (47.7-67.2)	0%
Rwanda	36.7 (32.9-40.6)	59.1 (55.8-62.6)	75.6 (72.5-78.8)	87.2 (83.8-89.9)	100%
Senegal	40.3 (35.6-45.1)	51.5 (48.7-54.3)	63.9 (61.1-66.7)	75.4 (71.1-79.5)	3.0%
Sierra Leone	44.4 (31.6-57.4)	59.5 (56.1-62.8)	74.9 (71.5-78.1)	85.4 (80.6-89.5)	98.5%
South Africa	74.3 (70.1-78.2)	74 (69.1-78.1)	75.9 (71.1-80.1)	76.5 (69.6-82.4)	18.7%
Togo	38.8 (34.4-43.3)	49.4 (45.9-53.3)	56.1 (51.5-60.7)	63.7 (55.7-71)	0%
Uganda	51.6 (47.1-56)	61.4 (58.1-64.3)	70.4 (65.4-74.6)	77.8 (70.9-83.3)	26.3%
Zambia	60.8 (56.1-64.9)	65 (60.4-69.5)	78.8 (74.6-82.3)	85.9 (80.6-89.8)	97.7%
Zimbabwe	50.2 (43.2-57.5)	67.2 (63.8-70.4)	81.2 (77.5-84.5)	89.4 (85.4-92.6)	100%
**Northern Africa**	**55 (54.2-55.8)**	**65.3 (64.9-65.7)**	**74.4 (74-74.8)**	**81.7 (81.2-82.2)**	
Egypt	70.8 (67.3-73.9)	78 (74.8-80.9)	82.6 (78.7-85.9)	88.1 (81.9-92.5)	99.0%
Morocco	56.8 (46.9-66.7)	69.2 (59.7-78.9)	76.3 (62-87.8)	82 (60.8-94.9)	68.6%
Sudan	31.8 (20-45.8)	51.2 (45.9-56.1)	59.1 (49.2-68.7)	73.6 (58.9-85.4)	18.1%
**Latin America and the Caribbean**	**67 (66.3-67.7)**	**73.3 (72.9-73.7)**	**78.8 (78.5-79.1)**	**83.4 (83-83.9)**	
Colombia	67.3 (63-71)	78 (74.4-80.8)	84.6 (80.3-88.4)	90.9 (85.5-94.9)	99.8%
Costa Rica	81.5 (74.4-87.6)	83 (80-85.7)	83.3 (79.7-86.4)	83.5 (75.5-89)	86.1%
Dominican Republic	75.6 (72.9-78.1)	77.8 (75.7-79.9)	79 (76.5-81.3)	79.6 (74.5-84)	42.3%
El Salvador	59.3 (27.5-84.9)	76.8 (65.6-85.4)	88 (83-91.9)	95.5 (83.8-99.5)	96.3%
Guatemala	56.5 (51.3-61.5)	66.6 (62.5-70.2)	75.3 (70.4-79.8)	82.5 (75.9-88.2)	80.1%

Haiti	40.9 (37-44.6)	49.2 (45.2-53)	57 (52-62)	65.3 (56.1-73.2)	0%
Honduras	71.4 (66.4-76)	77.9 (74.8-80.6)	83.4 (78.9-87.1)	86.3 (82.3-89.5)	99.9%
Mexico	70.2 (53.4-84)	76.2 (69.8-81.5)	80.5 (75-85.3)	84.1 (71.9-92.3)	79.5%
Nicaragua	73.9 (68.1-79.1)	78.4 (70.4-85.2)	81.5 (67.7-91.7)	82.7 (63.1-94.6)	68.3%
Panama	69.8 (48.2-84.9)	76 (69.3-81.5)	80.9 (73.6-86.8)	83.8 (67.3-95.3)	74.6%
Paraguay	67.3 (47.6-83.6)	75 (67.8-81.1)	81.2 (75.6-85.9)	85.2 (73.4-93.4)	83.3%
Peru	64.2 (61.6-66.8)	68.8 (67.2-70.2)	72.2 (69.5-74.6)	75.8 (72.5-78.9)	0.2%
**Southern Europe**	**70.1 (69.4-70.8)**	**65.4 (65-65.8)**	**60.4 (59.9-60.8)**	**55.2 (54.4-55.9)**	
Albania	72.4 (64.7-79.7)	63.8 (59.8-67.6)	62.4 (56.8-67.5)	54.5 (42.8-65.9)	0%
Bosnia and Herzegovina	68 (45.9-83.9)	67.4 (62.2-72.3)	66.6 (46.9-82.4)	62.4 (23.6-91.5)	20.6%
Montenegro	65.2 (44.8-82)	63.5 (56.9-69.8)	67 (56.2-77)	61.6 (33.3-85.4)	9.8%

CrI, credible interval

Table S8: Country-specific composite coverage index by place of residence, 2000-2030

	Urban (%, 95% CrI)				Rural (%, 95% CrI)			
**country**	**2000**	**2010**	**2020**	**2030**	**2000**	**2010**	**2020**	**2030**
**Southern Asia**								
Afghanistan	36 (18.5-56)	50.8 (41.7-59.9)	63 (53.8-71.6)	73.4 (54.8-87.9)	25.6 (12.3-42.5)	38.3 (30.3-46)	50.6 (42.1-60.3)	62.8 (43-81.2)
Bangladesh	59 (55.6-62.6)	69.3 (66.9-71.8)	77.8 (74.9-80.7)	84.7 (80.1-88.7)	47.1 (43.5-50.8)	58.3 (55.3-61.2)	68.5 (64.6-72.1)	77.4 (71-83)
India	63.7 (59.5-67.7)	73.8 (70.7-76.7)	82.2 (78.5-85.6)	89 (84.3-92.6)	48.5 (44.1-52.9)	60.2 (56.1-64.1)	71.3 (66.1-76.4)	81.3 (74.4-87.3)
Maldives	71.3 (61-80.6)	73.8 (69.6-77.4)	78.5 (71-85)	74.3 (63-83.1)	69.9 (59-79.8)	72.4 (68-76.3)	77.3 (69-83.9)	73 (61.6-82)
Nepal	57.1 (52.5-61)	68 (65.6-70.3)	77.6 (74.7-80.1)	84.7 (81.7-87.2)	45.6 (41.7-49.9)	57.2 (54.6-59.9)	68.6 (65-71.4)	77.7 (73.5-81.4)
Pakistan	52.9 (42.5-63.2)	62 (58.1-65.8)	71.6 (67.2-75.9)	80.4 (73.6-86.5)	38.8 (28.4-48.4)	47.8 (43.9-51.8)	58.6 (53-64.1)	69.7 (60.3-78.1)
**South-eastern Asia**								
Cambodia	44.6 (40.1-48.7)	66.4 (63-69.5)	83.1 (79.8-86.2)	92.4 (89.4-94.8)	37.2 (33.3-41.3)	59.2 (55.9-62.2)	78.3 (74-82.1)	89.9 (86-93)
Indonesia	70.8 (66.7-74.6)	76 (73.2-78.8)	80.7 (77.8-83.7)	84.3 (79.8-88.4)	64.1 (59.5-68.1)	70.1 (66.9-73.2)	75.6 (72.2-78.9)	79.9 (74.5-85)
Myanmar	65.2 (40.1-83.7)	70.2 (51.6-84.3)	81 (74.2-86.9)	85.6 (74-93.7)	49.4 (25.5-72.3)	54.7 (35.1-74.8)	68.2 (57.2-77)	75.1 (58.4-87.9)
Philippines	63.9 (60.4-67.4)	70.8 (67.9-73.6)	76.9 (73.8-80.3)	82.1 (76.9-86.2)	53.8 (49.9-57.8)	61.4 (58.1-64.7)	68.7 (64.6-72.7)	75.1 (68.5-80.5)
Thailand	81.4 (68.8-90.2)	87.4 (84.3-90)	90.1 (86.8-92.6)	92.5 (85.8-96.5)	80.5 (67.7-89.5)	86.7 (83.5-89.4)	89.5 (86-92.4)	92.1 (84.6-96.4)
Timor-Leste	55.3 (39.8-68.6)	64.1 (59.8-68.6)	73.3 (66.6-79.6)	77.6 (64.3-87.8)	44.7 (30.1-58.7)	53.6 (48.9-58.5)	64 (56.2-71.2)	69.4 (54-82.3)
**Central Asia**								
Kazakhstan	73.5 (69.3-77.5)	87.4 (84.8-89.4)	92.6 (90-94.8)	95.8 (93.2-97.6)	67.6 (62.9-72.5)	83.9 (80.6-86.6)	90.4 (86.9-93.2)	94.5 (90.9-96.9)
Tajikistan	66.6 (53-77.9)	71.8 (66.4-76.8)	76.3 (71.2-80.8)	79.6 (69.8-87.3)	60.6 (46.2-73)	66.1 (59.7-71.6)	71.1 (65.1-76.8)	74.9 (63.4-84.7)
**Eastern Asia**								
Mongolia	78.8 (71.9-85.1)	80.3 (77-83.2)	81.6 (78.1-84.7)	83.8 (76.5-89.4)	76.9 (69.9-83.4)	78.4 (75.3-81.5)	79.8 (76-83.2)	82.2 (74.6-88.2)
**Western Asia**								
Armenia	56.8 (52.4-61.1)	70.1 (67.3-72.8)	80.5 (77.3-83.7)	87.8 (84.3-91.1)	50.3 (46.2-55)	64.4 (61.2-67.6)	76.1 (71.9-80)	84.8 (80.1-88.8)
Azerbaijan	81.3 (37.1-98)	22.2 (3-64.3)	31.7 (4.7-78)	28.7 (1.2-87.5)	74.6 (27.9-96.8)	16 (2.1-52.8)	23.9 (3-69.1)	22.4 (0.7-81.8)

Iraq	71.1 (57.5-83.2)	68 (64.3-71.8)	71.3 (63.5-78.7)	73.9 (62.9-83.2)	63.9 (49.1-78.2)	60.3 (56-64.5)	64.1 (55.1-73.1)	67 (55.1-78.6)
Jordan	68.2 (63.5-72.9)	75.3 (72.2-78.2)	76.4 (72.5-80)	81.4 (75.8-86)	64.7 (58.7-70)	72.3 (68.6-75.4)	73.4 (68.9-77.4)	78.9 (72.9-84.1)
Yemen	44.7 (25.5-64.1)	55.9 (47.7-63.9)	48.6 (31.5-65.5)	58.7 (31.2-81.2)	28.6 (15-46.8)	38.2 (29.5-46.4)	31.8 (18.8-47.3)	42 (19.5-67.6)
**Sub-Saharan Africa**								
Angola	92.6 (72.4-99.3)	60.9 (49.5-70.7)	87.2 (75.8-95.1)	90.2 (78.1-97.2)	84 (49.1-98.2)	37.8 (27.1-48.5)	73 (52-88.4)	78.6 (56-93)
Benin	51.2 (47.3-55.2)	53.1 (50.3-56)	56.9 (52.7-61.2)	66.5 (55.3-75.8)	43.1 (39-47.2)	44.9 (42.1-47.8)	48.7 (44.4-53.1)	58.9 (47.3-68.8)
Burkina Faso	51.5 (47-56)	69 (64.7-73.1)	82.5 (76.6-87.5)	90.4 (85-94.5)	28.4 (25-32)	45.4 (40.9-50.3)	63.9 (55.5-72.6)	77.9 (68.1-86.6)
Burundi	54.8 (44.1-64.7)	62.9 (58.2-67.5)	69.6 (63.7-75.2)	75.5 (65.1-84.3)	47.6 (37.1-58)	55.8 (50.9-60.6)	63.1 (56.6-69.2)	69.8 (58.5-79.8)
Cameroon	52.9 (49.2-56.7)	57 (53.8-60)	60.4 (56-64.6)	66.3 (60.4-71.5)	35.4 (32.1-39.1)	39.2 (36.1-42.2)	42.6 (38.1-47.3)	48.9 (42.6-55.3)
Central African Republic	45 (36-54.5)	48.1 (43-53.2)	50.1 (44.8-55.6)	55.4 (44.2-67.1)	23.8 (17.4-32.1)	26 (22.3-30.3)	27.6 (22.9-32.6)	32.3 (22.4-43.7)
Chad	26.1 (21.3-31.1)	36.3 (32.4-40.1)	50.7 (42.8-58.5)	64.6 (51.6-76.1)	10.6 (8.3-13.2)	16 (13.7-18.4)	25.6 (19.9-31.7)	38.3 (26.3-51.2)
Comoros	48.3 (33.3-64.1)	56.5 (50.4-62.6)	65.1 (54.1-74.6)	72.3 (51.5-87.6)	38.7 (24.6-54.6)	46.5 (39.5-53)	55.6 (44-66.1)	63.9 (40.8-81.9)
Democratic Republic of the Congo	49 (42.3-55.5)	51.4 (48.1-54.7)	53.1 (47.5-58.7)	53.8 (42.8-64.8)	36.2 (30-42.6)	38.5 (35.4-41.7)	40 (34.8-45.5)	40.9 (30.1-52)
Ethiopia	43.3 (38.9-47.4)	59.7 (55.9-63.5)	77.4 (73.1-81.5)	88.2 (83.6-92.1)	16.5 (14.2-19)	27.7 (24.4-31)	47.1 (41.3-53)	66.1 (57.1-74.5)
Gabon	48.8 (43.4-53.9)	61 (56-65.7)	65.5 (57.8-72.5)	65.8 (51.1-78.3)	35 (30.3-40)	46.8 (41.8-52.3)	51.8 (44.1-59.5)	52.4 (37.2-66.9)
Ghana	57.2 (53.4-60.6)	67.2 (63.9-70.3)	73.5 (70.1-76.7)	79.4 (74.9-83.7)	46.4 (42.9-49.8)	56.9 (53.5-60.2)	64.1 (60.1-68)	71.4 (66-76.8)
Guinea	53.2 (49-57.4)	55.7 (52.2-59.3)	60.2 (55.9-64.7)	63.5 (56.6-70.9)	31.6 (27.8-35.7)	33.7 (30.6-37)	38.1 (33.9-42.6)	41.4 (34.7-49.3)
Guinea-Bissau	47.8 (31-64.5)	55.6 (48-63.2)	66.9 (61.9-71.9)	75.5 (64.5-84.4)	34.5 (20.6-50.7)	41.6 (34.6-49.1)	53.4 (47.4-59.2)	63.7 (49.9-75.7)
Kenya	65.6 (62-69.3)	72.4 (69.6-75.1)	78.9 (74.8-82.4)	85.1 (80.4-89.3)	52.6 (48.4-56.5)	60.4 (56.9-63.6)	68.4 (62.9-73.3)	76.9 (69.9-83.2)
Lesotho	72.3 (68.2-76.5)	76.7 (74.2-78.9)	79 (75.3-82.2)	80.3 (72.8-86.2)	63.2 (58.1-67.9)	68.3 (65.4-71.4)	71.1 (66.6-75.1)	72.8 (63.9-80.6)
Madagascar	55.4 (49.5-61.1)	61.2 (57.2-65.3)	64 (59-69.2)	68.9 (61.6-75.6)	40 (34.3-46.1)	45.8 (41.7-50.4)	48.9 (43.3-54.4)	54.4 (46.1-62.6)
Malawi	65.6 (62.2-69)	73.6 (71.1-76.2)	81.9 (79.5-83.9)	87.2 (84.2-89.5)	58.8 (55-62.4)	67.5 (64.6-70.3)	77.2 (74.7-79.6)	83.6 (80.3-86.4)
Mali	46.8 (42.9-50.6)	56.7 (53.3-60.3)	64.8 (60.8-68.4)	72.7 (67.1-77.4)	26.7 (23.8-29.9)	35.2 (32-38.6)	43.3 (39-47.4)	52.5 (45.7-58.7)
Mauritania	43.4 (30.6-57.8)	53.6 (48-58.6)	63.9 (56.6-70.9)	75.3 (57.9-87.5)	26.7 (17-38.9)	35.1 (30.2-40)	45.4 (37.3-53.9)	59.5 (39.5-76.6)

Mozambique	61.3 (55.8-66.2)	65.9 (61.4-69.9)	78.3 (72.2-83.7)	85 (76.2-91.5)	43 (37.4-48.2)	47.9 (43.1-52.5)	63.2 (55.8-70.8)	73.1 (61.1-83.1)
Namibia	76.9 (73.4-80)	80.7 (77.9-83)	83.7 (76.1-89.6)	86.2 (73.9-94)	66.3 (62.3-70.6)	71.2 (67.7-74.8)	75.4 (64.2-83.9)	78.9 (61.9-90.1)
Niger	45.9 (41.2-50.5)	62.5 (58.5-66.3)	76.9 (71.4-81.5)	86.4 (81-90.6)	19 (16.1-22.1)	31.5 (28-35.4)	48 (40.8-55.5)	63.9 (53.5-73.3)
Nigeria	44.2 (40.6-47.5)	50.3 (47.1-53.3)	56.2 (51.3-61.3)	64.3 (54.5-73.4)	28.5 (25.6-31.5)	33.7 (31.2-36.5)	39.3 (34.8-44.3)	47.6 (37.9-57.9)
Rwanda	40.7 (37-44.7)	63.2 (60.3-65.9)	78.6 (75.7-81.3)	89 (85.9-91.3)	33.5 (30.2-37.3)	55.8 (52.8-58.8)	73 (69.5-76.1)	85.6 (81.8-88.5)
Senegal	47.8 (43.1-52.6)	59.1 (56.5-61.7)	70.7 (68.1-73)	80.6 (76.7-84.2)	33.6 (29.2-38.3)	44.4 (41.7-47)	57.1 (54.1-59.9)	69.7 (64.7-74.6)
Sierra Leone	49 (35.5-62.5)	64 (61-67.1)	78.3 (75.6-81)	87.6 (83.8-90.9)	40.9 (28.4-53.9)	55.9 (52.6-59.2)	72.1 (68.5-75.2)	83.5 (78.5-87.6)
South Africa	78.1 (74.6-81.4)	77.9 (74.4-81.1)	79.5 (74.8-84.1)	80 (73.3-86.4)	72.1 (67.3-76.3)	71.8 (67.5-75.6)	73.8 (67.5-79.2)	74.4 (65.7-81.9)
Togo	46.1 (41.5-50.5)	56.9 (53.1-60.5)	63.3 (58.6-67.6)	70.2 (62.6-76.6)	31.3 (27.3-35.3)	41.3 (37.7-45)	47.9 (42.8-52.6)	55.8 (47.4-63.7)
Uganda	59.1 (54.9-63.1)	68.4 (65.2-71.2)	76.3 (72.4-80.1)	82.6 (77.1-87.4)	43.9 (39.7-48.3)	54 (50.4-57.2)	63.6 (58.8-68.3)	72.1 (63.9-78.8)
Zambia	68 (64.1-72)	71.7 (67.5-75.5)	83.5 (80.4-86.2)	89.2 (85.3-92.1)	54 (49.2-58.8)	58.4 (53.6-63.3)	73.8 (69.5-77.7)	82.2 (76.3-86.9)
Zimbabwe	55.7 (48.4-63.6)	72 (69.3-74.8)	84.4 (81.2-87.2)	91.4 (87.9-94)	45.9 (38.8-54)	63.3 (60-66.5)	78.4 (74.2-82.2)	87.7 (83-91.6)
Northern Africa								
Egypt	74.6 (71.2-77.8)	81.1 (78.4-83.6)	85.2 (81.9-88.3)	90 (84.9-93.9)	67.3 (63.4-70.7)	75 (71.8-77.9)	80.1 (75.8-83.9)	86.3 (79.8-91.5)
Morocco	63.6 (56.1-70.4)	74.7 (63.2-84.2)	80.7 (63.7-91.7)	85.3 (60.9-96.6)	49 (41.1-56.7)	62.1 (46.7-75.6)	70 (49.2-86.2)	76.7 (46.2-94.2)
Sudan	38 (25.7-51.7)	58.2 (53.3-62.7)	65.7 (57.1-74.3)	78.6 (65.7-88.6)	25 (16-35.9)	42.9 (38-47.7)	51 (41.6-60.5)	66.8 (51-81)
**Latin America and the Caribbean**								
Colombia	72.4 (68.7-75.6)	81.8 (78.9-84.3)	87.5 (83.5-90.9)	92.7 (88.4-96)	62 (57.7-66.2)	73.7 (69.8-77.1)	81.4 (76-85.9)	88.8 (82.7-93.6)
Costa Rica	83.4 (76.8-89.1)	84.7 (81.7-87.4)	85 (81.7-87.8)	85.2 (78.7-90.2)	80.7 (73.1-86.9)	82.2 (78.3-85.2)	82.6 (79-86)	82.8 (75.3-88.6)
Dominican Republic	76.5 (74-78.9)	78.6 (76.4-80.6)	79.8 (77.2-82.2)	80.3 (75.6-84.6)	76 (73.4-78.5)	78.2 (75.9-80.1)	79.4 (76.8-81.7)	79.9 (75.1-84.1)
El Salvador	63.1 (29.6-87.6)	79.7 (67.4-88.4)	89.8 (85.7-92.9)	96.3 (87.3-99.6)	56.8 (23.4-84.9)	74.6 (60.6-85.1)	86.8 (81.5-90.8)	95.2 (83.5-99.4)
Guatemala	62.4 (57-67.1)	71.8 (67.4-75.5)	79.6 (74.7-83.5)	85.7 (80.3-90.4)	49.1 (43.7-54.5)	59.7 (54.9-64.9)	69.4 (62.9-75.3)	77.8 (69.4-84.7)
Haiti	47.4 (42.9-51.3)	55.7 (51.5-59.6)	63.2 (58.3-67.9)	71 (62.5-78.1)	34.9 (31.2-38.8)	42.9 (38.7-46.5)	50.6 (45.6-55.6)	59.4 (49.9-67.6)
Honduras	75.1 (70.3-79.2)	81 (78.4-83.3)	85.9 (82.2-88.8)	88.4 (84.9-91.1)	68.3 (63.4-72.9)	75.3 (72.3-78.1)	81.2 (76.6-85)	84.5 (79.7-88.1)

Mexico	73.2 (57.5-86.4)	78.9 (72.8-84.4)	82.8 (77.7-87.3)	86.1 (76.2-93.3)	69.2 (51.6-83.6)	75.4 (68.3-82)	79.7 (73.2-85.2)	83.5 (70.7-91.9)
Nicaragua	78.8 (74.5-82.4)	82.6 (75.6-88.7)	85.1 (72.1-93.8)	86 (68-96.2)	68.8 (63.2-74.4)	73.9 (63.7-82.6)	77.4 (59.7-90.2)	78.8 (55.1-93.9)
Panama	75.4 (52.3-88.7)	81 (74-86.2)	85 (79-89.6)	87.5 (74.7-96)	63.7 (38.3-82.1)	70.4 (60.4-78.6)	76 (66.9-83.4)	79.9 (61.4-92.8)
Paraguay	70.8 (50.1-86)	78 (69.8-84.3)	83.7 (79.5-87.4)	87.3 (77.6-93.9)	65.4 (43.3-83.6)	73.3 (63.8-81.6)	79.9 (74.4-84.5)	84.2 (72.8-92)
Peru	69.5 (66.9-71.9)	73.7 (72.4-75)	76.7 (74.3-79)	80 (76.8-82.7)	58.6 (55.8-61.2)	63.5 (61.8-65)	67.1 (64.2-70.1)	71.2 (67.5-74.9)
**Southern Europe**								
Albania	75.5 (67.8-82.5)	67.6 (63.6-71.5)	66.2 (60.8-71.5)	58.5 (46.9-69.6)	68.6 (59.5-77)	59.6 (54.9-64.2)	58.1 (52.1-63.8)	50 (38.1-61.1)
Bosnia and Herzegovina	70.5 (47.2-87.3)	70.2 (64.9-74.7)	69.4 (51.5-83.4)	65.1 (28-91.8)	65.2 (41.6-83.9)	64.6 (58.6-70.4)	63.9 (44.8-80.3)	59.9 (22.3-90.3)
Montenegro	71.1 (50.6-86.4)	69.8 (63.5-75.6)	73 (64-80.8)	67.9 (40.9-88.1)	58.9 (35.5-78.7)	56.9 (49.2-64.3)	60.7 (50.1-70.6)	55.5 (28.6-79.8)

CrI, credible interval

Figure S2. Detail year-specific observed and predicted CCI by area of residence (national, urban, and rural)

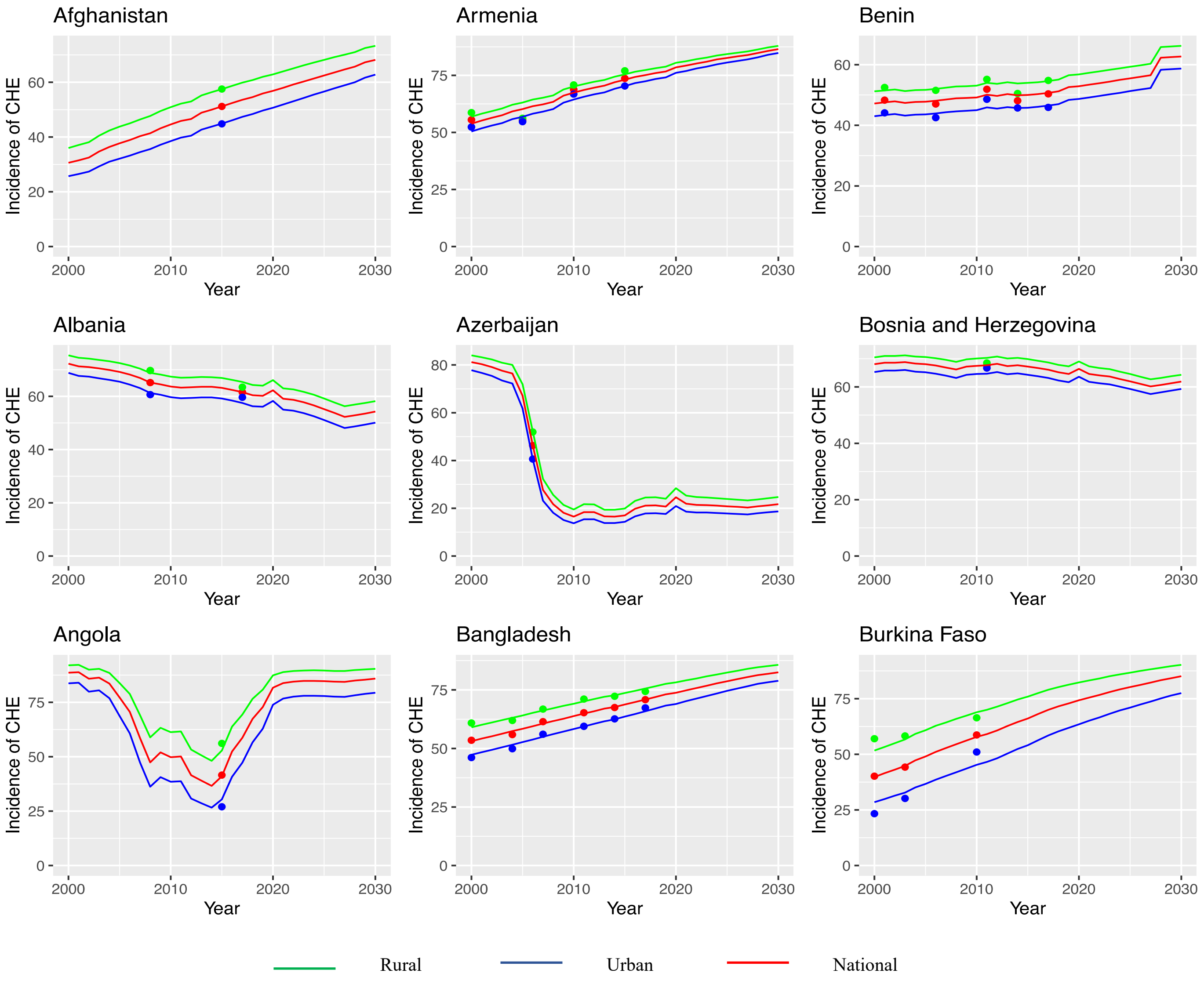

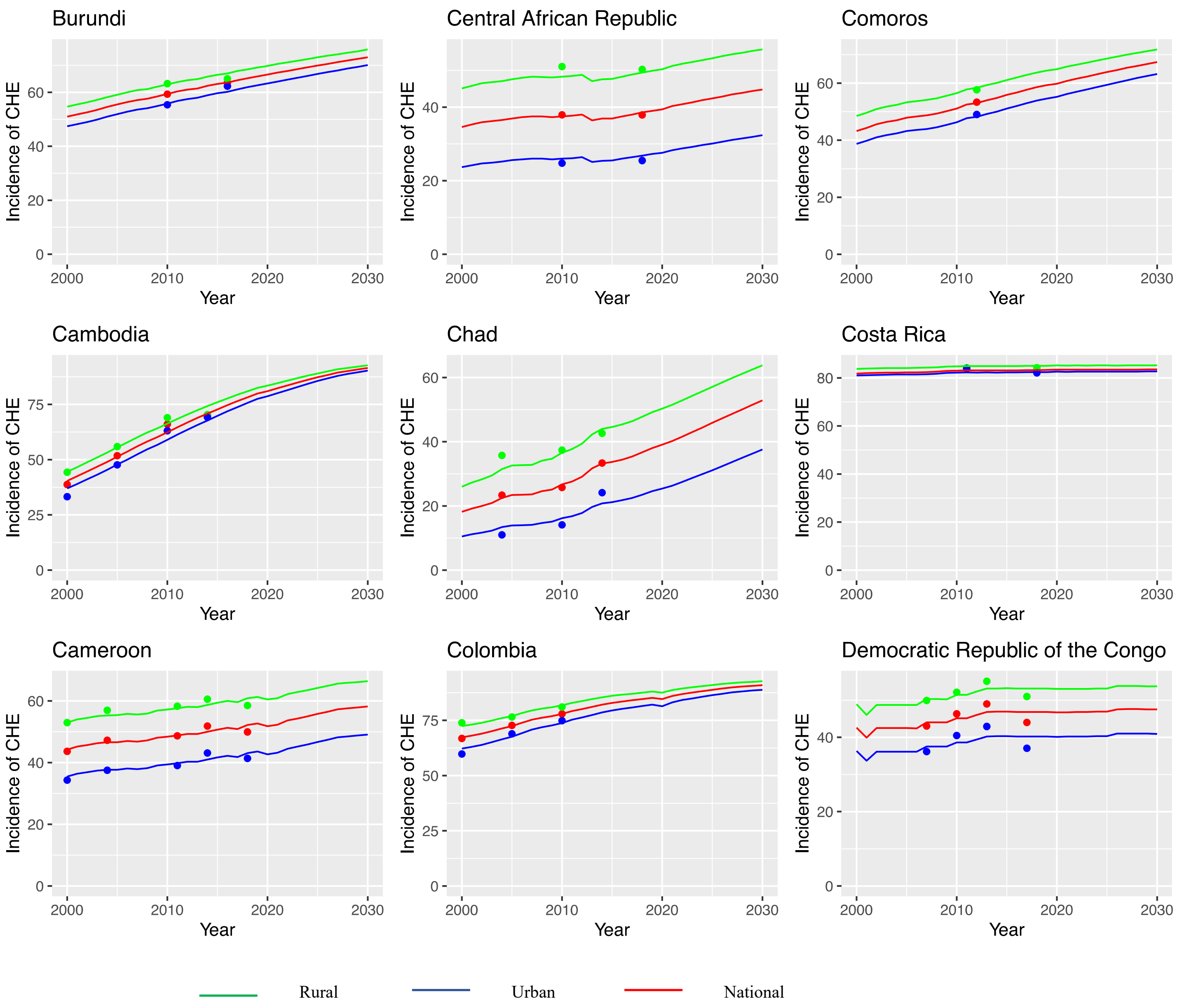
Burundi
Central African Republic
Comoros
Cambodia
Chad
Costa Rica
Cameroon
Colombia
Democratic Republic of the Congo
Incidence of CHE
Year
Rural
Urban
National

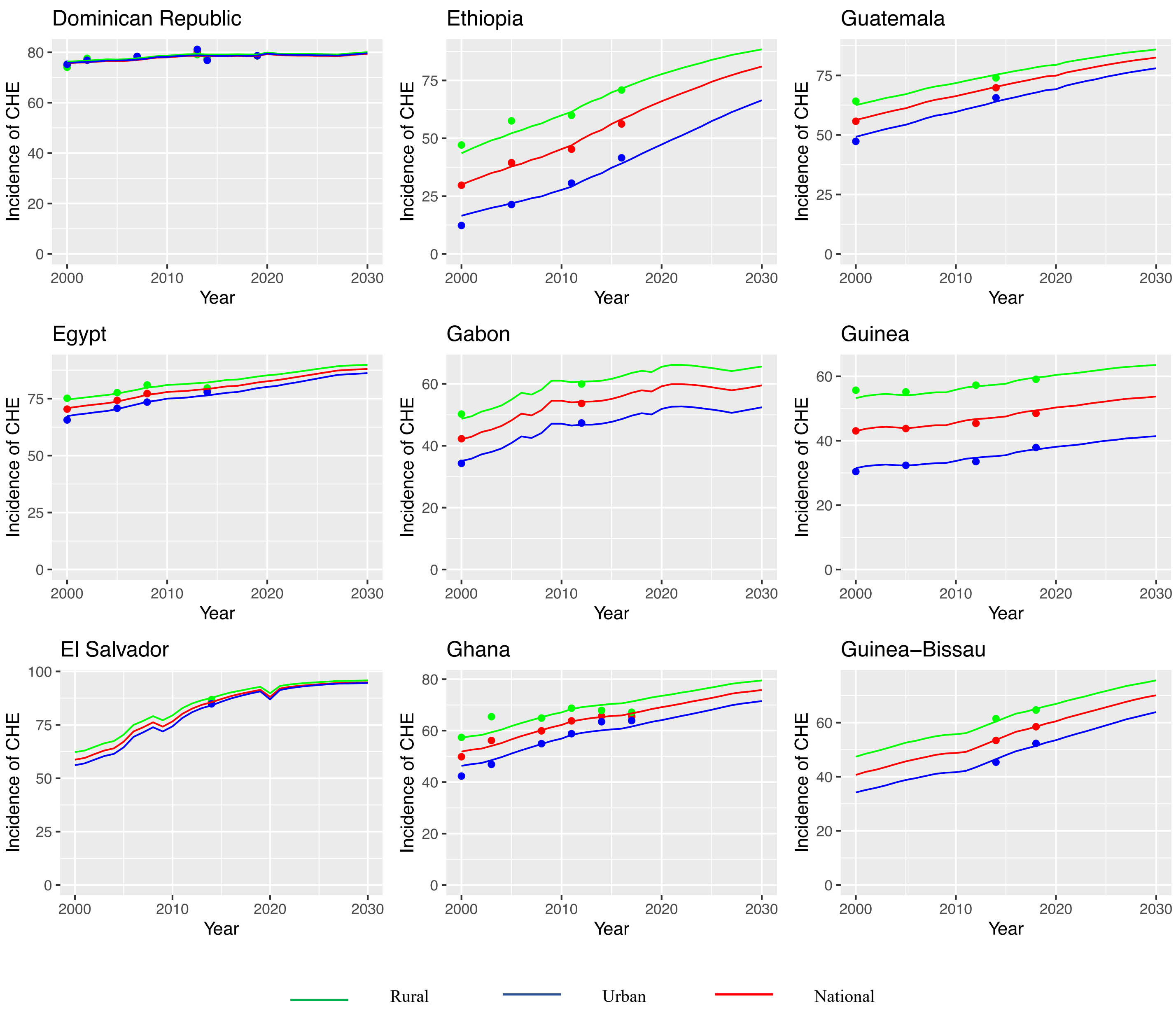

Dominican Republic
Ethiopia
Guatemala
Egypt
Gabon
Guinea
El Salvador
Ghana
Guinea–Bissau
Incidence of CHE
Year
Rural
Urban
National

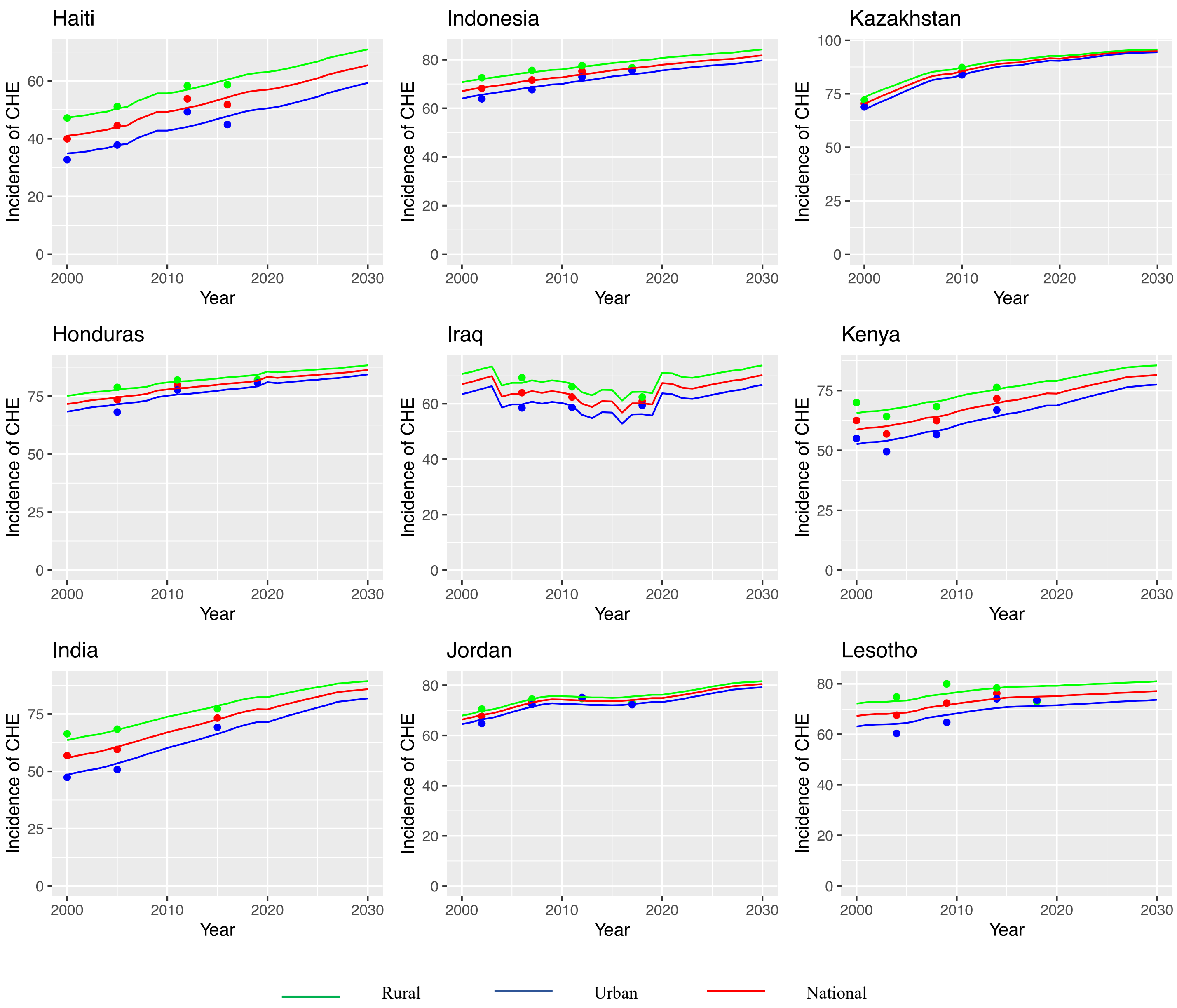
Haiti
Indonesia
Kazakhstan
Honduras
Iraq
Kenya
India
Jordan
Lesotho
Incidence of CHE
Year
Rural
Urban
National

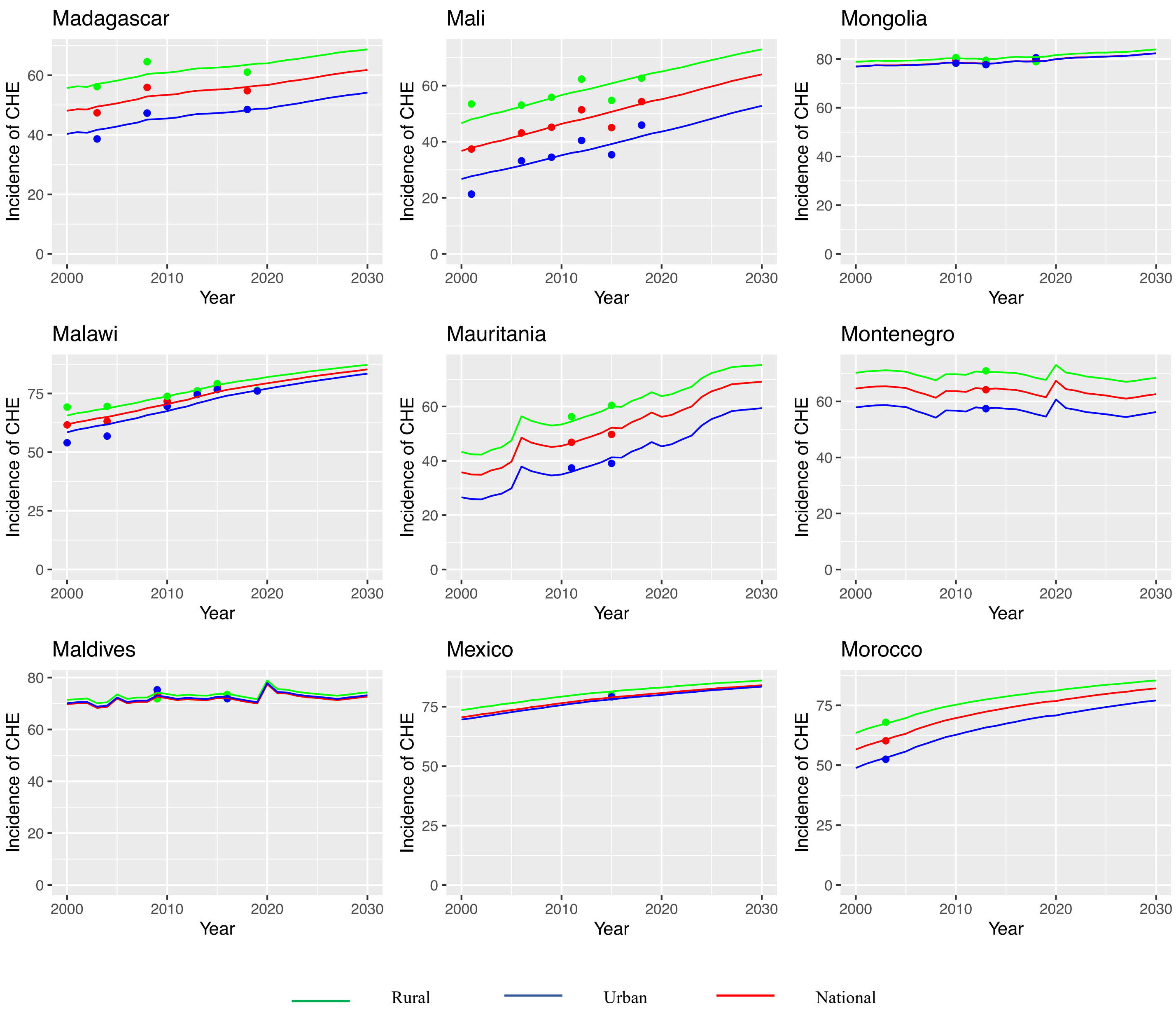
Madagascar
Mali
Mongolia
Malawi
Mauritania
Montenegro
Maldives
Mexico
Morocco
Incidence of CHE
Year
Rural
Urban
National

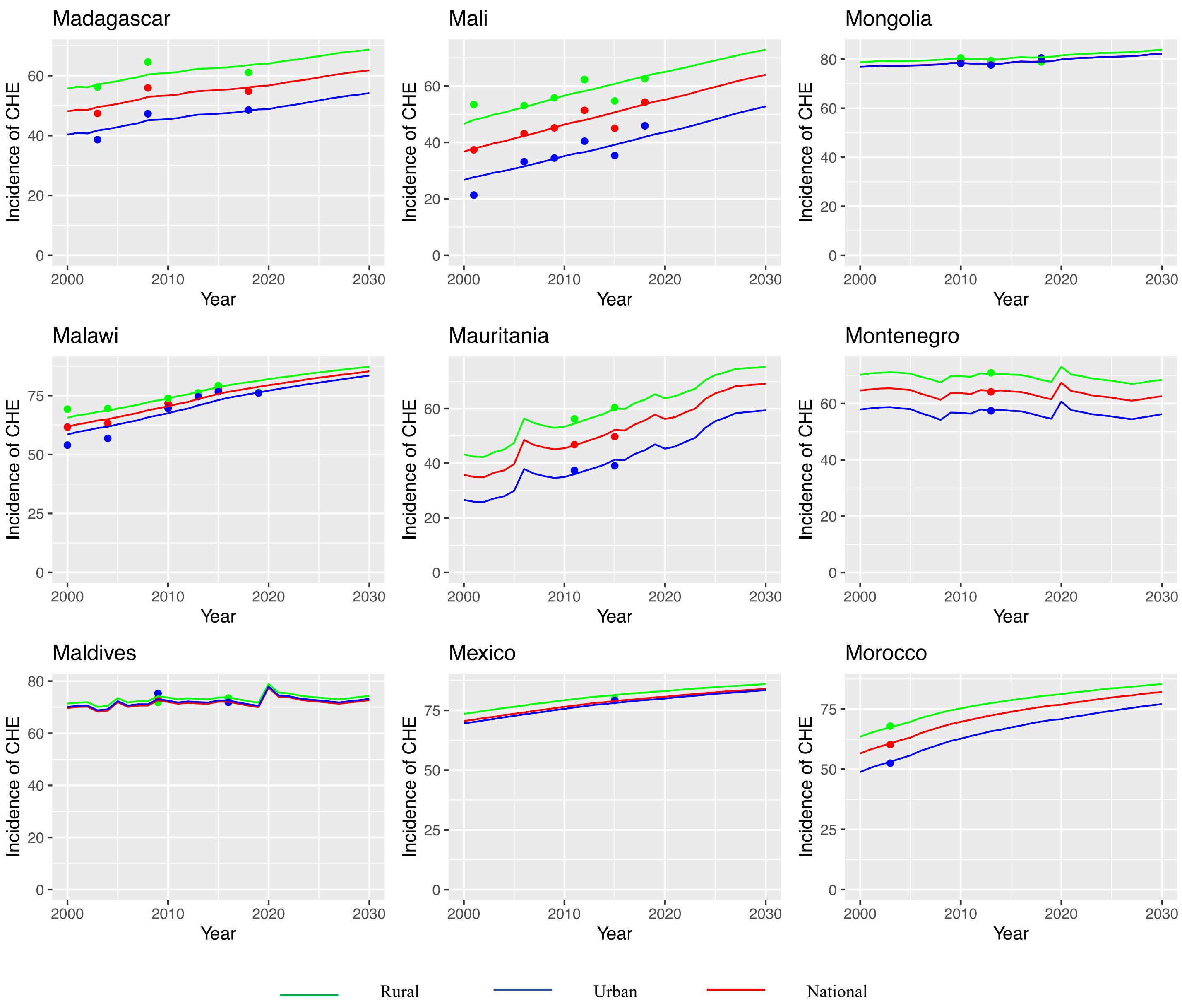
Madagascar
Mali
Mongolia
Malawi
Mauritania
Montenegro
Maldives
Mexico
Morocco
Incidence of CHE
Year
Rural
Urban
National

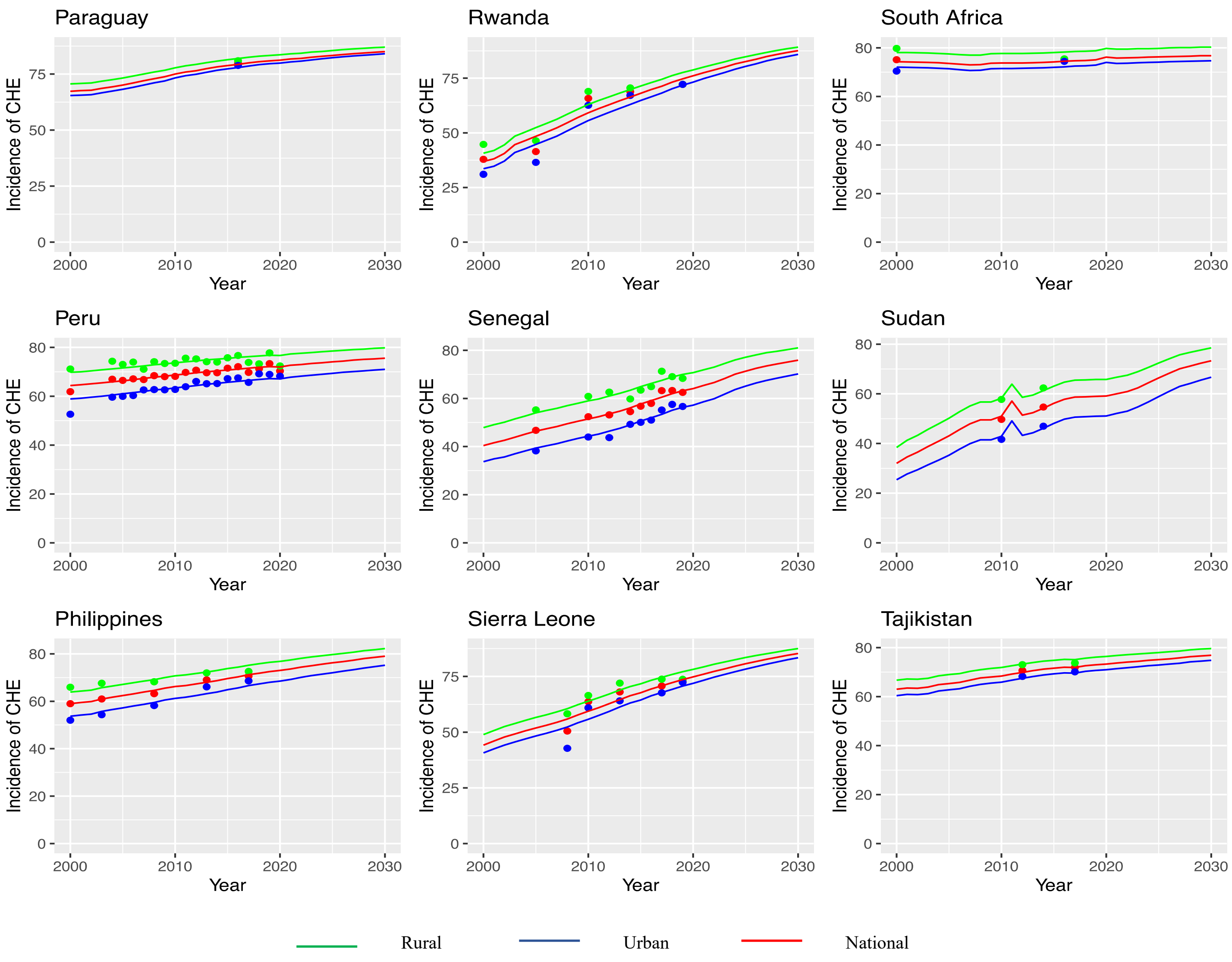
Paraguay
Rwanda
South Africa
Peru
Senegal
Sudan
Philippines
Sierra Leone
Tajikistan
Incidence of CHE
Year
Rural
Urban
National

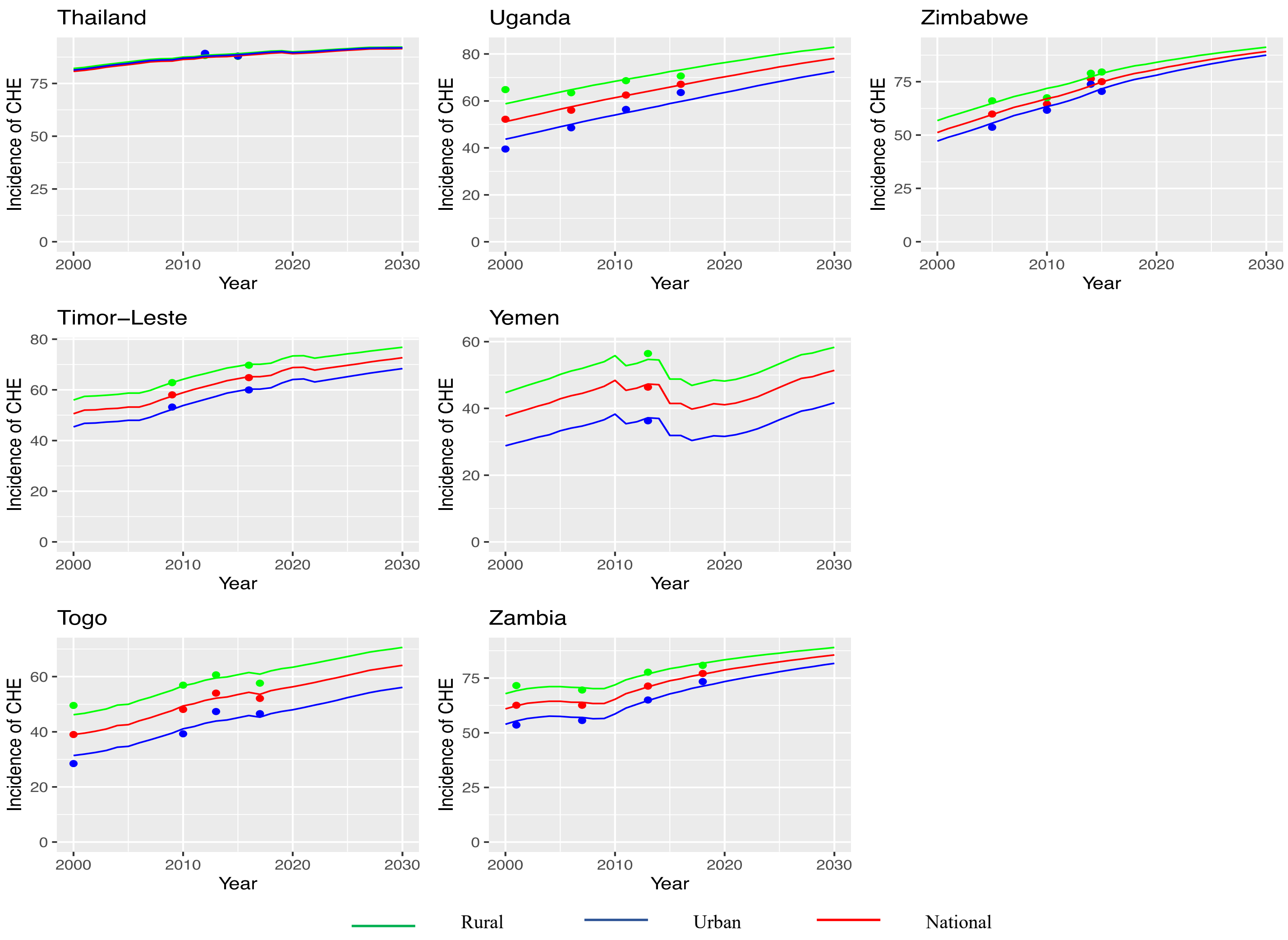
Thailand
Uganda
Zimbabwe
Timor–Leste
Yemen
Togo
Zambia
Incidence of CHE
Year
Rural
Urban
National

Table S9: Country-specific composite coverage index by educational status, 2000-2030

	No education (%, 95% CrI)				Secondary or higher education (%, 95% CrI)			
**country**	**2000**	**2010**	**2020**	**2030**	**2000**	**2010**	**2020**	**2030**
Afghanistan	28.4 (12.4-49.7)	39.8 (30.8-49.4)	49.6 (39.5-58.9)	59.5 (36.5-79.3)	44.4 (22.4-66.8)	57.8 (48.3-66.8)	67.1 (58.1-75)	74.8 (54.1-88.9)
Albania	59.5 (46.5-70.7)	51 (45.2-57.1)	49.7 (41.2-58.1)	42.8 (27.5-59.1)	74.1 (63-83.6)	67.1 (62.7-71.2)	66 (59.3-71.9)	59.2 (44.8-73.3)
Angola	76.7 (7.8-99.9)	41.5 (24.5-61.7)	70.4 (23.2-97)	74.5 (29.5-97.6)	85.8 (19.3-100)	66.5 (47.8-81.8)	85.1 (47.3-98.9)	87.7 (54.6-99.2)
Armenia	28.6 (21-36.9)	48.1 (38-58)	63.9 (51.5-74.5)	78.1 (65-87.6)	47.6 (42.1-53.2)	67.9 (64.7-71.1)	80.2 (74.7-84.6)	89.1 (83.1-93.2)
Azerbaijan	70 (8.2-99.6)	22.6 (0.4-81.8)	27.8 (0.7-86)	27.4 (0.1-94.8)	74.9 (12.1-99.7)	27.5 (0.5-87.6)	33.5 (1-90.2)	31.8 (0.1-96.2)
Bangladesh	43.5 (39.3-47.5)	52.7 (49.8-55.8)	63.6 (58.8-68.3)	75.3 (65.3-83.9)	58.1 (54.1-62)	66.8 (64-69.4)	75.8 (72.1-79.3)	84.5 (77.2-90.4)
Benin	44.5 (40.2-48.6)	44.6 (41.3-47.6)	45.2 (40-50.4)	44.8 (17.4-77.2)	61.7 (57.3-65.9)	61.9 (58.8-64.7)	62.5 (57.5-67)	60.6 (30-87.1)
Bosnia and Herzegovina	66.1 (31.9-91.8)	51.2 (40.5-62.3)	38.8 (13.4-70.6)	28 (1.3-82.7)	79.3 (47.3-96.2)	69.1 (63.4-74.5)	56 (27.4-80.3)	40.2 (3.1-89.5)
Burkina Faso	30.1 (26.4-34.2)	45.2 (40-50.3)	61.7 (52.1-70.5)	74.7 (62.9-84.7)	61.3 (56.8-65.5)	75.2 (71.5-78.7)	85.5 (80.2-89.7)	91.5 (86-95.2)
Burundi	46.7 (35.2-58.3)	53.9 (48.7-59.2)	60.2 (53-67)	66.2 (52.6-77.9)	59.8 (49.1-70.8)	66.7 (62.4-71.3)	72.1 (66.1-77.7)	76.9 (66.3-85.9)
Cambodia	30.6 (26.6-34.4)	51.3 (47.5-54.9)	74 (68.5-79)	88.0 (82.8-92.2)	48.9 (44.5-53.3)	69.6 (66.3-72.6)	86 (82.3-89)	94.1 (91.1-96.2)
Cameroon	25.1 (22-28.5)	28.2 (25.3-31.1)	31.2 (27.2-35.3)	37.7 (31.3-44.8)	56.5 (52.4-60.4)	60.4 (57.2-63.6)	63.8 (59.3-68)	70 (63.3-75.3)
Central African Republic	24 (16.4-32.5)	25.3 (21.2-30)	25.1 (20.5-29.8)	28.7 (19.7-39)	51.7 (40.9-62.7)	53.7 (48-59.2)	53.4 (47.6-59.6)	57.6 (46-68.9)
Chad	10.9 (8-14.3)	15.7 (13.1-18.2)	21.1 (15.8-27.1)	28.4 (17.7-40.7)	33.6 (27-40.8)	43.7 (39.4-48.1)	52.4 (44.3-60.6)	61.6 (47.4-73.8)
Colombia	54.4 (49.6-59.2)	66.5 (61.8-70.9)	75.2 (67.8-81.9)	84.3 (75.8-90.8)	73.3 (69.3-76.8)	82.1 (79-84.8)	87.5 (83.2-91.2)	92.5 (88.1-95.9)
Comoros	39.3 (23.6-56.6)	44.5 (37.9-51.9)	51.3 (39.9-63)	57.7 (35.5-79)	51.7 (33.9-67.1)	57.3 (50.6-64)	63.7 (51.6-74.6)	69.0 (47-86.3)
Costa Rica	83.2 (75.3-89.1)	82.2 (78.4-85.7)	80.8 (76.4-84.3)	78.3 (69-85.2)	86.7 (80.4-91.5)	85.9 (82.7-88.7)	84.7 (80.8-87.9)	82.6 (74.6-88.5)
Democratic Republic of the Congo	33.9 (27.7-40.8)	35.5 (32.3-39)	36.6 (31.3-42.2)	37 (27.1-47.7)	48.9 (41.9-56.1)	50.8 (47.1-54.5)	51.9 (45.8-57.9)	52.1 (41-63.3)
Dominican Republic	71 (67.9-74.2)	70.7 (67.7-73.5)	69.2 (65.6-72.8)	66.2 (59-72.7)	79.8 (77.2-82.1)	79.5 (77.4-81.5)	78.4 (75.6-81.3)	75.9 (70.4-81.4)
Egypt	64.7 (60-69.1)	71.6 (67.1-75.8)	76.7 (71.5-81.1)	82.7 (74.1-89.2)	74.4 (70.9-77.8)	80 (76.7-83.2)	83.9 (79.7-87.4)	88.3 (81.8-92.8)
El Salvador	51.3 (7-96.3)	70.6 (43.4-92.1)	85.7 (79.4-90.5)	89.9 (32.7-99.9)	57.2 (9.2-97.4)	76.5 (51.1-94.4)	89.4 (84.7-93)	91.9 (43.6-99.9)
Ethiopia	15.1 (12.9-17.5)	26.5 (22.9-30.4)	43.9 (37.1-50.5)	61.4 (50.2-71.3)	48.7 (43.7-53.6)	65.9 (61.8-69.7)	80.7 (76-84.8)	89.4 (84.2-93)

Gabon	41.2 (35.7-46.7)	50.5 (43.8-57.3)	53.7 (44.6-62.5)	53.1 (31.5-75.3)	50.8 (45.1-56.4)	60 (52.9-66.9)	63.1 (54.9-71.4)	62.1 (40-81.9)
Ghana	41.5 (37.7-45.6)	54.4 (50.3-58.4)	62.4 (57.5-66.9)	71.3 (64.7-77.4)	54.5 (50.3-58.4)	66.8 (63.5-70.4)	73.7 (69.6-77)	80.7 (75.5-85)
Guatemala	46.1 (40.7-51.9)	55 (49.5-60.4)	63.5 (55.6-70.5)	72.5 (61.7-81.3)	67 (61.7-72.1)	74.4 (69.8-78.5)	80.5 (75.2-85.1)	86.2 (79.7-91)
Guinea	37.4 (33.3-41.8)	35.8 (32.3-39.5)	37.2 (32.5-41.8)	36.8 (29.7-44.5)	62.4 (58-66.5)	60.8 (57-64.4)	62.2 (56.8-66.8)	61.7 (53.3-69.3)
Guinea-Bissau	39.7 (24.8-56.6)	43.4 (35.6-51.2)	51 (44.7-57)	57.5 (42.4-70.7)	58.4 (41.5-73.5)	62.5 (55-69.5)	69.4 (64-74.6)	74.4 (61.7-84.4)
Haiti	31.5 (27.7-35.5)	36.3 (32.4-40.5)	40.7 (34.9-46.7)	46.6 (36.1-56.8)	53.8 (49.4-58.5)	59.1 (55.4-63.1)	63.4 (57.9-68.8)	68.6 (59.2-76.8)
Honduras	62.2 (55.6-68.2)	69.6 (65.6-73.3)	74.7 (67.3-81.1)	80.0 (74.1-84.9)	76.4 (71.4-80.8)	81.9 (79.2-84.4)	85.3 (80.6-89.4)	88.7 (84.9-91.8)
India	43.6 (38.9-48.4)	53.8 (49.5-58)	64.7 (58.5-70.3)	76.7 (67-84.6)	64.9 (60-69)	73.6 (69.9-76.8)	81.4 (76.9-85.1)	88.7 (83.1-92.8)
Indonesia	46.6 (41.1-51.9)	51.3 (47.3-55.3)	56.7 (51.9-61.8)	60.8 (52.6-68.8)	73 (68.6-76.9)	76.5 (73.7-79.2)	80.2 (76.9-83.3)	82.7 (77.3-87.3)
Iraq	60.4 (45-74.5)	59.2 (54.6-63.8)	59.8 (48.5-70.9)	61.8 (46.3-76.7)	70.9 (57.2-82.6)	70 (66.2-73.7)	70.5 (60-79.2)	72.1 (56.9-83.9)
Jordan	58.8 (52.3-65.1)	69.2 (65-72.7)	63.3 (57.7-68.7)	71 (62.3-77.9)	67.9 (62.2-73.2)	76.9 (73.6-79.9)	71.8 (66.6-76.4)	78.3 (71.6-83.9)
Kazakhstan	56.5 (32.5-78.1)	75.3 (54.4-90.1)	83.6 (66-94.2)	89.5 (74.7-97)	70.6 (64.4-76.3)	85.5 (81.5-88.8)	90.9 (85.4-94.6)	94.3 (88.2-97.7)
Kenya	41.8 (37.4-46.2)	48.9 (45.1-52.9)	58.7 (52.1-65.3)	72.4 (61.4-81.5)	67.6 (63.6-71.6)	73.6 (70.5-76.7)	80.5 (76-84.6)	88.3 (81.8-93)
Lesotho	47.2 (41.3-53.3)	59.1 (55.4-62.7)	64.8 (58.3-70.4)	69.1 (57.5-79)	64.9 (59.6-70.2)	75 (72.1-77.8)	79.2 (74.7-83)	82.2 (73.6-88.7)
Madagascar	29.2 (24.4-34.2)	32.5 (28.6-36.4)	35.9 (30.6-41.7)	42.1 (33.8-50.3)	58.5 (52.4-64.2)	62.3 (58.3-66)	65.7 (60-71)	71.2 (63.2-77.8)
Malawi	54.4 (50.4-58.4)	64.2 (61.2-67.1)	74.8 (71.7-77.6)	82.0 (78.1-85.5)	67.3 (63.4-70.8)	75.6 (73-78)	83.6 (81.4-85.7)	88.7 (85.9-91)
Maldives	75.5 (64.3-84.1)	78.2 (73-82.7)	80.1 (70.1-87.4)	78.3 (63.8-88.7)	67.5 (53.4-78.9)	70.7 (65.3-75.7)	73.1 (60.3-82.7)	71.2 (56.6-82.5)
Mali	30.4 (26.5-34.4)	36.1 (32.8-39.4)	40.7 (36.6-45)	45.7 (39.1-52.4)	57 (52.7-61.1)	63.2 (59.5-66.9)	67.6 (63.6-71.3)	71.8 (65.7-77.1)
Mauritania	29.2 (17.7-43.2)	37.1 (31.3-43.4)	47.4 (39.2-56.4)	59.0 (36.1-79)	47.5 (33-61.8)	56.8 (50.9-62.4)	66.7 (58.1-74.7)	75.6 (54.6-89.3)
Mexico	59.3 (38.9-78.9)	64.5 (54.7-73.3)	68.2 (59.3-76.7)	71.6 (51-86.7)	75.9 (58.2-89.6)	80.1 (72.3-87.1)	82.7 (76.3-88.2)	84.7 (70.9-93.2)
Mongolia	70 (61.1-77.8)	72.6 (68.5-76.6)	75.8 (71.5-80)	79.2 (70.3-86.7)	76.9 (68.9-83.6)	79.1 (75.6-82.5)	81.7 (78.2-85)	84.5 (77.5-89.8)
Montenegro	56.9 (27.4-82.8)	45.8 (13.7-78.7)	45.4 (9.3-82.5)	35.6 (0.6-92.1)	74.6 (42.9-95.1)	67.4 (59.2-75)	66.5 (54-77.7)	49.1 (7.0-90.0)
Morocco	53.3 (37.5-68.3)	63.2 (35.3-86.5)	67 (24.5-94.9)	68.2 (11.9-99)	67.9 (54.7-78.7)	75.3 (46.7-92.8)	77.3 (36.2-97.4)	76.6 (16.9-99.5)
Mozambique	43.2 (37.3-49.4)	41.5 (36.1-46.5)	51.6 (42.3-60.2)	57.9 (42.8-71.8)	75.5 (70.6-79.9)	74.2 (70.2-77.9)	81.1 (74.8-86.1)	84.5 (74.8-91.2)

Myanmar	24.1 (4.6-61.7)	34 (17.3-56.5)	60.3 (42.9-74.3)	63.9 (42.7-82)	47.8 (15-85.3)	63 (44-81.8)	83.6 (71.2-91.3)	85.5 (71-94.2)
Namibia	55.3 (50.1-60)	59.9 (55.9-64)	65.5 (52.6-76.2)	70.1 (50-85.4)	76.9 (73.2-80.6)	80.1 (77.4-83)	83.5 (74.5-90)	85.9 (72.5-94.2)
Nepal	43.6 (39.5-47.8)	53.9 (51-56.8)	66.3 (62.5-69.7)	75.2 (70.1-79.5)	55.2 (50.9-59.4)	65.2 (62.5-67.7)	75.8 (72.7-78.6)	82.9 (79-86.1)
Nicaragua	62.9 (55.7-69.5)	68 (55.5-79)	71.6 (44.8-89)	72.6 (30.2-95.2)	81.3 (76.5-85.2)	84.3 (75.8-91.1)	85.9 (67.7-95.9)	85.6 (52.8-98.2)
Niger	21.5 (18.2-25.2)	32.7 (28.7-37)	46.5 (38.5-55.1)	61.7 (49.5-73.3)	56.6 (51.3-61.4)	69.8 (65.8-73.6)	80.4 (75.1-85.2)	88.3 (82.4-92.7)
Nigeria	18.7 (16.4-21.2)	19.7 (17.6-21.8)	26.9 (22.3-31.5)	35.8 (25.5-46.4)	56 (52.4-59.6)	57.5 (54.3-60.7)	66.9 (61.6-71.7)	75.2 (66.1-82.9)
Pakistan	35.5 (24.4-46.9)	44.3 (40.2-48.4)	53.6 (47.6-59.4)	66.1 (54.7-77.2)	58.1 (46-68.9)	67 (63-70.8)	74.6 (69.9-79)	83.1 (75.3-89.9)
Panama	60.6 (30.9-84.6)	57.9 (45.5-70.1)	57.4 (45.6-68.1)	48.7 (18.5-79.6)	84.9 (62.2-96.3)	84.7 (75.9-91.4)	84.5 (77.2-89.8)	77.5 (49.8-94.1)
Paraguay	63.3 (33.2-88.1)	70.7 (57.1-82.2)	77.1 (70.5-83)	80.6 (62.2-92.1)	70.4 (39.3-91.4)	77.3 (63.8-87.2)	82.7 (77.1-87.1)	85.5 (71-94)
Peru	54.6 (51.7-57.6)	58.4 (56.5-60.2)	60.3 (56.7-63.9)	63.5 (58.5-68.1)	70.8 (68.3-73.1)	73.8 (72.5-75.2)	75.4 (72.4-78)	77.8 (73.7-81)
Philippines	29.4 (26.2-33.1)	34.8 (31.2-38.4)	42.8 (37.9-48.1)	54.9 (46.6-62.8)	63.5 (59.5-67.3)	69.1 (66.2-72)	75.7 (72-79.3)	83.5 (78.5-87.5)
Rwanda	29.8 (26.3-33.5)	51.3 (47.7-54.9)	69.7 (65.4-73.5)	83.9 (79.5-87.6)	44.5 (40-48.6)	66.5 (63.6-69.5)	81.3 (78.4-84.1)	90.8 (88.1-93)
Senegal	39.2 (33.9-44.5)	46.9 (43.9-49.7)	56.4 (53.2-59.5)	66.3 (59.2-73.1)	56.7 (50.8-62.2)	64.3 (61.4-66.9)	72.5 (69.8-75.1)	80.0 (74.9-84.7)
Sierra Leone	43.4 (31-55.2)	55.8 (52.1-59.4)	70.4 (66.3-74.1)	80.9 (75-86.2)	55 (42-67.3)	67 (63.8-70.1)	79.2 (76.1-81.9)	87.2 (82.9-90.8)
South Africa	69 (63.7-73.7)	70.7 (64.7-76.1)	73.7 (65-81)	75.3 (62-85.5)	75.4 (70.8-79.7)	76.9 (72.3-80.9)	79.4 (71.8-85.8)	80.6 (68.8-89.3)
Sudan	24.4 (11.3-43)	37.5 (32.8-42.8)	41.8 (30.6-55.3)	54.4 (36.4-72.5)	47.7 (29-68.5)	63.9 (58.5-69.2)	67.6 (56.1-78)	77.3 (62.5-88.7)
Tajikistan	53.8 (38.6-69)	61.5 (54.4-68.3)	68.8 (61.9-75)	74.2 (60.6-84.4)	58.5 (43.2-72.5)	66 (59.5-71.8)	72.8 (66.3-79)	77.7 (65.6-87.1)
Thailand	84.2 (68-94.3)	85.1 (80.7-88.6)	84.2 (78.7-89.1)	82.4 (63.2-93.3)	88.4 (75-96.1)	89.2 (85.6-92.2)	88.6 (84.2-91.8)	87.1 (71.7-95.2)
Timor-Leste	44 (29.6-58.3)	49.5 (44-54.9)	57.9 (48.6-66.6)	62.5 (43.9-79)	56.8 (42.1-70.4)	62.3 (57.1-67.3)	69.9 (61.4-78.2)	73.5 (57.5-86.6)
Togo	30.6 (26.4-35.1)	38.4 (34.2-42.4)	43.9 (38.7-49.3)	51.0 (41-60.5)	50.8 (45.8-56)	59.4 (55.6-63.4)	64.8 (59.7-69.6)	70.8 (62.4-78.3)
Uganda	36.9 (32.8-41.4)	49.2 (45.5-52.7)	60 (54.4-65.2)	71.2 (63.2-78.2)	56.5 (51.8-60.8)	68.3 (65-71.5)	76.9 (72.8-80.8)	84.5 (79.3-88.8)
Yemen	30.9 (14.6-48.8)	39 (28.9-49.5)	27.3 (8.2-59.8)	34.4 (9.6-72.5)	51.1 (29.4-69.7)	60.5 (47.8-71.7)	45.6 (19.4-75.5)	53.4 (22.3-84.9)
Zambia	47.3 (42.5-52.7)	52.9 (47.5-58)	66.1 (60.2-71.7)	73.9 (65-81.7)	68.4 (63.8-72.5)	73.1 (68.3-77.1)	82.5 (78.7-85.7)	87.2 (81.7-91.5)
Zimbabwe	39.4 (27.5-49.8)	53.6 (49.4-57.8)	72.1 (66.3-77.1)	84.5 (77.9-89.7)	54.9 (41.8-65.6)	68.6 (65.4-71.8)	83 (79.2-86.7)	91.1 (87.2-94.5)

CrI, credible interval

Table S10: Country-specific composite coverage index by wealth quintile, 2000-2030

	Poorest (%, 95% CrI)				Richest (%, 95% CrI)			
**country**	**2000**	**2010**	**2020**	**2030**	**2000**	**2010**	**2020**	**2030**
**Southern Asia**								
Afghanistan	18.8 (6.9-38.5)	30.3 (23-39)	43.6 (34.1-52.9)	57.2 (32.2-78.5)	35.5 (15.5-60)	51.8 (41.3-61.9)	65.6 (55-74.5)	76 (55.2-90.5)
Bangladesh	40.9 (37.6-44.4)	52.6 (49.6-55.4)	65.1 (61.4-68.6)	76.8 (69.1-83.3)	63 (59.5-66.3)	73.2 (70.7-75.7)	82.1 (79.3-84.6)	89 (84.1-92.7)
India	35.7 (31.9-39.8)	49 (45.3-53)	63.3 (57.9-69)	78.5 (70.8-85.1)	70 (66.2-73.5)	80.1 (77.3-82.8)	87.9 (84.9-90.4)	93.9 (91.1-96.1)
Maldives	70.9 (55.8-81.9)	72.7 (68.4-76.5)	76.7 (64.7-84.9)	71 (60.3-80.5)	69.7 (53.8-81.1)	71.5 (66.4-75.7)	75.5 (62.7-84.7)	69.8 (58.2-79.9)
Nepal	34.1 (31.1-37.2)	49.4 (46.8-52)	64.9 (61.2-68.2)	78.3 (74.8-81.4)	55.6 (52-59.2)	70.2 (68-72.5)	81.7 (79.1-83.9)	89.7 (87.6-91.3)
Pakistan	25 (14.9-40.1)	35.8 (31.9-39.5)	47.9 (42.8-52.8)	64.3 (52.4-74.6)	56 (40.9-71.8)	68.7 (64.9-72.2)	78.4 (74.6-81.7)	87.5 (80.5-92.1)
**South-eastern Asia**								
Cambodia	28.5 (25.5-31.5)	52.2 (49.1-55.6)	74.7 (69.8-79.1)	88.8 (84.7-92.2)	47.6 (43.5-51.9)	71.3 (68.2-74.3)	87 (83.8-89.6)	94.7 (92.5-96.4)
Indonesia	57.2 (52.1-62.3)	64.2 (60.2-67.6)	69.8 (65.9-73.5)	74.7 (68.7-80.1)	73.5 (69.4-77.4)	78.9 (76.1-81.7)	82.8 (80.1-85.4)	86 (82-89.5)
Myanmar	30.4 (6.4-66.8)	45.5 (21.1-74.7)	65 (51.8-77.6)	71.6 (49.3-88)	58.9 (21.7-87.7)	75.1 (52.6-91.9)	87.7 (79.2-93.5)	90.4 (79-96.7)
Philippines	47.4 (43.8-50.8)	55.1 (51.6-58.6)	62.3 (57.8-66.6)	68.4 (62.1-73.7)	69.6 (66.3-72.7)	75.7 (72.8-78.4)	80.7 (77.5-83.6)	84.6 (81-87.9)
Thailand	84.2 (70.1-93.3)	88.2 (85.2-90.9)	89.6 (85.9-92.6)	90.9 (81.7-96.5)	79.3 (61.8-90.9)	84.2 (79.9-87.6)	86 (81.3-89.8)	87.8 (76.7-95)
Timor-Leste	30.8 (15.5-49.9)	45.2 (40.7-49.8)	60.4 (49.1-71.6)	62.1 (39.9-80.6)	53.8 (32.9-72.6)	69.1 (64.6-73)	80.4 (72.1-87)	81.1 (64.6-92)
**Central Asia**								
Kazakhstan	67.2 (62.4-71.1)	83.2 (80.2-85.7)	89.8 (86.1-92.7)	93.9 (89.8-96.8)	74 (69.4-78)	87.3 (84.6-89.4)	92.4 (89.3-94.8)	95.5 (91.9-97.7)
Tajikistan	59.2 (45.2-72)	63.4 (56.9-69.2)	67 (60.8-72.7)	70.5 (58.4-80.4)	68.9 (54.9-80.7)	72.6 (66.8-78.1)	75.7 (70.4-80.5)	78.5 (69.3-86.6)
**Eastern Asia**								
Mongolia	74.9 (66-82.3)	77.8 (74.5-80.8)	79.4 (75.4-82.9)	81.5 (72.5-88.4)	79 (71.3-85.3)	81.6 (78.7-84.4)	83 (79.3-86.2)	84.8 (77-90.8)
**Western Asia**								
Armenia	46.6 (42.8-50.5)	60 (57.1-62.9)	73.8 (69.7-77.6)	83.6 (78.9-87.6)	61.1 (56.8-64.9)	72.9 (70.1-75.7)	83.5 (80.5-86.2)	90.1 (87-92.6)

Azerbaijan	78.5 (15.6-99.6)	11.5 (0.2-57.6)	17.4 (0.4-74.4)	17.3 (0-89.5)	86.5 (28.5-99.8)	19.1 (0.4-76.1)	27.2 (0.7-86.3)	24.8 (0.1-94.9)
Iraq	61.6 (30.7-86.6)	58.5 (53.2-63.4)	63 (48.6-74.9)	65.8 (46.8-81)	72.3 (43.9-91.7)	70.5 (65.6-74.9)	74.1 (60.9-83.8)	76.2 (58.6-87.9)
Jordan	63.6 (58.4-68.5)	72.2 (68.8-75.6)	71.8 (67.7-75.3)	77.9 (72.7-82.7)	70.7 (65.8-75)	78.2 (75-81.2)	77.8 (74.2-81.1)	83 (78.4-87)
Yemen	17.6 (6.7-35.5)	30.9 (22.8-39.5)	14.1 (4.8-32.2)	23.4 (5.7-56.7)	46.2 (22.8-71)	65.6 (54.9-74.9)	39.6 (18.2-65.2)	53.2 (21.7-84.3)
**Sub-Saharan Africa**								
Angola	83 (14.8-99.9)	39.8 (23.4-57.8)	78.3 (38.9-96.9)	83.4 (47.3-98.1)	93.2 (46.1-100)	74.4 (58.7-86.6)	93.3 (72.8-99.3)	95.2 (80.4-99.6)
Benin	35 (31.8-38.3)	36.8 (34.1-39.2)	40.8 (36.5-44.9)	51.6 (31.4-75.9)	58.5 (54.5-62.6)	60.4 (57.4-63.4)	64.3 (59.7-68.1)	72.8 (54.8-89.5)
Burkina Faso	17.9 (15.6-20.2)	39.9 (35.8-44)	67.2 (59.8-73.9)	85.7 (79.1-90.8)	45.8 (41.5-50.4)	72 (68.1-76)	88.7 (85.4-91.7)	95.8 (93.7-97.5)
Burundi	41.5 (30.7-52.3)	52.1 (47.1-56.7)	62.4 (55.8-68.4)	71.6 (60.1-81)	50.7 (39.5-61.2)	61.2 (56.3-65.7)	70.7 (64.4-76.6)	78.5 (68.8-86.6)
Cameroon	24.5 (21.9-27.3)	28.5 (25.7-31.1)	32.9 (28.8-37)	41.7 (36.3-47.2)	60.1 (56.2-63.5)	64.9 (61.4-67.8)	69.4 (65-73.3)	76.8 (72.1-81)
Central African Republic	18.2 (12.8-23.9)	20.1 (16.9-23.3)	20.5 (17.1-24.2)	24 (15.7-34.5)	53.3 (43.7-62.5)	56.5 (51.2-61.7)	57.1 (51.4-62.9)	61.6 (48.9-73.2)
Chad	4 (3.1-5.2)	9.7 (8.2-11.3)	20.4 (15.2-26.4)	38.8 (26.1-52.8)	19.1 (15.3-23.8)	37.6 (33.3-42)	58.8 (50.5-66.6)	77.5 (67.3-86.2)
Comoros	31.1 (16-49.9)	40.2 (33.9-46.6)	50.4 (35.5-64.6)	59.9 (31.4-83.3)	46.6 (27.4-68.2)	57.1 (50-64.3)	66.6 (52.5-78)	73.9 (46.3-91)
Democratic Republic of the Congo	31.4 (26.3-36.8)	33.2 (30.3-36.2)	34.3 (29.5-39.1)	34.6 (24.8-45.6)	54.7 (48.2-60.5)	56.8 (53.3-60.4)	57.9 (52.3-63.3)	58 (45.4-69.2)
Ethiopia	9.5 (8.2-10.8)	21.5 (18.7-23.9)	42.7 (37.4-48.4)	65.5 (57-73.8)	32.7 (29-36.6)	55.8 (51.9-60)	77.5 (73.4-81.4)	89.7 (86.1-93)
Gabon	35.4 (31-39.9)	48.1 (41.6-53.9)	52.6 (44.3-60.9)	52.3 (30.1-73)	53.7 (48.3-59.1)	66.2 (60.2-72)	70.1 (62.6-76.6)	69.2 (49.1-85.1)
Ghana	39.7 (36.6-42.9)	51.8 (48.1-55.7)	60 (55.9-63.8)	69 (62.8-74.3)	60.9 (57.4-64.5)	71.7 (68.5-74.7)	78 (74.7-80.8)	84 (80.1-87.3)
Guinea	22.5 (19.7-25.4)	24.7 (22.3-27)	30.3 (27-34)	35.3 (29.7-41.6)	56.1 (51.8-60.2)	59.1 (55.6-62.4)	65.7 (61.4-69.8)	70.6 (64.7-76.2)
Guinea-Bissau	29.8 (16.2-48.1)	39.2 (32.2-46.7)	50.1 (44.4-55.6)	61.2 (46.6-73.7)	47.9 (30.1-65.9)	58.8 (51-66)	69 (63.3-74.1)	77.6 (65.8-86.5)
Kenya	41.6 (37.8-45.5)	50.4 (47.2-53.8)	62.7 (57.8-67.5)	79.2 (72.4-84.8)	68.8 (65.2-72.1)	75.8 (72.9-78.5)	83.8 (80.6-86.7)	92.2 (89-94.6)
Lesotho	52 (47.2-56.5)	61.8 (58.5-64.7)	67 (61.7-71.6)	70.7 (59.5-79.9)	68.7 (64.4-72.8)	76.7 (74.2-79.3)	80.5 (76.8-83.7)	82.9 (75.4-88.6)
Madagascar	26.8 (22.8-30.9)	34.5 (31.1-38.1)	38 (33.8-42.5)	46.8 (40.5-53.7)	59.7 (54.1-64.8)	68 (64.1-72.1)	71.3 (66.8-75.8)	78 (72.8-83)
Malawi	50.9 (46.9-54.5)	64.1 (61.3-67)	76 (73.4-78.4)	84.5 (81.5-87)	63.5 (59.8-66.9)	75 (72.6-77.4)	84.2 (82.1-86.2)	90.2 (88.1-92)
Mali	18.3 (16.1-20.7)	27.5 (24.9-30.4)	34.9 (31.5-38.1)	44.8 (39.6-50)	47.7 (43.8-51.3)	60.7 (57.3-64.1)	68.6 (65.3-71.9)	76.7 (72.6-80.7)

Mauritania	17.3 (9.8-26.9)	26.1 (21.9-30.4)	37.2 (29.1-45.4)	58.7 (35.5-79.9)	46.1 (31.2-60.5)	59.7 (54-65)	71.2 (63.2-77.9)	84.9 (69.9-94.5)
Mozambique	32.4 (27.8-37)	36.9 (32.7-41.1)	54 (45.7-61.7)	66.6 (53.1-77.6)	66.6 (61.8-71.4)	70.9 (66.6-74.8)	82.9 (77.9-87.2)	89.1 (82.4-93.5)
Namibia	58.8 (54.7-62.8)	66.4 (62.7-69.8)	71.9 (58.7-83.5)	76.3 (55.5-91.7)	79.9 (76.8-82.7)	84.6 (82.2-86.8)	87.5 (79.7-93.6)	89.6 (77.3-96.9)
Niger	11.5 (9.8-13.3)	25.2 (22.4-28.1)	48.3 (41.9-55.2)	71.1 (63-78.5)	39.8 (35.3-44.3)	63.1 (58.9-67)	82.6 (78.1-86)	92.6 (89.5-94.8)
Nigeria	9.5 (7.9-11.4)	16.5 (14.8-18.3)	22.5 (18.7-26)	33.8 (24.1-42.6)	50.6 (45.7-55.6)	65.9 (62.9-68.9)	73.8 (69.3-77.5)	83 (75.7-88)
Rwanda	27.7 (24.7-30.6)	52.6 (49.3-55.7)	72.5 (69.2-75.4)	86.7 (83.6-89.3)	38.2 (34.5-41.9)	64.1 (60.6-67.3)	80.9 (78.1-83.4)	91.3 (89.1-93.2)
Senegal	27.6 (24.2-31.2)	37.4 (35.2-39.9)	50.6 (47.8-53.2)	67 (61.5-72.3)	52.2 (47.5-56.6)	63.2 (60.7-65.6)	74.7 (72.4-76.8)	85.3 (81.8-88.5)
Sierra Leone	30.2 (18-45.3)	51.5 (48.5-54.8)	70.7 (67.4-73.9)	84.7 (79.6-89.2)	42.3 (26.7-59.2)	64.6 (61.4-67.6)	80.5 (77.8-83)	90.5 (86.8-93.2)
South Africa	68.8 (64.7-73)	67.3 (62.2-72.2)	72.1 (64.6-78.8)	74 (62.9-83.2)	81.7 (78.4-84.7)	80.8 (77-84.3)	83.9 (78.2-88.6)	85.1 (76.5-91.3)
Togo	24.5 (21.4-27.7)	35.2 (32-38.6)	43 (38.7-47.3)	53.2 (45.5-60.7)	47.7 (43.3-52.5)	60.6 (56.6-64.3)	68.1 (63.7-72)	76.2 (70.1-81.6)
Uganda	35 (31.3-38.8)	48.8 (45.6-52)	62.6 (58-66.9)	74.1 (67.5-79.6)	54.9 (50.4-59.3)	68.3 (64.9-71.3)	79 (75.8-82.1)	86.5 (82.8-89.9)
Zambia	47.9 (43.7-52.3)	53 (48.3-57.3)	70 (65.2-74.7)	79.3 (72-85.2)	71.8 (67.9-75.3)	75.7 (72.1-79.1)	86.6 (83.9-88.9)	91.4 (88.1-94.2)
Zimbabwe	44.4 (34.5-53.5)	57.5 (53.9-61.1)	73.8 (68.7-78.4)	85.2 (79.7-89.5)	62.7 (52.5-71.6)	74.1 (71-77.2)	85.6 (82.5-88.3)	92.4 (89.5-94.7)
**Northern Africa**								
Egypt	61.9 (58.3-65.5)	70.2 (66.6-73.7)	76 (71.8-79.8)	82.5 (75.5-88.2)	76.7 (73.7-79.6)	82.7 (79.8-85.2)	86.5 (83.4-88.9)	90.5 (86-93.8)
Morocco	43 (30.4-55.6)	62.9 (36.6-81.7)	72.8 (34.7-93.5)	80.3 (25.3-98.8)	67 (56.6-77.3)	81.4 (60.3-93.2)	86.7 (58.3-97.7)	90 (48.4-99.6)
Sudan	12.9 (5.3-24.7)	30.4 (26.5-34.2)	35.6 (26.4-46)	56.9 (36.8-75.7)	37.6 (19.2-56.6)	65 (60.2-69.8)	69.9 (60.2-78.6)	84.3 (7 1.3-92.9)
**Latin America and the Caribbean**								
Colombia	61 (56.6-65)	71.7 (68-75.4)	79.4 (73-84.6)	86.6 (79.4-91.5)	76.3 (72.9-79.5)	83.9 (81.2-86.2)	88.8 (85.2-91.9)	93 (89.3-95.6)
Costa Rica	79.7 (72.1-85.8)	81.3 (77.8-84.3)	82.2 (78.5-85.7)	83.1 (76.3-88.3)	84.5 (78.2-89.7)	85.8 (82.9-88.4)	86.6 (83.1-89.5)	87.2 (81.7-91.5)
Dominican Republic	72.1 (69.3-74.9)	75.2 (72.9-77.4)	78 (75.4-80.4)	78.3 (73.2-82.7)	75.5 (72.6-77.9)	78.4 (76-80.6)	80.9 (78.4-83.1)	81.1 (76.8-85)
El Salvador	49.3 (8.7-93.1)	71.1 (46.6-89.5)	87 (81-91.3)	93.9 (58.7-99.9)	56.1 (11.7-95.4)	77.4 (55.6-92.7)	90.5 (86.1-93.8)	95.5 (69-99.9)
Guatemala	38.6 (33.9-43.3)	50.6 (45.3-55.5)	62.6 (55.6-69)	73.9 (66.1-80.2)	69.7 (65.1-74.2)	78.9 (75.1-82.6)	85.9 (81.8-89.1)	91.2 (87.9-94)
Haiti	27.3 (24.2-30.5)	35.3 (31.3-39.4)	43.9 (38.8-49.1)	53.4 (42.9-63)	52.9 (48.5-56.8)	62 (57.5-66)	70.1 (65.3-74.1)	77.3 (69.2-83.4)

Honduras	63.9 (58.7-68.8)	71.9 (68.5-75.1)	78.1 (71.3-84)	82.3 (77.6-86.6)	77 (73-80.8)	82.9 (80.2-85.1)	87.1 (82.7-90.7)	89.8 (86.8-92.5)
Mexico	65.6 (42.2-84.3)	72.7 (64.2-80.2)	78.3 (71-84.4)	82.3 (64.6-93)	76.2 (55.1-90.4)	82 (74.2-87.9)	86.1 (80.2-90.6)	88.7 (76-95.8)
Nicaragua	65.2 (59.3-70.8)	71.1 (57.8-81)	75 (49.5-91)	77.2 (39.1-95.7)	82.3 (78.2-85.8)	85.7 (77.3-91.9)	87.6 (70.3-96.4)	88.3 (62.4-98.3)
Panama	61.5 (29-85)	68.6 (55.4-77.9)	74.4 (63.2-83.1)	77.2 (48-94.4)	77.9 (50.3-92.9)	83.7 (75.1-89.8)	87.2 (80.3-92.2)	88.4 (69.1-97.5)
Paraguay	60.7 (29.4-87.5)	71.8 (59.1-83.1)	80.5 (73.8-85.3)	85.6 (66.9-95)	64.6 (33.7-89.1)	75.4 (62.2-86.1)	83.3 (78-87.9)	87.8 (73.9-95.8)
Peru	56.1 (53.8-58.9)	61.1 (59.4-62.7)	63.4 (60.2-66.5)	67.4 (63.5-71.4)	75.1 (73.1-77.1)	78.8 (77.6-80)	80.3 (78.3-82.3)	83 (80.5-85.4)
**Southern Europe**								
Albania	63.4 (53.2-72.1)	54.5 (50.6-58.2)	55.1 (49.9-60.3)	48 (38.6-58.2)	76.5 (67.4-83.1)	69.3 (64.9-73.1)	69.8 (64.4-74.7)	63.5 (53.9-72.3)
Bosnia and Herzegovina	59.5 (20.3-88.7)	59.5 (53.9-65)	59.7 (30.8-83.9)	56.7 (7.3-96.8)	71.9 (31.6-93.9)	73.4 (67.7-78.6)	72.7 (44.2-91.1)	67.6 (12.6-98.4)
Montenegro	55.5 (26.1-81.7)	58.4 (50.2-66)	63.6 (50.2-75.3)	61.6 (25.3-89.7)	64.6 (33-87.8)	68 (60.1-75.3)	72.5 (60.8-82.2)	69.9 (34.4-92.4)

CrI, credible interval

Table S11: Country-specific education-based slope index of inequalities in CCI, 2000-2030

Country	Slope index of inequalities (95% CI)			
	2000	2010	2020	2030
**Southern Asia**				
Afghanistan	24.0 (10–38)	27.0 (19.2–34.8)	26.3 (23.9–28.6)	23 (22.2–23.7)
Bangladesh	21.9 (-21.7–65.5)	21.2 (-18.5–60.8)	18.3 (-14.4–51)	13.8 (-9.5–37.1)
India	32 (28.1–35.8)	29.7 (26.6–32.8)	25.1 (16.5–33.6)	18 (8.7–27.3)
Maldives	-12.0 (-26–2)	-11.3 (-24.5–2)	-10.5 (-21.4–0.4)	-10.7 (-23.9–2.6)
Nepal	17.4 (-1.3–36.1)	16.9 (0.6–33.3)	14.3 (2.6–25.9)	11.6 (3–20.1)
Pakistan	33.9 (29.2–38.6)	34.1 (31.7–36.4)	31.5 (22.2–40.8)	25.5 (11.5–39.5)
**South-eastern Asia**				
Cambodia	27.5 (6.4–48.5)	27.5 (15.8–39.1)	18 (18–18)	9.1 (6.8–11.5)
Indonesia	39.6 (5.4–73.8)	37.8 (-1.1–76.7)	35.2 (-4.4–74.9)	32.8 (-6.8–72.5)
Myanmar	35.6 (17.7–53.4)	43.5 (35.7–51.3)	35 (12.4–57.5)	32.4 (9.1–55.7)
Philippines	51.2 (27–75.3)	51.5 (16.4–86.5)	49.3 (0.3–98.4)	42.9 (-14.7–100.5)
Thailand	6.3 (4.7–7.9)	6.1 (5.4–6.9)	6.6 (6.6–6.6)	7 (6.3–7.8)
Timor-Leste	19.2 (17.6–20.8)	19.2 (16.1–22.3)	18 (13.3–22.7)	16.5 (11.8–21.2)
**Central Asia**				
Kazakhstan	21.2 (14.1–28.2)	15.3 (12.2–18.4)	11 (7.1–14.8)	7.2 (4.1–10.3)
Tajikistan	7.1 (-56–70.1)	6.8 (-53.2–66.7)	6 (-48.5–60.5)	5.3 (-42.2–52.7)
**Eastern Asia**				
Mongolia	10.4 (8–12.7)	9.7 (7.4–12.1)	8.8 (6.5–11.2)	8 (5.6–10.3)
**Western Asia**				
Armenia	28.5 (-4.2–61.2)	29.7 (9.5–49.9)	24.4 (15.9–33)	16.5 (14.9–18.1)
Azerbaijan	7.4 (-10.5–25.2)	7.3 (-12.1–26.8)	8.6 (-14–31.1)	6.6 (-10.5–23.7)
Iraq	15.8 (5.6–25.9)	16.2 (5.3–27.1)	16.1 (5.9–26.2)	15.5 (6.9–24)
Jordan	13.7 (-2.7–30)	11.6 (-1.7–24.8)	12.8 (-2–27.5)	11 (-2.3–24.2)
Yemen	30.3 (13.2–47.4)	32.3 (20.6–43.9)	27.5 (9.6–45.3)	28.5 (14.5–42.5)
**Sub-Saharan Africa**				
Angola	13.7 (11.3–16)	37.5 (32.8–42.2)	22 (15–29.1)	19.8 (10.5–29.1)
Benin	25.8 (24.2–27.4)	26 (25.2–26.7)	26 (23.6–28.3)	23.7 (20.6–26.8)
Burkina Faso	46.8 (42.1–51.5)	45 (24.8–65.2)	35.7 (4.6–66.8)	25.2 (-4.4–54.8)
Burundi	19.7 (-2.9–42.2)	19.2 (-1–39.4)	17.9 (0–35.7)	16.1 (1.3–30.8)
Cameroon	47.1 (25.3–68.9)	48.3 (20.3–76.3)	48.9 (14.7–83.1)	48.5 (2.5–94.4)
CAR	41.6 (-7.5–90.6)	42.6 (-5.6–90.8)	42.5 (-6.6–91.5)	43.3 (-1–87.7)
Chad	34.1 (-5.6–73.7)	42 (4.7–79.3)	47 (18.2–75.7)	49.8 (35.8–63.8)
Comoros	18.6 (-4.7–41.9)	19.2 (-2.6–41)	18.6 (-1.6–38.8)	17 (0.6–33.3)
DRC	22.5 (-25.7–70.7)	23 (-26.1–72)	23 (-24.5–70.4)	22.7 (-24.8–70.1)

Ethiopia	50.4 (-38.3–139.1)	59.1 (-7.8–126)	55.2 (35–75.4)	42 (34.2–49.8)
Gabon	14.4 (-26.1–54.9)	14.3 (-23.9–52.4)	14.1 (-23.2–51.4)	13.5 (-22.3–49.3)
Ghana	19.5 (2.4–36.6)	18.6 (6.2–31)	16.9 (6.8–27.1)	14.1 (6.3–21.9)
Guinea	37.5 (31.3–43.7)	37.5 (29.7–45.3)	37.5 (29.7–45.3)	37.4 (30.3–44.4)
Guinea-Bissau	28.1 (24.2–31.9)	28.7 (26.3–31)	27.6 (24.5–30.7)	25.4 (19.9–30.8)
Kenya	38.7 (23.1–54.3)	37 (30–44.1)	32.7 (31.1–34.3)	23.9 (15.3–32.4)
Lesotho	26.6 (2.4–50.7)	23.9 (6–41.7)	21.6 (9.2–34)	19.7 (9.5–29.8)
Madagascar	44 (29.2–58.7)	44.7 (36.9–52.5)	44.7 (41.6–47.8)	43.7 (38.2–49.1)
Malawi	19.4 (-4.8–43.5)	17.1 (-3.1–37.3)	13.2 (-0.8–27.2)	10 (-0.1–20.2)
Mali	39.9 (5.7–74.1)	40.7 (11.9–69.4)	40.3 (17.8–62.9)	39.2 (22.8–55.5)
Mauritania	27.5 (11.1–43.8)	29.6 (17.9–41.2)	28.9 (23.5–34.4)	24.9 (24.9–24.9)
Mozambique	48.5 (30.6–66.3)	49 (29.6–68.5)	44.3 (38.8–49.7)	39.9 (38.3–41.5)
Namibia	32.4 (29.3–35.5)	30.3 (30.3–30.3)	27 (23.9–30.1)	23.7 (19–28.4)
Niger	52.7 (16.1–89.2)	55.7 (47.1–64.2)	50.9 (31.4–70.3)	39.9 (5.7–74.1)
Nigeria	56 (36.5–75.4)	56.7 (34.9–78.5)	60 (13.3–106.7)	59.1 (-10.9–129.1)
Rwanda	22.1 (-13–57.1)	22.8 (-6.8–52.4)	17.4 (0.3–34.5)	10.4 (1.8–18.9)
Senegal	26.3 (-1–53.5)	26.1 (-3.5–55.7)	24.2 (-7.8–56.1)	20.5 (-9.8–50.9)
Sierra Leone	17.4 (14.3–20.5)	16.8 (10.6–23)	13.2 (7–19.4)	9.4 (4–14.9)
South Africa	9.6 (-27.7–46.9)	9.3 (-26.5–45.1)	8.6 (-24.9–42)	7.9 (-22.4–38.3)
Togo	30.3 (22.5–38.1)	31.5 (31.5–31.5)	31.4 (29–33.7)	29.7 (23.5–35.9)
Uganda	29.4 (-17.3–76.1)	28.7 (-11–68.3)	25.4 (-3.4–54.1)	20 (0.5–39.4)
Zambia	31.7 (7.5–55.8)	30.3 (11.6–49)	24.6 (15.3–33.9)	19.9 (14.5–25.4)
Zimbabwe	23.3 (-16.4–62.9)	22.5 (-10.2–55.2)	16.4 (-3.1–35.8)	9.9 (-1–20.8)
**Northern Africa**				
Egypt	14.6 (1.3–27.8)	12.6 (1.7–23.5)	10.8 (1.5–20.1)	8.4 (2.2–14.6)
Morocco	21.9 (18.8–25)	18.2 (17.4–18.9)	15.5 (14.7–16.2)	12.6 (12.6–12.6)
Sudan	35 (23.3–46.6)	39.6 (38–41.2)	38.7 (32.5–44.9)	34.4 (18–50.7)
**Latin America and the Caribbean**				
Colombia	28.4 (24.5–32.2)	23.4 (15.6–31.2)	18.5 (9.9–27)	12.3 (4.5–20.1)
Costa Rica	5.3 (-4.9–15.4)	5.6 (-6.1–17.2)	5.8 (-7.4–19.1)	6.4 (-6.8–19.7)
Dominican Republic	13.2 (8.5–17.9)	13.2 (8.5–17.9)	13.8 (7.6–20)	14.6 (10.7–18.4)
El Salvador	8.9 (-10.6–28.3)	8.9 (-10.6–28.3)	5.6 (-6.1–17.2)	3 (-3.2–9.2)
Guatemala	31.4 (13.5–49.2)	29.1 (16.7–41.5)	25.5 (19.3–31.7)	20.5 (19.8–21.3)
Haiti	33.5 (-6.2–73.1)	34.2 (-1.6–70)	34.1 (0.6–67.5)	33 (5–61)
Honduras	21.3 (21.3–21.3)	18.5 (17.7–19.2)	15.9 (14.3–17.5)	13 (10.7–15.4)
Mexico	24.9 (-3.1–52.9)	23.4 (-6.2–53)	21.8 (-7–50.5)	19.7 (-6–45.3)
Nicaragua	27.6 (12–43.2)	24.5 (8.1–40.8)	21.5 (6.7–36.2)	19.5 (8.6–30.4)
Panama	36.5 (4.5–68.4)	40.2 (4.4–76)	40.7 (5.6–75.7)	43.2 (23–63.4)

Paraguay	10.7 (-11.9–33.2)	9.9 (-10.3–30.1)	8.4 (-7.2–24)	7.4 (-7.4–22.1)
Peru	24.3 (-17.7–66.3)	23.1 (-15.8–62)	22.7 (-15.5–60.8)	21.5 (-13.6–56.5)
**Southern Europe**				
Albania	21.9 (15.7–28.1)	24.2 (20.3–28)	24.5 (22.1–26.8)	24.6 (23–26.2)
Bosnia	19.8 (18.2–21.4)	26.9 (21.4–32.3)	25.8 (13.4–38.2)	18.3 (5.9–30.7)
Montenegro	26.5 (18–35.1)	32.4 (16.8–48)	31.7 (10.6–52.7)	20.3 (-3.9–44.4)

CI, confidence interval

Table S12: Country-specific wealth-based slope index of inequalities in CCI, 2000-2030

**Country**	Slope index of inequalities (95% CI)			
	2000	2010	2020	2030
**Southern Asia**				
Afghanistan	20.1 (13.8–26.4)	26 (19.4–32.5)	26.6 (22.1–31.1)	22.8 (20–25.5)
Bangladesh	25.7 (12.4–39)	24.1 (13.1–35)	19.9 (12.3–27.5)	14.3 (9.6–19)
India	42.5 (41–43.9)	38.5 (34–42.9)	30.3 (23.9–36.7)	18.9 (13–24.7)
Maldives	-1.3 (-4.1–1.5)	-1.3 (-3.9–1.2)	-1.3 (-3.8–1.2)	-1.3 (-4.1–1.5)
Nepal	26.1 (20.9–31.2)	25.3 (22.2–28.4)	20.4 (18.4–22.4)	13.8 (12.3–15.4)
Pakistan	38.4 (35–41.8)	40.8 (39.3–42.2)	37.7 (32.9–42.5)	28.5 (21.7–35.4)
**South-eastern Asia**				
Cambodia	23.2 (20.1–26.3)	23.2 (20.6–25.8)	14.9 (12.3–17.5)	7.1 (5.5–8.8)
Indonesia	20.7 (6.3–35)	18.5 (5.3–31.6)	16.3 (4.3–28.2)	14.2 (3.8–24.5)
Myanmar	34.9 (29–40.8)	36.4 (34.1–38.6)	27.8 (23.7–31.9)	23 (19.2–26.8)
Philippines	28 (15.5–40.4)	25.8 (13.2–38.4)	23 (11–35)	20.2 (9.2–31.1)
Thailand	-5.2 (-13.4–3)	-4.3 (-10.9–2.4)	-3.8 (-9.7–2)	-3.3 (-8.8–2.2)
Timor-Leste	28.1 (23.3–32.8)	29.2 (26.3–32)	24.4 (22.3–26.5)	23.2 (21.1–25.3)
**Central Asia**				
Kazakhstan	8 (5.2–10.8)	4.8 (3–6.6)	3.1 (2–4.2)	1.8 (0.9–2.8)
Tajikistan	12 (7.4–16.6)	11.5 (6.9–16)	10.8 (6.6–15)	9.9 (5.8–14)
**Eastern Asia**				
Mongolia	4.1 (0.4–7.8)	3.8 (0.4–7.2)	3.6 (0.3–6.9)	3.3 (0.3–6.3)
**Western Asia**				
Armenia	18.2 (14.06–22.3)	16.2 (11.7–20.6)	12.2 (8.4–15.9)	8.1 (5.3–10.9)
Azerbaijan	9.6 (8.1–11.1)	9.2 (7.6–10.8)	11.9 (10.2–13.5)	9.1 (7.7–10.4)
Iraq	12.9 (10.5–15.2)	14.5 (11.9–17)	13.3 (10.7–15.9)	12.5 (10–15)
Jordan	8 (5.3–10.8)	6.8 (4.5–9.1)	6.8 (4.5–9.1)	5.8 (3.9–7.7)
Yemen	35.2 (27.6–42.7)	42.8 (39.6–46)	31.3 (23–39.5)	36.7 (30.9–42.4)
**Sub-Saharan Africa**				
Angola	12 (9.1–14.9)	40.7 (26.6–54.8)	17.7 (14–21.3)	13.9 (11.1–16.7)

Benin	28.5 (25.1–31.8)	28.6 (25.2–31.9)	28.5 (25.4–31.5)	25.6 (22.4–28.8)
Burkina Faso	31.9 (7.8–55.9)	37.3 (17.8–56.8)	25.2 (16.9–33.5)	11.9 (9–14.8)
Burundi	10.8 (7.5–14.1)	10.7 (7.9–13.5)	9.7 (7.2–12.3)	8.1 (6.1–10.1)
Cameroon	44.5 (43.2–45.7)	45.5 (42.3–48.6)	45.6 (40.2–50.9)	43.7 (34.8–52.6)
CAR	42 (21.2–62.8)	43.7 (23.4–64)	44 (23.6–64.3)	45.3 (26.6–64)
Chad	17.2 (2.1–32.2)	32.1 (8–56.1)	44.7 (19.6–69.8)	45.6 (30.4–60.8)
Comoros	20.1 (12.1–28)	21.8 (12.5–31.1)	20.8 (11.1–30.4)	17.9 (9.2–26.6)
DRC	27.9 (19.3–36.5)	28.3 (19.6–36.9)	28.3 (19.9–36.7)	28 (19.6–36.4)
Ethiopia	25.9 (-0.6–52.3)	38.9 (6.2–71.6)	40.2 (15.8–64.5)	28.2 (16.5–40)
Gabon	23.7 (12.4–34.9)	23.3 (11.2–35.3)	22.4 (10.5–34.3)	21.7 (10.4–32.9)
Ghana	26.2 (24.9–27.4)	24.6 (22.6–26.5)	22.2 (19.9–24.5)	18.5 (15.6–21.3)
Guinea	41.4 (31.3–51.5)	42.5 (33.3–51.6)	43.8 (37–50.5)	43.7 (38.9–48.4)
Guinea-Bissau	22.1 (17.2–26.9)	23.9 (19.7–28.1)	23.1 (20.2–25.9)	20 (18.1–21.9)
Kenya	33.5 (30.4–36.6)	31.3 (26.5–36)	25.9 (20.1–31.7)	15.9 (10.8–20.9)
Lesotho	20.8 (17.5–24.1)	18.5 (14.8–22.2)	16.8 (13.2–20.3)	15.2 (11.8–18.5)
Madagascar	40.3 (32–48.5)	41.1 (35.7–46.5)	40.9 (36.9–44.8)	38.3 (36–40.6)
Malawi	14.7 (9.8–19.5)	12.7 (9–16.4)	9.5 (6.9–12.2)	6.6 (5–8.3)
Mali	34.7 (13.5–55.8)	39.5 (19.6–59.3)	40.2 (22.8–57.7)	38.3 (25.4–51.2)
Mauritania	36.5 (34.6–38.4)	42.5 (39.2–45.8)	42.9 (35.4–50.4)	32.8 (22.6–42.9)
Mozambique	42.5 (37.7–47.3)	42.3 (39.2–45.3)	36 (33.4–38.5)	27.9 (23.9–31.9)
Namibia	25.6 (22.2–28.9)	22 (18.6–25.5)	18.9 (15.5–22.2)	16 (12.9–19.2)
Niger	31.8 (0.2–63.3)	43.2 (9.1–77.4)	39.9 (18.5–61.2)	25.2 (16.1–34.3)
Nigeria	50.6 (31.2–69.9)	61.4 (48.4–74.4)	64 (57.6–70.4)	61.4 (56.9–65.9)
Rwanda	12.3 (7.1–17.5)	13.5 (8.8–18.2)	9.9 (7.1–12.7)	5.4 (4–6.8)
Senegal	30.8 (29.7–31.8)	32.2 (28.8–35.5)	30 (24.6–35.4)	22.7 (16.5–28.8)
Sierra Leone	14.5 (10–19)	15.8 (12.1–19.4)	11.8 (9.7–13.9)	7 (5.7–8.2)
South Africa	15.9 (13.6–18.3)	16.7 (14.3–19)	14.6 (12.3–16.9)	13.7 (11.5–15.9)
Togo	28.5 (21.6–35.4)	31.2 (26.1–36.3)	30.9 (27.3–34.4)	28.3 (26.4–30.2)
Uganda	23.2 (9.6–36.7)	22.8 (11.3–34.2)	19.2 (10.7–27.7)	14.6 (8.8–20.3)
Zambia	29.1 (21.1–37)	27.7 (21–34.3)	20.3 (17–23.5)	14.8 (13–16.6)

Zimbabwe	22.2 (19.4–25)	20.2 (18.4–22)	14.3 (12.9–15.8)	8.7 (7.4–10)
**Northern Africa**				
Egypt	18.3 (13.6–23)	15.5 (11–19.9)	13 (9.1–16.9)	9.8 (6.6–13.1)
Morocco	29.7 (26.4–33)	22.9 (17.9–27.8)	17.1 (12.8–21.4)	11.9 (9–14.8)
Sudan	30.9 (26.5–35.2)	43.3 (41–45.6)	42.9 (38.5–47.2)	34 (25.2–42.8)
**Latin America and the Caribbean**				
Colombia	19.2 (6.3–32.1)	15.3 (4.3–26.2)	11.7 (3.4–20)	8 (2.1–13.8)
Costa Rica	5.6 (3.7–7.4)	5.3 (3.6–6.9)	5.1 (3.4–6.8)	4.7 (3.1–6.4)
Dominican Republic	4.2 (-3.3–11.6)	3.9 (-2.9–10.6)	3.6 (-2.7–9.8)	3.4 (-2.8–9.6)
El Salvador	7.9 (6.1–9.8)	7.4 (5.5–9.2)	4 (2.8–5.3)	1.9 (1.4–2.4)
Guatemala	38.2 (35.7–40.7)	34.8 (31.7–37.8)	28.6 (24.2–33)	21.1 (16.6–25.7)
Haiti	31.3 (26.4–36.2)	32.8 (29.6–35.9)	32.1 (29.9–29.9)	29.3 (27–31.6)
Honduras	16.1 (10.6–21.6)	13.5 (8.6–18.4)	11 (6.8–15.2)	9.1 (5.4–12.9)
Mexico	12.9 (11.6–14.2)	11.4 (10.4–12.3)	9.4 (8.4–10.5)	7.7 (6.8–8.7)
Nicaragua	21.1 (13.5–28.6)	18 (10.9–25)	15.5 (9.5–21.4)	13.6 (8.5–18.7)
Panama	21 (5.9–36)	19.1 (4.7–33.5)	16.2 (3.8–28.6)	14.2 (3.2–25.2)
Paraguay	4.6 (1.3–7.9)	4.2 (1.1–7.4)	3.4 (0.9–5.8)	2.6 (0.7–4.5)
Peru	23.2 (15.9–30.5)	21.6 (14.5–28.7)	20.7 (13.4–27.9)	19 (12.2–25.8)
**Southern Europe**				
Albania	16.2 (11.1–21.3)	18.4 (13–23.7)	18.3 (13–23.5)	19.2 (14.2–24.2)
Bosnia	15.1 (13.2–16.9)	17 (14.5–19.4)	15.9 (13.9–17.8)	13.3 (11.7–14.8)
Montenegro	10.9 (4.4–17.3)	11.5 (4.7–18.2)	10.6 (4.3–16.9)	9.9 (4.1–15.7)

CI, confidence interval

References

1. Lim SS, Allen K, Bhutta ZA, et al. Measuring the health-related Sustainable Development Goals in 188 countries: a baseline analysis from the Global Burden of Disease Study 2015. *The Lancet* 2016; **388**(10053): 1813-50.
2. Global Burden of Disease Collaborative Network. Global Burden of Disease Study 2017 (GBD 2017) Socio-Demographic Index (SDI) 1950–2017. Seattle, United States: Institute for Health Metrics and Evaluation (IHME), 2018.
3. Gwatkin D, Rutstein S, Johnson K, Suliman E, Wagstaff A, Amouzou A. Socio-economic differences in health, nutrition, and population within developing countries: An overview. Country reports on HNP and poverty. Washington DC: World Bank, 2009.
4. Rutstein SO. The DHS Wealth Index: Approaches for rural and urban areas. DHS Working Papers No. 60. Calverton, Maryland: Macro International, 2008.
5. Rutstein SO, Johnson K. The DHS wealth index. DHS comparative reports no. 6. Calverton, Maryland: ORC Macro, 2004.
